# Multi-amplicon microbiome data analysis pipelines for mixed orientation sequences using QIIME2: Assessing reference database, variable region and pre-processing bias in classification of mock bacterial community samples

**DOI:** 10.1371/journal.pone.0280293

**Published:** 2023-01-13

**Authors:** Katherine A. Maki, Brian Wolff, Leonardo Varuzza, Stefan J. Green, Jennifer J. Barb

**Affiliations:** 1 Translational Biobehavioral and Health Disparities Branch, Clinical Center, National Institutes of Health, Bethesda, MD, United States of America; 2 National Institute of Nursing Research, National Institutes of Health, Bethesda, MD, United States of America; 3 Thermo Fisher Scientific, Waltham, MA, United States of America; 4 Genomics and Microbiome Core Facility, Rush University, Chicago, IL, United States of America; National Taiwan University, TAIWAN

## Abstract

Microbiome research relies on next-generation sequencing and on downstream data analysis workflows. Several manufacturers have introduced multi-amplicon kits for microbiome characterization, improving speciation, but present unique challenges for analysis. The goal of this methodology study was to develop two analysis pipelines specific to mixed-orientation reads from multi-hypervariable (V) region amplicons. A secondary aim was to assess agreement with expected abundance, considering database and variable region. Mock community sequence data (n = 41) generated using the Ion16S™ Metagenomics Kit and Ion Torrent Sequencing Platform were analyzed using two workflows. Amplicons from V2, V3, V4, V6-7, V8 and V9 were deconvoluted using a specialized plugin based on CutPrimers. A separate workflow using Cutadapt is also presented. Three reference databases (Ribosomal Database Project, Greengenes and Silva) were used for taxonomic assignment. Bray-Curtis, Euclidean and Jensen-Shannon distance measures were used to evaluate overall annotation consistency, and specific taxon agreement was determined by calculating the ratio of observed to expected relative abundance. Reads that mapped to regions V2-V9 varied for both CutPrimers and Cutadapt-based methods. Within the CutPrimers-based pipeline, V3 amplicons had the best agreement with the expected distribution, tested using global distance measures, while V9 amplicons had the worst agreement. Accurate taxonomic annotation varied by genus-level taxon and V region analyzed. For the first time, we present a microbiome analysis pipeline that employs a specialized plugin to allow microbiome researchers to separate multi-amplicon data from the Ion16S Metagenomics Kit into V-specific reads. We also present an additional analysis workflow, modified for Ion Torrent mixed orientation reads. Overall, the global agreement of amplicons with the expected mock community abundances differed across V regions and reference databases. Benchmarking data should be referenced when planning a microbiome study to consider these biases related to sequencing and data analysis for multi-amplicon sequencing kits.

## Introduction

**Fig 1:**
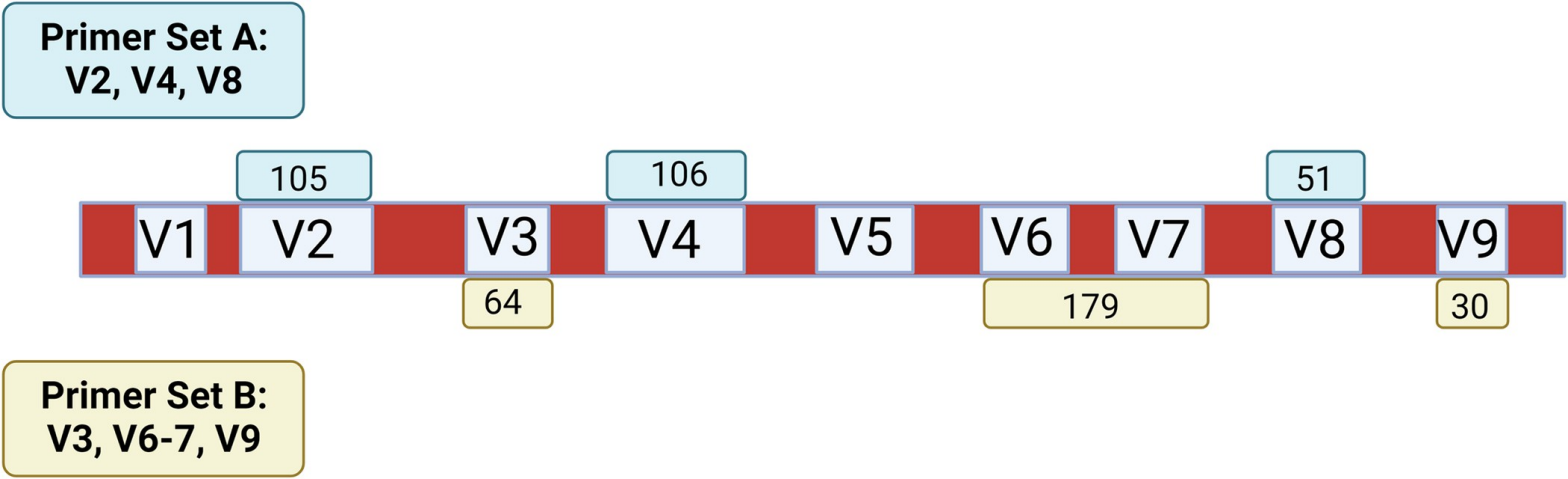
Variable regions of the 16S rRNA gene targeted and expected amplicon size. Schematic of the 16S rRNA gene and of the conserved regions (red) that are targeted by PCR primers to amplify the variable (V) regions (in blue). The Ion 16STM Metagenomics Kit (Thermo Fisher Scientific, Waltham, MA) used in this analysis includes two primer sets that targets 7 of the 9 V regions of the 16S rRNA gene: primer set A targets V2, V4, and V8 (green) and primer set B targets V3, V6-7, and V9 (yellow). The numbers inside each green and yellow primer location notation is the length of the V region targeted, as estimated by [1]. An exception is the V6-7 region, which also includes the conserved region sequence length in between V6 and V7 in the targeted amplicon. Note, the above numbers do not represent the length of the whole amplicon that is targeted by the primers, as that information is proprietary, but instead represent the length of the V region.

The length of the bacterial 16S rRNA gene, although slightly variable between different organisms, is approximately 1541 base pairs long (*e*.*g*., *E*. *coli*; [1]) and contains nine hypervariable (V) regions that are flanked by conserved regions (**Fig 1**). The structure of this gene makes it an ideal target for microbiome research, as the conserved regions of the gene are largely consistent across organisms from the domain Bacteria and therefore are used as targets for polymerase chain reaction (PCR) primers to extend across and amplify the V gene segment/segments of interest. The V regions that are amplified by polymerase chain reactions are subsequently sequenced and used to taxonomically classify bacteria based on their genetic signatures. PCR amplicons are sequenced using different platforms (i.e. Roche 454, Ion Torrent, Illumina, Oxford Nanopore and Pacific Biosciences) using single-end or paired-end reads to produce files used for downstream microbiome analysis. Illumina sequencing instruments have the capability to generate both single-end and paired-end sequences, while Ion Torrent sequencing platforms generally only create single-end, mixed orientation sequences. Furthermore, Illumina sequencing technology is highly used across institutions and sequencing cores and therefore many bioinformatics pipelines are tailored to the paired-end unidirectional sequences that result from the instrument. Consequently, documentation aimed at bioinformatics analysis using the mixed-orientation reads that result from Ion Torrent sequencing platforms, including the commonly used microbiome analysis workflow Quantitative Insights into Microbial Ecology version 2 [QIIME2; 2], is not as readily available for microbiome researchers to use as a resource. This can create challenges for researchers new to microbiome analysis as small differences in certain processing commands can result in drastically different output [3].

One important decision that researchers must make before beginning a microbiome study using 16S rRNA sequencing technology is which V region(s) to target for amplification [4]. Many different analytic strategies are available to annotate and characterize microbial communities and conclusion inference can be biased by the V region targeted [5], among other factors [4]. Different V regions can classify certain strains of bacteria more accurately or at a higher taxonomic resolution versus others due to redundancy in base pairs across several species at certain V regions [6]. Nevertheless, it is of common practice in microbiome studies to choose one to three V regions for PCR amplification with a single primer set. For example, the Human Microbiome Project used primers to amplify the V1-V3, V3-V5 and V6-V9 regions in their sequencing of fecal samples to represent the gut microbiome [7], while other protocols employ multi-V region primers to target several V regions across the 16S rRNA gene [8,9].

Although long-read sequencing technology (such as methods using PacBio and Oxford Nanopore sequencing platforms) can reliably quantify sequences at the species or strain level due to its production of reads long enough to sequence the entire 16S rRNA gene [10], this technology is not as readily available to microbiome researchers as Illumina or Ion Torrent sequencing methodologies at this time. Therefore, multi-amplicon kits have been developed in an attempt to amplify short, but multiple, V regions across the 16S rRNA gene. Some multi-amplicon panels specific for microbiome research include the xGen^TM^ 16S v2 and ITS1 Amplicon Panel (Integrated Data Technologies, Coralville, IA) and the Ion 16S^TM^ Metagenomics Kit (Thermo Fisher Scientific, Waltham, MA). These kits aim to alleviate bias that can be present from single primer sets [5] and while also potentially increasing specificity to annotate down to the species level. For example, regions V1-V3 are better for speciating the *Staphylococcus* species relative to V4, where no sequence variation between *S*. *aureus* and *S*. *epidermidis* is found [6]). Multi-amplicon amplification and sequencing overcomes limitations of single primer set bias and limited speciation capacity for single amplicons, which theoretically leads to increased taxonomic resolution when annotating features [9]. However, the multi-amplicon approach also comes with a unique set of challenges. For example, downstream data analysis becomes increasingly more complex when the researcher is presented with results from six or more V regions. Additionally, some manufacturers have chosen not to publish the primer sequences and therefore workflows that rely on known primer sequences to separate V regions cannot be employed. This prevents the user from using amplicon deconvolution tools currently available in QIIME2 (such as Cutadapt), as input of the primer sequences is a requirement for these tools to be employed. Previous research by Barb et. al [ref 11] developed a workaround for this by aligning amplicons to the 16s rRNA gene, while other researchers who have used the Ion 16S metagenomics Kit rely on the built-in Ion Reporter™ software (https://ionreporter.thermofisher.com/ir/). In the literature, other researchers reporting Ion Torrent microbiome sequencing results with known primers targeting a single V region used the original version of QIIME [12,13]. Although these are valid options, many users prefer the flexibility of open-source tools that are available within QIIME2 and want to have the option to separate reads by the respective V regions amplified when multi-amplicon kits are used. Additionally, support for QIIME is no longer maintained and the use of QIIME2 for microbiome analysis is continuously expanding and used by many researchers in the microbiome field [14–17].

To address the issue of proprietary primers preventing V-specific amplicon deconvolution for users of the Ion 16S^TM^ Metagenomics Kit, Thermo Fisher developed a plugin that uses the CutPrimers [18] pre-processing amplicon deconvolution workflow while masking the proprietary primers from the user performing the analysis. In this methodology manuscript, a microbiome analysis pipeline is presented that first employs this plugin developed by Thermo Fisher. After the plugin is run and sequences are separated by V region, each respective V region is imported into QIIME2 where the rest of the analysis pipeline continues.

As we understand that not all users of the Ion Torrent sequencing platform use the Ion 16S^TM^ Metagenomics Kit, we also present an alternate pipeline using the currently available QIIME2 Cutadapt [19] amplicon deconvolution workflow, with adjustments to the standard Illumina-based documentation that we obtained through extensive testing with Ion Torrent-generated sequences, along with benchmarking and quality control procedures in our own analyses. Importantly, this Cutadapt-based pipeline requires knowledge of the exact primer sequences for read trimming, extraction and deconvolution, and therefore cannot be used when a kit with masked proprietary primers are used [18].

To date, the commonly used QIIME2 documentation does not include a tutorial for 16S amplicon data in the mixed-orientation format, nor is there a standard workflow specific to Ion Torrent data. Because of this, there is a need for a comprehensive QIIME2 workflow aimed at unique considerations for Ion Torrent-specific mixed orientation single-end reads, especially those generated from the Ion16S^TM^ Metagenomics Kit. The aim of this project is to document two QIIME2 pre-processing microbiome analysis workflows specific to the single-end, mixed-orientation reads that result from Ion Torrent sequencing platforms: the first includes a new pre-processing plugin script that allows for amplicon deconvolution from sequences generated from the proprietary Ion 16S^TM^ Metagenomics Kit, and the second uses the established QIIME2 documentation. A nested, secondary aim was to investigate how well each V region (V2, V3, V4, V6-7, V8, V9) and three commonly used reference databases (Silva, Greengenes and RDP) perform when quantifying the global and taxon-specific bacterial composition of the mock samples.

## Methods

### Collection of mock community samples used for analysis

This manuscript outlines a workflow for collected and sequenced mock community samples and therefore does not contain any form of human data. Given that this work does not include any data from human participants, we were exempt from patient consent, ethics committee approval or Institutional Review Board approval. Sequence data from mock community DNA samples for this study were acquired from our prior studies [8,20] employing the Ion 16S^TM^ Metagenomics Kit (Thermo Fisher Scientific, Waltham, MA) and Ion Torrent Sequencing Platforms. Additional mock bacterial community sequencing data were identified by searching the literature for terms such as “Ion Torrent”, “Ion 16S”, and “Ion Torrent Multi-Amplicon Kit”. A list of studies using the Ion 16S^TM^ Metagenomics Kit was compiled and the sequence read archive was searched to find publicly available sequence files. Authors were contacted to verify that mock bacterial community samples were used in the study, and verify the DNA exaction methods, sequencing platform, and lot number of the mock bacterial sample. Additionally, a single FASTQ file containing sequence data from a mock bacterial community sample is available for download from the Ion Torrent Suite Software and used in this work. **Table 1** provides author and sample information for all mock bacterial community samples analyzed. Mock bacterial community samples generally fell into one of two distribution categories: samples that contained a uniformly distributed (usually 5%) relative abundance (RA) of each organism, while staggered samples contained RA values that range across taxa from 0.02% to >20%. For the remainder of this work, mock bacterial community samples that are evenly distributed will be deemed “even mock” and those that are staggered distribution will be deemed “staggered mock”. Even and staggered mocks from different companies (i.e. ATCC [Manassas, VA], BEI Resources [Manassas, Virginia], and Zymo Research [Irvine, CA]) were targeted to create heterogeneity with laboratory environment, if mock communities were purchased as genomic deoxyribonucleic acid (DNA) or cells that require DNA extraction, DNA extraction methodology (if applicable), sequencing methods and mock bacterial composition. Mocks were subsequently grouped into “mock type” categories, based on the manufacturer and expected bacterial RA for each sample (**Table 1**). Expected bacterial RAs for each mock type category are shown in **S1 Table**.

**Table 1:** Metadata and source of mock samples used.

Sample id	Even vs. Staggered Distribution	Manufacturer	Sequencing Platform	Total Reads	Reference
even_st_01	even	ATCC	Ion Torrent S5 XL	453,978	[20]
even_st_02	even	ATCC	Ion Torrent S5 XL	927,504	[20]
even_st_03	even	ATCC	Ion Torrent S5 XL	791,698	[20]
even_st_04	even	ATCC	Ion Torrent S5 XL	1,285,466	[20]
even_st_05	even	ATCC	Ion Torrent S5 XL	1,126,263	[20]
even_st_06	even	ATCC	Ion Torrent S5 XL	1,628,904	[20]
even_st_07	even	ATCC	Ion Torrent S5 XL	1,368,188	[20]
even_st_08	even	ATCC	Ion Torrent S5 XL	362,704	[20]
even_st_09	even	ATCC	Ion Torrent S5 XL	361,612	[20]
even_or_12	even	ATCC	Ion Torrent S5 XL	254,347	[8]
even_or_13	even	ATCC	Ion Torrent S5 XL	1,182,867	[8]
even_or_19	even	ATCC	Ion Torrent S5 XL	460,848	[8]
even_or_20	even	ATCC	Ion Torrent S5 XL	537,316	[8]
even_or_21	even	ATCC	Ion Torrent S5 XL	394,295	[8]
even_out_001	even	BEI	Ion Torrent PGM	306,935	[21]
even_out_006	even	ATCC	Ion Torrent PGM	267,880	[22]
even_out_007	even	ATCC	Ion Torrent PGM	291,418	[22]
even_out_008	even	ATCC	Ion Torrent PGM	300,469	[22]
even_out_009	even	ATCC	Ion Torrent PGM	251,620	[22]
even_out_012	even	BEI	Ion Torrent PGM	165,615	[23]
even_out_013	even	BEI	Ion Torrent PGM	185,338	[23]
stag_st_01	stag	ATCC	Ion Torrent S5 XL	461,147	[20]
stag_st_02	stag	ATCC	Ion Torrent S5 XL	832,836	[20]
stag_st_03	stag	ATCC	Ion Torrent S5 XL	360,847	[20]
stag_st_04	stag	ATCC	Ion Torrent S5 XL	1,378,356	[20]
stag_st_05	stag	ATCC	Ion Torrent S5 XL	1,188,743	[20]
stag_st_06	stag	ATCC	Ion Torrent S5 XL	1,591,533	[20]
stag_st_07	stag	ATCC	Ion Torrent S5 XL	1,403,778	[20]
stag_st_08	stag	ATCC	Ion Torrent S5 XL	829,750	[20]
stag_st_09	stag	ATCC	Ion Torrent S5 XL	355,202	[20]
stag_or_12	stag	ATCC	Ion Torrent S5 XL	337,113	[8]
stag_or_13	stag	ATCC	Ion Torrent S5 XL	950,954	[8]
stag_or_19	stag	ATCC	Ion Torrent S5 XL	611,115	[8]
stag_or_20	stag	ATCC	Ion Torrent S5 XL	599,866	[8]
stag_or_21	stag	ATCC	Ion Torrent S5 XL	430,075	[8]
stag_out_002	stag	BEI	Ion Torrent PGM	95,023	[24]
stag_out_003	stag	BEI	Ion Torrent PGM	306,748	[25]
stag_out_004	stag	Zymo	Ion Torrent PGM	157,630	[22]
stag_out_005	stag	Zymo	Ion Torrent PGM	151,707	[22]
stag_out_010	stag	BEI	Ion Torrent PGM	212,471	[23]
stag_out_011	stag	BEI	Ion Torrent PGM	153,684	[23]

Abbreviations. PGM: Personal Genome Machine. The mock bacterial distribution (evenly spaced versus staggered bacterial relative abundances) and manufacturer columns were used to create five “mock type” groups to categorize the expected relative abundances of the mock bacterial community samples (see **S1 Table** for more information and expected bacterial relative abundance).

### Bioinformatics pipeline using the CutPrimers-based MetagenomicsPP workflow before QIIME2 import (primer sequences are unknown)

**Fig 2:**
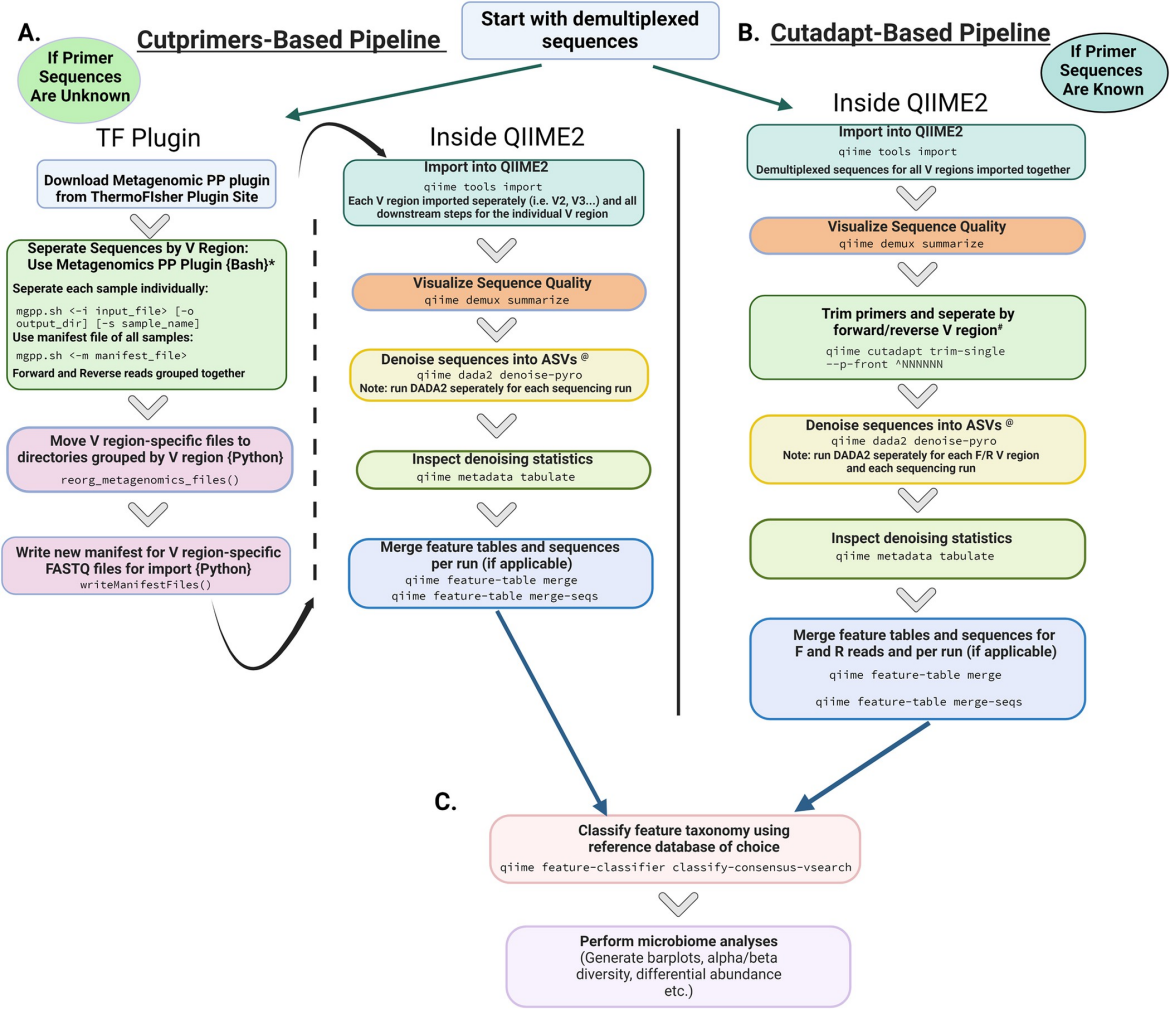
Microbiome bioinformatics pipeline by differing primer deconvolution method. **A**. Initial steps for the CutPrimers-based microbiome analysis pipeline. The steps on the left are performed outside of the QIIME2 environment using the TF plugin, where the demultiplexed FASTQ files are separated into respective V regions. These separated files are then imported into QIIME2 for denoising and feature/sequencing table merging, if needed. **B**. The Cutadapt-based microbiome analysis pipeline is performed exclusively in QIIME2. **C.** With both the CutPrimers-based pipeline and the Cutadapt-based pipeline, the feature taxonomy classification steps are the same and performed in QIIME2. Microbiome analysis methods such as alpha/beta diversity and differential abundance evaluations can be performed within or outside of the QIIME2 environment. Abbreviations. TF: Thermo Fisher Scientific. QIIME2: Quantitative Insights Insight Microbial Ecology, Version 2. *View MetagenomicsPP README for most current script syntax ^@^Can substitute to OTU clustering at this stage using other QIIME2 plugins ^#^Need primer sequences to run Cutadapt. For the workflow using the Ion 16S^TM^ Metagenomics Kit, run Cutadapt 12 times: 6 forward (V2F, V3F, V4F, V6-7F, V8F, V9F) and 6 reverse (V2R, V3R, V4R, V6-7R, V8R, V9R). Figure created with Biorender.

When primers sequences are unknown (due to the proprietary nature of the Ion 16S Metagenomics kit), the initial primer deconvolution is performed before importing sequencings into the QIIME2 environment (**Fig 2A**).

### MetagenomicsPP pre-processing plugin details

The proprietary forward and reverse primer sequences in the Ion 16S^TM^ Metagenomics Kit prevent the user from using primer-based amplicon deconvolution methods like cutPrimers or Cutadapt [17,18]. Therefore, authors KAM and JJB worked collaboratively with coauthor LV to develop a plugin for Ion 16S^TM^ Metagenomics Kit called the Metagenomics Post Processor (MetagenomicsPP). The MetagenomicsPP plugin separates amplicons and generates primer-based V region sub-folders to facilitate downstream 16S amplicon sequencing workflows such as QIIME2 [2], mothur [26], or custom microbiome analysis pipelines. The plugin can be run on the Torrent Suite software directly, or it can be downloaded to the user’s local computing environment from the Thermo Fisher Scientific plugin library [27]. The use of this plugin allows the researcher to deconvolute their amplicons by V region target without knowing the primer sequences a priori, as the primers are automatically provided to the CutPrimers workflow by the plugin functions, while keeping them hidden from the user. Please see **S1 Appendix** for MetagenomicsPP information and a Jupyter notebook with example scripts associated with the referenced analysis pipelines. As the MetagenomicsPP plugin employs the CutPrimers workflow for primer identification, trimming and removal, we refer to the analysis pipeline employing MetagenomicsPP as the CutPrimers-based pipeline (**Fig 2A**).

#### QIIME2 import, sequence denoising and taxonomy classification

In QIIME2, sequence files are imported as a QIIME2 artifact prior to preprocessing and denoising, if applicable [2]. FASTQ files separated into V specific reads were imported and processed using tools available in QIIME 2 (version 2020.8) (https://qiime2.org). Data were quality filtered and dereplicated with ‘q2-dada2’ using the ‘pyro’ flag to account for the Ion Torrent mixed orientation sequences [11]. Modifications to the DADA2 syntax were made to account for differences in reads generated from an Ion Torrent Sequencing platform versus those from an Illumina sequencing platform (**Fig 2A**). Because DADA2 models error individually per sequencing run while denoising, the DADA2 script was run separately for each sequencing run. After denoising, run-specific feature tables and representative sequences were merged to generate one master feature table and representative sequence list for each V region.

### Bioinformatics pipeline using Cutadapt within the QIIME2 environment (primer sequences are known)

When primers sequences are known (atypical when using the Ion 16S Metagenomics kit), the entire analysis pipeline can be employed within the QIIME2 environment (**Fig 2B**). Although this workflow is illustrated using a multi-amplicon kit from Ion Torrent, similar workflows can be employed using amplicons targeting single V regions or using other sequencing technology with some modifications to the pre-processing, denoising and taxonomy classification parameters.

#### QIIME2 import, V-specific sequence separation/adaptor removal and denoising

Demultiplexed FASTQ files were imported into QIIME2 as artifacts, and the Cutadapt plugin was implemented to split sequences based on known primer sequence into their targeted V regions (V2 forward, V2 reverse, V3 forward etc.; **Fig 2B**). Cutadapt allows for multiple adapter searching where all reads in the FASTQ file are searched for each primer input. This construct differs from the mutually exclusive workflow used by cutPrimers (i.e., as a primer is found, the read is removed from the pool to be searched). An important modification to the Cutadapt script in QIIME2, specific to nested multi-amplicon sequences is to use the “—p-front” parameter to ensure the script is only searching for sequences of an adapter ligated to the 5’ end and to also use the “^” symbol before the primer sequence so a primer is only matched if it is located at the beginning of the read. See **S1 Appendix** for example scripts and the documentation resource link. Reads were quality filtered and dereplicated with q2-dada2 using the same pyro flag used in the CutPrimers-based workflow [11]. Because DADA2 models error individually per sequencing run while denoising, the DADA2 script was again run separately for each sequencing run. After denoising, run-specific feature tables and representative sequences were merged to generate one master feature table and representative sequence list for each V region.

#### Taxonomy classification

Representative sequence sets for each DADA2 ASV were then used for taxonomy classification using VSEARCH global sequence alignment (**Fig 2C**) [28]. VSEARCH was used over the Bayesian q2-feature-classifier, as the q2 feature classifier does not perform well with Ion Torrent sequences (N. Bokulich, personal communication, March 17, 2020). Three reference databases were used for taxonomic classification. Greengenes [version 13.8; 29] and Silva [version 138; 30] databases were imported from QIIME2 data resources, and the Ribosomal Database Project (RDP; version 16) was modified to QIIME2 expected format with custom scripts and was then imported into QIIME2 as an artifact [31]. See **S2–S37 Appendices** for genus-level taxonomy tables created using the CutPrimers- and Cutadapt-based pipelines outlined in **Fig 2**.

### Merging ASV tables across V regions, database and processing workflow at the genus level (CutPrimers-based pipeline)

Once a count ASV table was generated using the CutPrimers-based pipeline, ASVs were summarized at the taxonomic level of genus and any ASV that was assigned to the same genus was summed to facilitate cross-V region and database comparisons. If an ASV was assigned to a higher level than genus, then that ASV was renamed as “Assigned Higher”. The RA values at the genus level were calculated for each sample and tables were merged. If an ASV was assigned to *Escherichia*, then this ASV was renamed as *Escherichia/Shigella* for consistency due to anomalous assignment between these two genera; *Escherichia* is only classified to the family level in Greengenes [29], named as *Escherichia/Shigella* in RDP [31], and named as *Escherichia-Shigella* in Silva [30]. Also, since *Propionibacterium* has been renamed to *Cutibacterium* [32], then any ASV assigned to either one of these genera was renamed to *Cutibacterium*/*Propionibacterium*.

### Calculation and comparison of V-specific reads

Total reads from each V regions (and reads with no adapter) over all samples were averaged. V-specific reads were expressed as a mean across all samples and as a percentage of the total number of reads obtained per sample (prior to V-specific amplicon deconvolution). To determine if V region length affected the total number of V-specific reads the total amplicon length for each V region mapping to the *Escherichia coli* 16S rRNA gene [1] was compared against the average number of reads that mapped to same targeted V-region(s) using Spearman correlation coefficients.

### Global similarity and taxon-based accuracy metrics

Bray-Curtis dissimilarity, Jensen-Shannon divergence and Euclidean distance measures were used to evaluate agreement between the expected distribution and the observed distributions of the mock communities for all even and staggered samples (**see S1 Appendix for source code**) in Python using SciPy (version 1.8.0). The expected RA values at the genus level for each mock community is provided in **S1 Table**. Annotation accuracy (calculated as observed/expected (O/E) ratio) was determined for each genus using customized Python scripts (**see S1 Appendix for script**). The O/E ratio was calculated by dividing the observed RA (%) from the feature table by the expected RA (%) of that genus according to the specific mock community type (**S1 Table**). A value of 1 indicates perfect agreement between the expected and observed RA, a value less than 1 indicates the actual RA (%) was less than the expected RA for that mock bacterial community, and a value greater than 1 indicates the actual RA (%) was higher than expected the expected RA in the mock community for that individual taxon. For distance metrics and the O/E ratio for each taxon, values were summarized by V region (V2, V3, V4, V6-7, V8 and V9) and reference database (Silva, Greengenes, and RDP) for both pipelines (using CutPrimers or Cutadapt) in the evenly spaced and staggered mock bacterial communities.

#### Statistics

Statistical analyses were performed using the JMP™ Statistical Discovery Software version 15 (SAS Headquarters, Cary, NC). Differences in total reads extracted were compared across V regions and workflows (i.e. CutPrimers versus Cutadapt) using non-parametric testing (Wilcoxon Signed rank or Kruskal-Wallis tests). V-region specific amplicon length was compared to the number of V-specific reads amplified using Spearman correlation coefficient. Composition of mock bacterial community samples were evaluated by the Shannon alpha diversity index to measure the richness and evenness of individual bacterial communities. To evaluate the compositional difference between V regions and reference database for the CutPrimers-based pipeline, average RA values from the mock communities and the expected relative abundance for both the even and the staggered mock community samples were submitted to Principal Component Analysis in the JMP™ Statistical Discovery software. The first two principal components were plotted in bivariate plots. Differences in global distance metrics, O/E ratios and alpha diversity were calculated across reference databases and V regions for the CutPrimers-based pipeline using non-parametric testing (Wilcoxon Signed rank or Kruskal-Wallis tests). Post-hoc testing with the false discovery rate correction was applied when appropriate. Results are presented as mean ± standard deviation in tables and figures. Statistical significance was defined as *p* < .05.

## Results

The final data set used for the analysis in this work consisted of a total of 41 mock community samples from various labs sequenced using Ion Torrent sequencing platforms. All libraries were generated using the Ion 16S^TM^ Metagenomics multi-amplicon kit (**Table 1**).

### Average sequences per V region does not differ across amplicon deconvolution workflows

To compare the number of V-specific reads resulting from the CutPrimers and Cutadapt amplicon deconvolution workflows, reads mapping to the V region(s) targeted by the forward and reverse primers were averaged (**Table 2**). There was significant variability in the number of reads that mapped to specific V region(s) for both the CutPrimers (*p* < .0001) and Cutadapt (*p* < .0001) workflows. The average summed V-specific reads (summed across V2-V9 amplicons) were comparable between the CutPrimers and Cutadapt workflows (89.70 ± 5.86% versus 94.03 ± 1.87%, respectively; **Table 2**). The percentage of V-specific reads were not different for V2 (*p* = .28), V3 (*p* = .79), V4 (*p* = .72), V6-7 (*p* = .75), V8 (*p* = .65), or V9 (*p* = .76) between CutPrimers versus Cutadapt. For the Cutadapt workflow, a notable consideration was the need to include the “^” symbol at the beginning of the “—p-front” parameter (**Fig 2B**, **S1 Appendix)** otherwise there were large discrepancies between CutPrimers and Cutadapt and the summed total of V-specific reads greatly surpassed the expected total number of sequenced reads at 161.71 ± 7.70% (**S2 Table**).

**Table 2:** Average sequences per V region comparing CutPrimers versus Cutadapt.

V Region	Reads CutPrimers	Reads Cutadapt	Total Reads (Sample)	% Total Reads CutPrimers	% Total Reads Cutadapt
**2**	32,923.66 ± 21,356.37*	44,598.36 ± 11,615.13*	617,410.80 ± 452,297.61	5.98 ± 2.63	7.49 ± 4.85
**3**	180,767.93 ± 133,940.63*	181,773.90 ± 133,488.98*	617,410.80 ± 452,297.61	29.19 ± 5.56	29.60 ± 5.65
**4**	77,965.95 ± 56,527.49*	79,196.95 ± 55,721.23*	617,410.80 ± 452,297.61	12.80 ± 2.01	13.28 ± 2.11
**6–7**	141,118.29 ± 119,970.73*	144,510.41 ± 121,927.86*	617,410.80 ± 452,297.61	20.40 ± 5.83	21.11 ± 5.91
**8**	89,393.07 ± 63,675.28*	96,739.80 ± 71,689.65*	617,410.80 ± 452,297.61	14.85 ± 3.67	16.35 ± 6.15
**9**	41,814.22 ± 33,688.15*	43,038.34 ± 34,245.34*	617,410.80 ± 452,297.61	6.47 ± 2.26	6.73 ± 2.39
**No adapter**	53,427.68 ± 32897.05	N/A	-	-	-
**Summed V Total°**	563,983.12 ± 423,673.83	582,485.29 ± 431,025.69	-	89.70 ± 5.86%	94.03 ± 1.87%

V: Variable region. Reads are presented as the average absolute number of reads and the percentage of the total reads for each sample. Total reads are the sum of all sequences/reads for each mock community sample (same for each sample regardless of workflow). Cutadapt searches the entire demultiplexed sequence file for each primer (forward or reverse) and discards all other reads; therefore, “no adapter” row was unable to be calculated for Cutadapt. °Summed V total is the total number of reads for regions V2, V3, V4, V6-7, V8 and V9 after Cutadapt or cutPrimers, respectively (note- “no adapter” sequences not included as this is not calculated with the Cutadapt pipeline).

*p < .05 between V regions (holding cutPrimers vs Cutadapt workflow constant)

^ǂ^*p* < .05 between cutPrimers vs Cutadapt workflows (holding V region constant).

When holding workflow constant and comparing across V-specific reads, the V2 region had the smallest percentage of total reads for CutPrimers (5.98 ± 2.63), and was significantly lower compared to V3 (*p* < .001), V4 (*p* < .001), V6-7 (*p* < .001), and V8 (*p* < .001). Using Cutadapt, V9 had the lowest percentage of total reads (6.73 ± 2.39) and was significantly lower than V3 (*p* < .001), V4 (*p* = .001), V6-7 (*p* = .029), and V8 (*p* < .001). Like CutPrimers, the percentage of total reads that mapped to V2 using Cutadapt was also significantly lower compared to V3 (*p* < .001), V4 (*p* < .001), V6-7 (*p* < .001), and V8 (*p* < .001). V3 had the largest percentage of V-specific reads for both the CutPrimers (29.19 ± 5.56) and Cutadapt (29.60 ± 5.65) workflows, and was significantly higher compared to V2 (*p* < .001), V4 (*p* < .001), V8 (*p* = .001), and V9 (*p* < .001), and V2 (*p* < .001), V4 (*p* < .001), V8 (*p* = .001), and V9 (*p* < .001) for CutPrimers and Cutadapt, respectively. We subsequently investigated whether the hypothetical nucleotide length of a targeted V region(s) was associated with the total number of reads that mapped to that V region. The length of the V region targeted by the multi-amplicon primers was not associated with the number of reads that mapped to the respective V region in either workflow (CutPrimers: Spearman r = -0.13, *p* = .811; Cutadapt: Spearman r = -0.11, *p* = .831; **S1 Fig**).

### V regions amplified and reference database influence overall agreement with mock bacterial community samples

**Fig 3:**
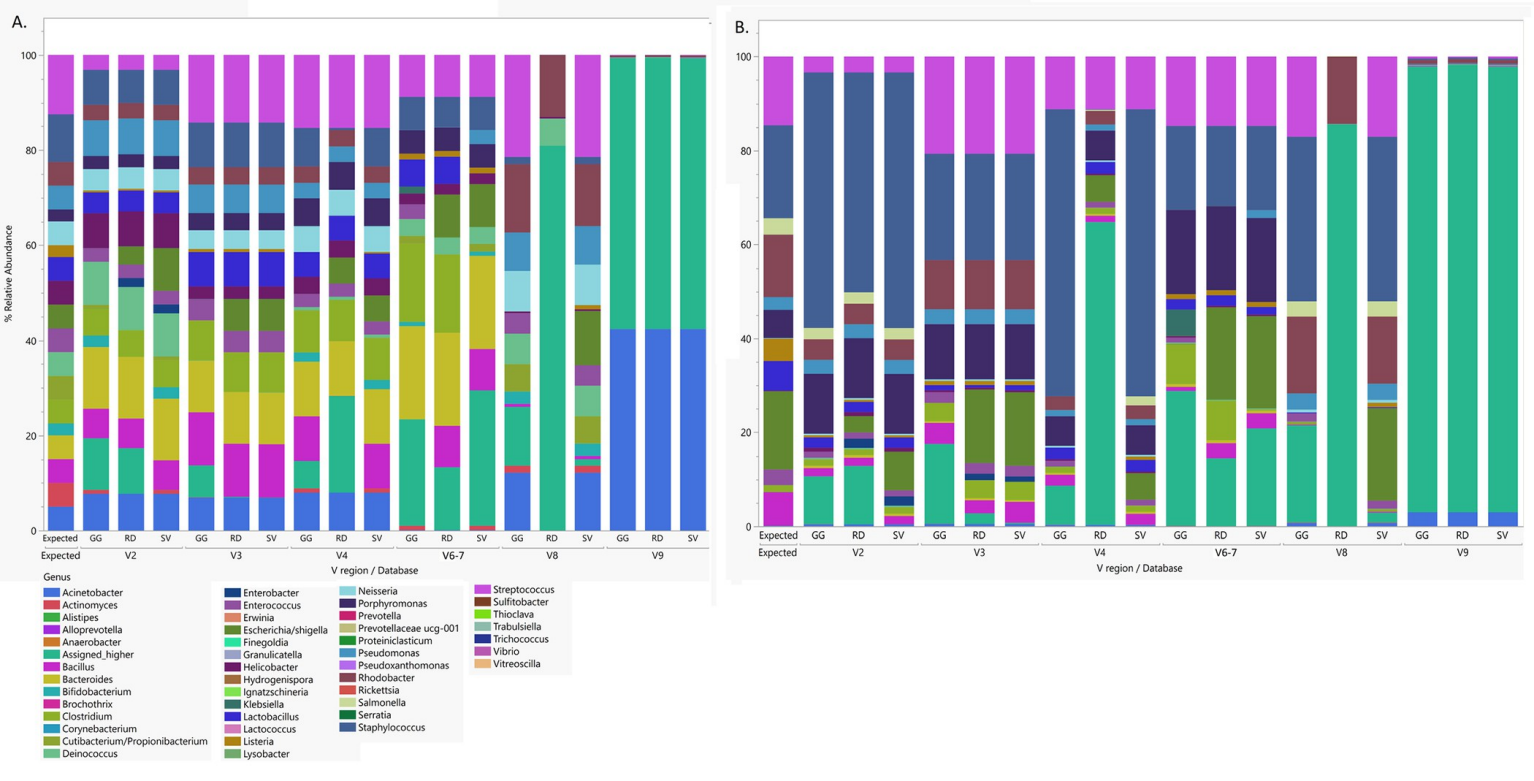
Averaged expected and actual mock community abundance by V region and reference database. **A.** Averaged evenly distributed mock community samples for the CutPrimers bioinformatics workflow stratified by V region and reference database. **B.** Averaged staggered mock community samples for the CutPrimers bioinformatics workflow stratified by V region and reference database. The expected mock community abundance (boxed) is the averaged RA for expected listed abundance on the package insert for evenly spaced and staggered mock bacterial communities.

The RA of mock bacterial community taxa that were annotated using the CutPrimers-based pipeline varied greatly across V regions and reference databases (**Fig 3**). Similar to other analysis pipelines, the CutPrimers-based pipeline produced ASVs that could not be assigned to the taxonomic level of genus, but the percentage of features that were “assigned higher” varied across V regions and reference databases (**Fig 3**, **S3 Table**). CutPrimers identified a total of 5 additional genera in the even mocks and 11 in the staggered mocks that were not present in the mock samples. The most notable unexpected bacteria (via a high RA or were annotated across multiple V regions) were *Alistipes*, *Enterobacter*, *Finegoldia*, *Klebsiella*, *Prevotella*, and *Rickettsia* (**S3 Table**). Nevertheless, across all V regions and reference databases, the summed RA of unexpected taxa at the level of genus were 0.34 ± 0.72% and 0.81 ± 1.53% for CutPrimers even and staggered samples, respectively. In feature tables created using the CutPrimers-based processing pipeline, the V9 region was the most notably different from the expected mock bacteria community in evenly spaced and staggered samples across all reference databases used for taxonomy assignment. Most of the taxa for region V9 were not annotated to the genus level and classified as “Assigned Higher” as the highest resolution many features were assigned were at the taxonomic level of family, order or phylum.

**Fig 4:**
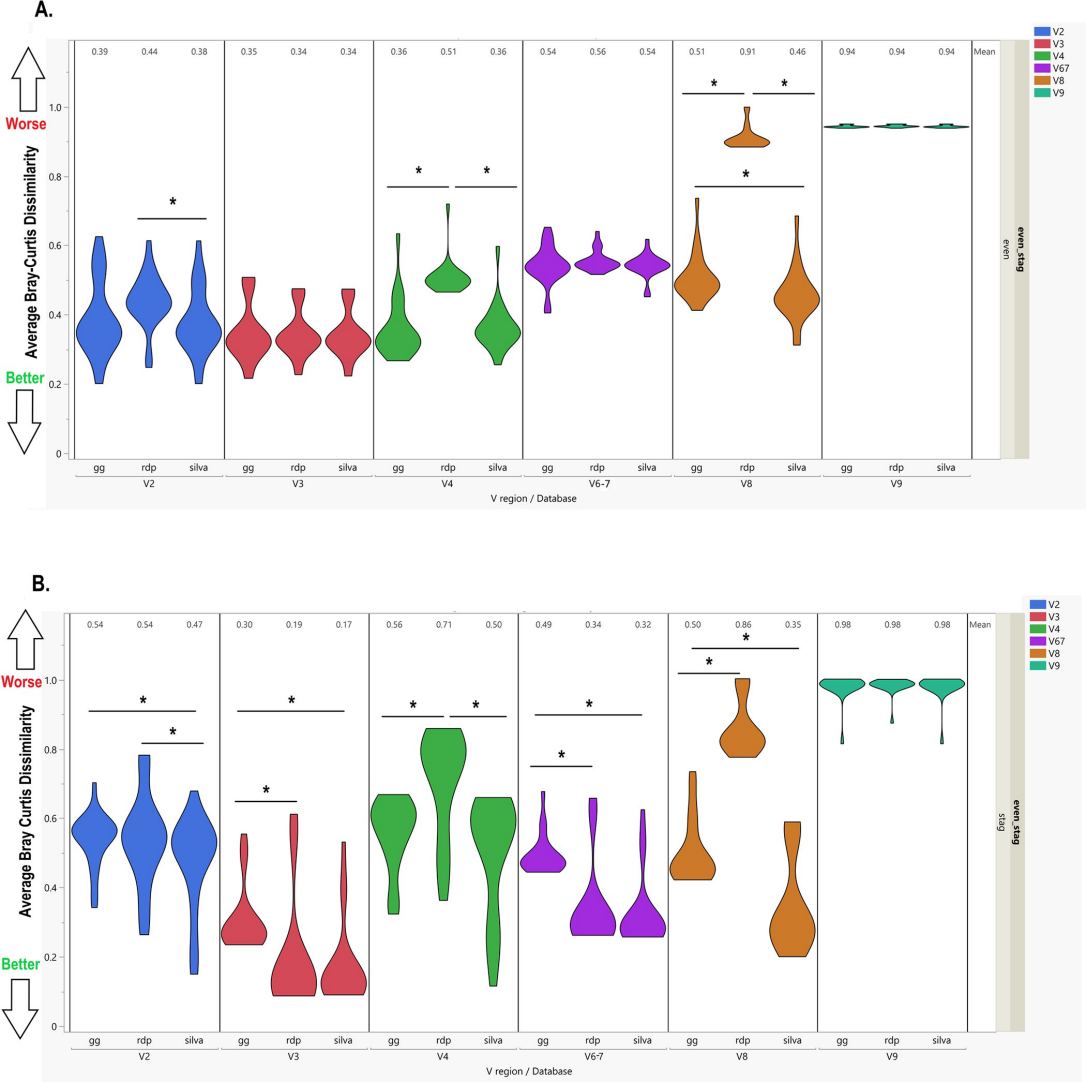
Overall mock community agreement with expected abundance using CutPrimers. **A.** Comparison of Bray-Curtis distance in the evenly spaced mock community samples using the CutPrimers-based pipeline. Scores closer to 0 indicate better agreement with expected abundance, while scores closer to 1 indicate worse agreement. Regions V3, V6-7 and V9 did not have significant differences in average Bray-Curtis distance across reference databases used for taxonomy classification. In the evenly spaced mock community samples, region V3 had low Bray-Curtis distances across all reference databases indicating feature tables from the V3 region had the closest agreement with expected mock community annotation and abundance. **B.** Comparison of Bray-Curtis distance in the staggered mock community samples using the CutPrimers-based pipeline. All regions with the exception of V9 had significant differences in average Bray-Curtis distance across reference databases used for taxonomy classification. In the staggered mock community samples, region V3 also had the lowest Bray-Curtis distances, although feature tables that used Greengenes for taxonomy classification had higher average distances, compared to Silva and RDP. Differences in Bray-Curtis distance across reference databases for each V region were tested using non-parametric testing Kruskal-Wallis tests. *p < .05.

Global distance metrics (Bray-Curtis, Jensen-Shannon, and Euclidean) were used to assess overall agreement of observed abundance values with expected abundance values. The V3 feature tables had the lowest Bray-Curtis distances (best agreement) in both evenly spaced (0.35 ± 0.08, 0.34 ± 0.07, 0.34 ± 0.07 for Greengenes, Silva, and RDP, respectively) and staggered (0.30 ± 0.09, 0.17 ± 0.12, 0.19 ± 0.17 for Greengenes, Silva, and RDP, respectively) mock bacterial community samples. Bray-Curtis distances did not differ by reference database in the even mock samples for V3 (*p* = .368; **Fig 4A**); however, in the staggered mock samples, Greengenes had significantly higher distances compared to RDP and Silva (**Fig 4B, S4 Table**). V3 feature tables also demonstrated the lowest average Euclidean distance and Jensen-Shannon divergence metrics across both even and staggered mock samples (**S2 Fig, S4 Table)**. In the staggered mock samples, more variance was present in Bray Curtis distances, and database-specific differences were present in all V regions except V9 (**Fig 4B**). Region V9 performed the worse on both the even and staggered samples with the highest distances for all three measures compared to the other V regions (**Figs 4** and **S2 and S4 Table**). Interestingly, there were not database-specific differences for any of the V9 global metrics using CutPrimers.

To visualize similarities and differences across V regions and reference databases, alpha and beta diversity metrics were calculated and visualized in the evenly spaced and staggered mock community samples. Shannon index did not significantly differ across reference databases in V2, V3 or V9 in the even mock community samples (**S3 Fig**). Shannon index was significantly lower in RDP in regions V4 and V8, while mock samples classified using Silva were significantly lower versus RDP and Greengenes in V6-7. Conversely, in the staggered mock community samples, alpha diversity only differed by reference database in region V8, where RDP was significantly lower versus Greengenes and Silva (**S3 Fig**). When samples were clustered on Principal Components Analysis plots (**S4 Fig**), mock samples classified using RDP were less similar versus mock samples classified using Silva or Greengenes, and this was most notable in the staggered mock community samples.

### Taxon-specific classification accuracy is dependent on V region amplified and reference database

**Fig 5:**
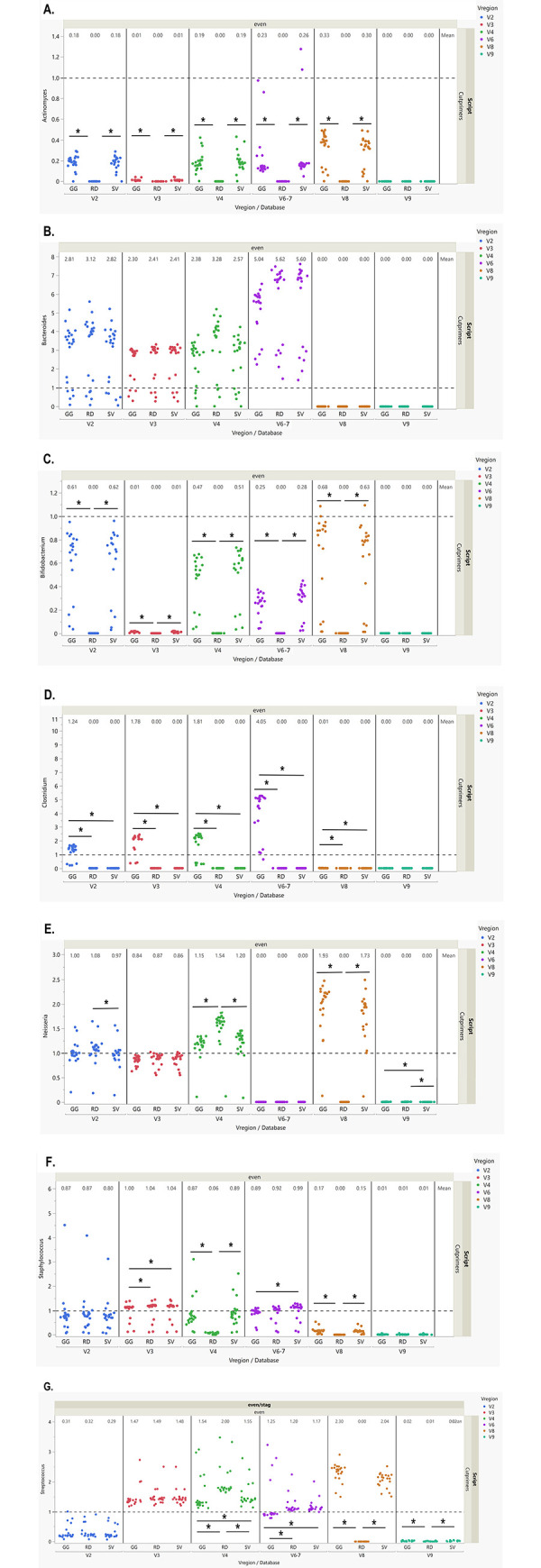
Observed/Expected ratio differences in evenly distributed mock bacterial community samples with CutPrimers workflow. **A.** Average O/E ratio of *Actinomyces* in evenly distributed mock community samples stratified by V region and reference database. All V-region specific feature tables, with the exception of V9, had significant differences across reference databases. **B.** Average O/E ratio of *Bacteroides* in evenly distributed mock community samples stratified by V region and reference database. There were no significant O/E ratio differences across reference databases in any of the V region-specific feature tables. **C.** Average O/E ratio of *Bifidobacterium* in evenly distributed mock community samples stratified by V region and reference database. All V-region specific feature tables, with the exception of V9, had significant differences across reference databases **D.** Average O/E ratio of *Clostridium* in evenly distributed mock community samples stratified by V region and reference database. All V-region specific feature tables, with the exception of V9, had significant differences across reference databases **E.** Average O/E ratio of *Neisseria* in evenly distributed mock community samples stratified by V region and reference database. The V2, V4, V6-7 and V9 feature tables had significant observed/expected ratio differences across reference databases. **F.** Average O/E ratio of *Staphylococcus* in evenly distributed mock community samples stratified by V region and reference database. The V3, V4, V6-7 and V9 feature tables had significant O/E ratio differences across reference databases. **G.** Average O/E ratio of *Streptococcus* in evenly distributed mock community samples stratified by V region and reference database. The V4, V6-7, V8, and V9 feature tables had significant O/E ratio differences across reference databases. Differences in O/E ratio values across reference databases for each V region were tested using non-parametric testing Kruskal-Wallis tests. *p < .05.

O/E ratios were calculated for each genus to assess genus level agreement with the expected RA using the CutPrimers-based pipeline. *Neisseria* had the best O/E ratio for the even mock samples at regions V2 and V3, and the ratios were consistent across reference databases (**Fig 5E, S5 Table**). Conversely, for *Staphylococcus* and *Streptococcus*, the V6-7 region had acc O/E ratios close to 1 for the even mock samples (**Fig 5F and 5G**). In the staggered mock samples, *Bacteroides*, *Neisseria*, *Streptococcus* and *Staphylococcus* (**S5 Fig**) had O/E ratios close to 1, but the V3 region had trouble annotating *Actinomyces* and *Bifidobacterium* with O/E ratios close to 0 (**S5 Fig**). This could be due to the sensitivity of the sequencing assay since both of these genera are represented at extremely low abundance of 0.02 or lower in the staggered mock samples. *Clostridium* was not annotated using the RDP or Silva reference databases for region V3, but Greengenes showed over-representation with ratios more than twice the expected (2.72 ± 0.71; **S5 Fig, S6 Table**). The V8 and V9 regions had O/E ratios close to 0 for most taxa in the evenly spaced and staggered mock bacterial community samples, with some exceptions (**Figs 5 and S5, S5 and S6 Tables**). See **S7–S12 Tables** for distance and O/E metrics, stratified by mock type.

## Discussion

In this manuscript we present two processing pipelines for microbiome data of mixed orientation, multi-V region amplicons from the Thermo Fisher Ion Metagenomics™ kit used on the Ion Torrent Sequencing platform. One pipeline (CutPrimers-based) presents a workflow for users of the kit with no knowledge about the primer sequences, while the other pipeline (Cutadapt-based) is used in cases when primer sequences are known. The two pipelines utilize the QIIME2 open-source platform for microbiome analysis, with some pre-processing steps outside of QIIME2 in the CutPrimers-based pipeline. Known mock communities were used in this work for benchmarking the accuracy of the two pipelines along with other specific variables such as V region V region and reference database. This work was developed in an effort to shed light on contributing biases that one might consider when designing future microbiome analysis pipelines. Previously, researchers using the Ion 16S Metagenomics™ multi-amplicon kit were unable to easily separate amplicons into specific V regions since the primer sequences used were proprietary and therefore traditional adapter removal workflows like Cutadapt or CutPrimers could not be applied. However, now, for the first time, we present the Metagenomics PP plugin, which allows researchers to separate amplicons into their respective V regions in order to facilitate typical downstream analyses such denoising and taxonomic classification can be employed.

As more bioinformatics tools for processing microbiome data become available, there is an increased need for researchers to be able to customize analysis pipelines to address the aims of the research study while controlling for variables of interest. While this work introduces the MetagenomicsPP plugin as a way to deconvolute multiple V region amplicons into their respective V regions, another aim was to illustrate a pre-processing workflow for Ion Torrent specific data which was not readily available as a tutorial in the QIIME2 environment. Although the overall analysis steps are similar to pipelines specific to Illumina data, there are some modifications that are made for optimal performance at the denoising step and taxonomy classification step. Namely, for Cutadapt (V-specific amplicon deconvolution) the -p front flag and “^” symbol prior to the primer sequence are necessary to prevent matching to nested primers and double counting amplicons matching to specific primers (see **S1 Appendix** for code syntax). Additionally, for denoising in DADA2, the “pyro” flag is employed and the accepted read length in our pipeline was shorter than it may be for other Ion Torrent-based pipelines due to the multi-amplicon sequencing data. Furthermore, for taxonomy classification, VSEARCH taxonomy alignment is preferable for Ion Torrent reads [28], as compared to the q2-feature-classifier plugin [33] due to the mixed orientation format that comes from Ion Torrent sequencing data.

Both the CutPrimers-based pipeline and Cutadapt-based pipeline have similar analysis steps (**Fig 2**), but a major difference between the two is where the adapter removal step occurs and whether the researchers knows the primer sequences or not. In the Cutadapt-based pipeline, the forward and reverse reads are run separately through Cutadapt and DADA2, since the forward and reverse V-specific primers generated unique sequence amplicons. In the CutPrimers-based pipeline, all V regions and runs were processed separately, however, since the Metagenomics PP plugin generates split V region specific reads combining both forward and reverse reads together, the combined V region subsets were run through DADA2 together. Nevertheless, the principle of running each sequencing run separately through DADA2 still remains, so sequencing error can be independently modeled for each run [11]. In both pipelines, the separate feature tables and sequences are merged after DADA2, to generate one combined feature table for taxonomy assignment and downstream analyses. The Cutadapt-based pipeline can also be used as a guide for single amplicon data from Ion Torrent reads, as the main requirement for this pipeline is that the sequences for the primers are known. Similarly, these microbiome analysis pipelines are also relevant for Illumina sequences, but researchers with sequences generated from Illumina platforms should also reference the freely available tutorials on the QIIME2 user guide aimed specifically for Illumina or paired-end sequences.

The total number of V region-specific reads generated from the CutPrimers and Cutadapt workflows were largely similar. An important caveat is that when the “^” symbol was not placed at the beginning of the primer sequence for the Cutadapt workflow (see **S1 Appendix** for code), which allowed matches only when the primer was at the beginning of a sequence, the percentage of summed V-specific reads largely outnumbered the total expected number of reads and total reads for CutPrimers at >150% (**S2 Table**). When Cutadapt was allowed to search the pool of demultiplexed amplicon sequences, a primer sequence could match the query even if associated with the preceding or subsequent V-specific amplicon (based on the primer location on the 16S gene) causing duplicate matches and the same amplicon to be assigned to multiple V regions. With the “^” symbol and Cutadapt only searching at the beginning of a read, there were not significant differences in the total number of reads summed across V regions or in any percentage of reads mapping to a specific V region. Nevertheless, holding workflow (Cutadapt or CutPrimers) constant, there were significant differences for the percentage of V-specific reads across V regions (V2-V9). V3 had the highest percentage of V-specific reads using both CutPrimers and Cutadapt workflows, and V3 had a significantly higher percentage of reads compared to V2, V4, V8 and V9. Additionally, the percentage of reads mapping to V2 was significantly lower compared to all other V regions, with the exception of V9 for both CutPrimers and Cutadapt both primer deconvolution workflows. Although these V-region specific differences in average reads were consistent across workflows, this may be a source of bias in taxonomic annotation of features across V regions, and should be considered when interpreting microbiome results.

We also demonstrate in the following analysis that taxonomic classification of multi-amplicon sequences generated from the Ion 16S™ kit varies based on V region of the 16S gene amplified and reference database employed, based on the CutPrimers workflow. As anticipated from our experience with previous analyses [34], the V9 region performed poorly across all reference databases for both global agreement with the mock bacterial community RA and with individual taxonomic accuracy statistics. Global distance metrics ranged from 0.69–0.94 in the even mock samples, and was even higher in the staggered mocks. To that note, we caution any researcher who uses the kit to consider excluding feature tables generated from that region in downstream analyses.

When global distances were considered, Bray-Curtis metrics ranged between 0.32 to 0.51 from regions V2-4 in the even mock samples, and were 0.17 to 0.71 in the staggered based communities. The significant differences observed between databases (with the exception of the V3 region) was likely due to RDP exhibiting higher dissimilarity metrics in the CutPrimers-based pipeline. This was almost consistently observed, indicating that it should not be a preferred reference database for alignment in microbiome studies using that pipeline if targeting regions V 2–4. However, if RDP is the reference database of choice, the V3 region may be the preferred region to target, since this analysis indicated better agreement with the expected RA and similar metrics to other reference databases in this V region. The V2, V3, V6-7, and V8 regions had significant Bray-Curtis differences in the staggered mock samples, where Greengenes had less agreement with the expected mock RA in all cases. As staggered mock community samples are more representative of a typical distribution of gut microbiome-associated bacteria, Silva may be a preferred reference database, possibly due to the fact it has been more recently updated and is a larger database encompassing approximately 6,300 genera versus approximately 2,000 in the Greengenes reference database [24,25]. Further investigation and benchmarking of this multi-amplicon data will be needed to confirm these findings.

The variance in individual taxa accuracy metrics demonstrated that bacterial taxonomic classification agreement with expected mock RA varied by V region and occasionally reference database. *Actinomyces* and *Clostridium* were largely underrepresented in the even mock samples with the exception of *Clostridium*, demonstrating a more accurate RA representation in the V2-V4 regions when the Greengenes reference database was used. Conversely, *Bacteroides*, for example, had RA values in the feature table that were larger than was expected in regions V2-V6-7, but had mean accuracy values close to zero in regions V8-9 across all reference databases in both the even and staggered mocks. As specific bacterial taxa are more predominant in different microbiome habitats, such as the oral and gut microbiome [35], researchers should consider the research microbiome site sampled in addition to other factors if one individual V region is emphasized for downstream analysis. The accuracy measure (O/E ratio) used in this work to reports how close the observed genus relative abundance value was to the expected value; however, we are unable to report how well these ratios compare to other literature since there is a scarcity of benchmarking literature targeting the multi-amplicon kits currently available. Importantly, future benchmarking work using multi-amplicon kits and Ion Torrent sequences is needed to confirm our results. Furthermore, this variability in taxonomic annotation across V regions supports our view that it is imperative to plan the sequencing strategy in line with the research questions, the dominant taxa in the microbiome being targeted, and the available resources [4]. When V-region informed planning is not possible due to lack of previous data on the taxa of bacteria expected or a need for taxonomic resolution at the level of species or strain, full length 16S rRNA gene sequencing with long-read sequencing technology or shotgun metagenomics sequencing may be desirable [10]. However, when this is not feasible, using the benchmarking-associated resources presented in this manuscript to target two or more V regions in a bias-informed manner would be a valuable alternative in order to merge taxonomy tables and manage this variation in annotation efficiency.

Although we believe this manuscript and the pre-processing pipelines presented make a valuable contribution to the literature, there are some limitations in this research that we want to address. First, we present two pre-processing pipelines aimed at amplicon deconvolution and ASV generation, up to the point of feature or taxonomy table generation. Feature table combination strategies with multi-amplicon kits are outside the scope of this manuscript, but many strategies have been employed to synthesize additive information from multiple feature tables in downstream microbiome analysis [20,36]. Additionally, the benchmarking results presenting in this manuscript are only comparable to reads generated from the Ion 16S kit and Ion Torrent sequencing data. This work focused on a finite number of mock samples and as such, discrepancies in taxa names were accounted for manually. For future work employing a larger number of samples or taxa, we suggest using the NCBI taxonomy IDs for taxa merging across many tables. Nevertheless, we believe this workflow and pre-processing pipeline will be useful for analyzing data in the QIIME2 environment and will allow for flexibility in pre-processing and downstream analysis of microbiome sequencing data.

## Conclusions

We present a specialized script to allow microbiome researchers to separate multi-amplicon data from the Ion16S Metagenomics™ Kit into V specific reads, and outline two microbiome analysis pre-processing pipelines based on CutPrimers and Cutadapt workflows for amplicon deconvolution. Although the two workflows had some differences in primer targeting and removal, along with differences in the number average amplicons generated across V regions, overall summed reads across V regions were largely similar. Nevertheless, for the CutPrimers-based pipeline, both global and taxon-specific metrics differed significantly across V regions and reference databases used for taxonomy classification. There was notable variance with respect to V region and reference database considered across bacterial taxa with some O/E ratios being close one and others being close to 0). In conclusion, several factors need to be considered when performing microbiome analysis. The V region and reference database used to create the taxonomy table both influenced overall agreement with the mock bacterial samples, and benchmarking data should be referenced when planning a microbiome study to consider these biases related to sequencing and data analysis.

## Supporting information

S1 Fig Bivariate plot of V region specific average number of reads compared to hypothetical V region length. The length of each V region-specific amplicon mapping to the Escherichia coli 16S rRNA gene that was targeted by each group of forward and reverse primers in the Ion 16STM Metagenomics Kit was calculated for each V region(s). Spearman rank correlation was computed to assess the relationship between amplicon length (x-axis) and the average number of reads (y-axis) that mapped to targeted V-region(s). Amplicon length was not associated with the total number of reads that mapped to a targeted V-region A. CutPrimers (r = -0.13, *p* = .811) or B. Cutadapt (r = -0.11, *p* = .831).
(PNG)

S2 Fig Distance metrics between expected versus observed abundances using CutPrimers-Based Pipeline for all V regions and databases. A. Average Euclidean distance from the evenly distributed mock community feature table to expected bacterial abundance stratified by V region and reference database. B. Average Jensen-Shannon distance from the evenly distributed mock community feature table to expected bacterial abundance stratified by V region and reference database. C. Average Euclidean distance from the staggered mock community feature table to expected bacterial abundance stratified by V region and reference database. D. Average Jensen-Shannon distance from the staggered mock community feature table to expected bacterial abundance stratified by V region and reference database. *p < .05 between reference database, holding V region constant.
(PNG)

S3 Fig Shannon index across V regions and reference databases in mock community samples analyzed with the CutPrimers-based pipeline. A. Comparison of alpha diversity using Shannon index in the evenly spaced mock community samples using the CutPrimers-based pipeline. B. Comparison of alpha diversity using Shannon index in the staggered mock community samples using the CutPrimers-based pipeline. *p < .05 between reference database, holding V region constant.
(PNG)

S4 Fig Overall community structure similarities and differences in mock samples analyzed with the CutPrimers-based pipeline. A. Principal Components Analysis plot of all evenly spaced mock community samples reduced to one point per reference database and V region using the CutPrimers-based pipeline. A. Principal Components Analysis plot of all staggered mock community samples reduced to one point per reference database and V region using the CutPrimers-based pipeline.
(PNG)

S5 Fig Observed/Expected ratio differences in staggered mock bacterial community samples with CutPrimers workflow. A. Average O/E ratio of Actinomyces in staggered mock community samples stratified by V region and reference database. V67 and V8 were the only feature tables that had significant O/E ratios that were significantly different across reference databases. B. Average O/E ratio of Bacteroides in staggered mock community samples stratified by V region and reference database. The V4 and V67 feature tables had significantly different O/E ratios across reference databases. C. Average O/E ratio of Bifidobacterium in staggered mock community samples stratified by V region and reference database. The V2, V67 and V8 feature tables had significant O/E ratio differences across reference databases. D. Average O/E ratio of Clostridium in staggered mock community samples stratified by V region and reference database. All V-specific feature tables, with the exception of V9, had significantly different O/E ratios across reference databases. E. Average O/E ratio of Neisseria in staggered mock community samples stratified by V region and reference database. The V3, V4, and V8 feature tables had significant O/E ratio differences across reference databases. F. Average O/E ratio of Staphylococcus in staggered mock community samples stratified by V region and reference database. The V3, V4, V67 and V8 feature tables had significant O/E ratio differences across reference databases. G. Average O/E ratio of Streptococcus in staggered mock community samples stratified by V region and reference database. The V3, V4, V67 and V8 feature tables had significant O/E ratio differences across reference databases.
(PNG)

S1 Table Expected relative abundances by mock bacterial community type. Genomic DNA from American Type Culture Collection (ATCC, Manassas, VA) for even (ATCC® MSA-1002) and staggered (ATCC® MSA-1003) mock microbiome standard samples were used for analysis. The mock communities contain 20 common bacterial species [14,15], that include both gram positive and gram-negative bacteria. Additional mock community sample sequencing information and expected abundances were compiled using the product information generously provided the external labs. Evenly distributed ATCC and BEI mock bacterial community samples, and staggered ATCC, BEI and Zymo mock samples were added to the even/staggered ATCC mock microbiome standard samples from the external institutions (Table 1).
(DOCX)

S2 Table Average sequences per V region comparing CutPrimers versus Cutadapt (Forward Primer Amplicons Only and Forward + Reverse Primer Amplicons). V: Variable region. Reads are presented as the average absolute number of reads and the percentage of the total reads for each sample. Total reads are the sum of all sequences/reads for each mock community sample (same for each sample regardless of workflow). Cutadapt searches the entire demultiplexed sequence file for each primer (forward or reverse) and discards all other reads; therefore, “no adapter” row was unable to be calculated for Cutadapt. °Summed V total is the total number of reads for regions V2, V3, V4, V67, V8 and V9 after Cutadapt or cutPrimers, respectively (note- “no adapter” sequences not included as this is not calculated with the Cutadapt pipeline). ¥ Cutadapt workflow performed without the “^” symbol preceding the primer sequence at the–p front flag (which only matches primers if they occur at the beginning of a read). *p < .05 between V regions (holding cutPrimers vs Cutadapt workflow constant); ^ǂ^*p* < .05 between cutPrimers vs Cutadapt workflows (holding V region constant).
(DOCX)

S3 Table Bacteria annotated in the mock community feature table that were unexpected or not classified to the taxonomic level of genus. The average relative abundance of bacteria at the taxonomic level of genus that were not present in the mock bacterial communities, stratified by V region and reference database for both CutPrimers and Cutadapt-based pipelines. *Assigned higher is any feature that was annotated at a taxonomic level higher than genus (i.e. Family, Class etc.). If cell is blank that indicates this taxon was not annotated in that V region and reference database.
(DOCX)

S4 Table Global distance metrics using CutPrimers. Even mock samples n = 21 (atcc_even n = 18; bei_even n = 3). Staggered mock samples n = 20 (atcc_stag n = 14; bei_stag n = 4; zymo_stag n = 2). Non-parametric tests were run to determine distance metric differences between V region (Kruskal-Wallis), reference databases (Kruskal-Wallis), and bioinformatics workflows (Wilcoxon Rank Sum), respectively. *p < .05 between V regions (holding reference database and workflow constant); Φ*p* < .05 between reference databases (holding V region and workflow constant); *p* < .05 between cutPrimers vs Cutadapt workflows (holding V region and reference database constant). Euclidean, Jensen-Shannon, and Bray-Curtis Dissimilarity scores range between 0–1 where a score of 0 indicates zero dissimilarity between expected and actual mock bacterial abundance (or that expected and actual abundance are identical) and a score of 1 indicates complete dissimilarity between actual and expected abundances.
(DOCX)

S5 Table Taxon-specific metrics for all evenly-spaced mock bacterial communities evenly-spaced mock bacterial communities V2, V3, V4, V6-7, V8, V9. Even mock samples n = 21 (atcc_even n = 18 samples; bei_even n = 3 samples). n/a = Bacteria listed was not in the specified mock community. Values (mean or standard deviation) were rounded to two decimal places, and values < 0.005 were rounded to 0.0 (not true zero in every case). Taxon-specific agreement was defined as the observed/expected ratio and calculated as the observed relative abundance (%) / expected relative abundance (%) for each genus. A value of 1 indicates perfect agreement, a value under 0–0.999 indicates the actual relative abundance (%) is less than expected, and a value over 1 indicates the actual relative abundance (%) is higher than expected in the mock community for that individual taxon. Non-parametric tests were run to determine precision metric differences between V region (Kruskal-Wallis), reference databases (Kruskal-Wallis), and bioinformatics workflows (Wilcoxon Rank Sum), respectively, for each individual genus. *p < .05 between V regions (holding reference database and workflow constant).
(DOCX)

S6 Table Accuracy metrics for all staggered mock bacterial communities: Staggered mock communities V2, V3, V4, V6-7, V8, V9. Staggered mock samples n = 20 (atcc_stag n = 14 samples; bei_stag n = 4 samples; zymo_stag n = 2 samples). n/a = Bacteria listed was not in the specified mock community. Values (mean or standard deviation) were rounded to two decimal places, and values < 0.005 were rounded to 0.0 (not true zero in every case). Taxon-specific agreement was defined as the observed/expected ratio and calculated as the observed relative abundance (%) / expected relative abundance (%) for each genus. A value of 1 indicates perfect agreement, a value under 0–0.999 indicates the actual relative abundance (%) is less than expected, and a value over 1 indicates the actual relative abundance (%) is higher than expected in the mock community for that individual taxon. Non-parametric tests were run to determine precision metric differences between V region (Kruskal-Wallis), reference databases (Kruskal-Wallis), and bioinformatics workflows (Wilcoxon Rank Sum), respectively, for each individual genus. *p < .05 between V regions (holding reference database and workflow constant); Φ*p* < .05 between reference databases (holding V region and workflow constant).
(DOCX)

S7 Table Global distance metrics by mock type. Even mock samples n = 21 (atcc_even n = 18 samples; bei_even n = 3 samples). Staggered mock samples n = 20 (atcc_stag n = 14 samples; bei_stag n = 4 samples; zymo_stag n = 2 samples). Euclidean, Jensen-Shannon, and Bray-Curtis Dissimilarity scores range between 0–1 where a score of 0 indicates zero dissimilarity between expected and actual mock bacterial abundance (or that expected and actual abundance are identical) and a score of 1 indicates complete dissimilarity between actual and expected abundances.
(DOCX)

S8 Table Taxon-specific metrics by mock type: Evenly-spaced ATCC mock bacterial community samples V2, V3, V4, V6-7, V8, V9. Even mock samples atcc_even n = 18. n/a = Bacteria listed was not in the specified mock community. Values (mean or standard deviation) were rounded to two decimal places, and values < 0.005 were rounded to 0.0 (not true zero in every case). Taxon-specific agreement was defined as the observed/expected ratio and calculated as the observed relative abundance (%) / expected relative abundance (%) for each genus. A value of 1 indicates perfect agreement, a value under 0–0.999 indicates the actual relative abundance (%) is less than expected, and a value over 1 indicates the actual relative abundance (%) is higher than expected in the mock community for that individual taxon. Non-parametric tests were run to determine precision metric differences between V region (Kruskal-Wallis), reference databases (Kruskal-Wallis), and bioinformatics workflows (Wilcoxon Rank Sum), respectively, for each individual genus.
(DOCX)

S9 Table Taxon-specific metrics by mock type: Evenly-spaced BEI mock bacterial community samples V2, V3, V4, V6-7, V8, V9. Even mock samples bei_even n = 3. n/a = Bacteria listed was not in the specified mock community. Values (mean or standard deviation) were rounded to two decimal places, and values < 0.005 were rounded to 0.0 (not true zero in every case). Taxon-specific agreement was defined as the observed/expected ratio and calculated as the observed relative abundance (%) / expected relative abundance (%) for each genus. A value of 1 indicates perfect agreement, a value under 0–0.999 indicates the actual relative abundance (%) is less than expected, and a value over 1 indicates the actual relative abundance (%) is higher than expected in the mock community for that individual taxon. Non-parametric tests were run to determine precision metric differences between V region (Kruskal-Wallis), reference databases (Kruskal-Wallis), and bioinformatics workflows (Wilcoxon Rank Sum), respectively, for each individual genus.
(DOCX)

S10 Table Taxon-specific metrics by mock type: Staggered ATCC mock bacterial community samples V2, V3, V4, V6-7, V8, V9. Staggered mock samples atcc_stag n = 14 samples. n/a = Bacteria listed was not in the specified mock community. Values (mean or standard deviation) were rounded to two decimal places, and values < 0.005 were rounded to 0.0 (not true zero in every case). Taxon-specific agreement was defined as the observed/expected ratio and calculated as the observed relative abundance (%) / expected relative abundance (%) for each genus. A value of 1 indicates perfect agreement, a value under 0–0.999 indicates the actual relative abundance (%) is less than expected, and a value over 1 indicates the actual relative abundance (%) is higher than expected in the mock community for that individual taxon. Non-parametric tests were run to determine precision metric differences between V region (Kruskal-Wallis), reference databases (Kruskal-Wallis), and bioinformatics workflows (Wilcoxon Rank Sum), respectively, for each individual genus.
(DOCX)

S11 Table Taxon-specific metrics by mock type: Staggered BEI mock bacterial community samples V2, V3, V4, V6-7, V8, V9. Staggered mock samples bei_stag n = 4 samples. n/a = Bacteria listed was not in the specified mock community. Values (mean or standard deviation) were rounded to two decimal places, and values < 0.005 were rounded to 0.0 (not true zero in every case). Taxon-specific agreement was defined as the observed/expected ratio and calculated as the observed relative abundance (%) / expected relative abundance (%) for each genus. A value of 1 indicates perfect agreement, a value under 0–0.999 indicates the actual relative abundance (%) is less than expected, and a value over 1 indicates the actual relative abundance (%) is higher than expected in the mock community for that individual taxon. Non-parametric tests were run to determine precision metric differences between V region (Kruskal-Wallis), reference databases (Kruskal-Wallis), and bioinformatics workflows (Wilcoxon Rank Sum), respectively, for each individual genus.
(DOCX)

S12 Table Taxon-specific metrics by mock type: Staggered ZYMO mock bacterial community samples V2, V3, V4, V6-7, V8, V9. Staggered mock samples zymo_stag n = 2. n/a = Bacteria listed was not in the specified mock community. Values (mean or standard deviation) were rounded to two decimal places, and values < 0.005 were rounded to 0.0 (not true zero in every case). Taxon-specific agreement was defined as the observed/expected ratio and calculated as the observed relative abundance (%) / expected relative abundance (%) for each genus. A value of 1 indicates perfect agreement, a value under 0–0.999 indicates the actual relative abundance (%) is less than expected, and a value over 1 indicates the actual relative abundance (%) is higher than expected in the mock community for that individual taxon. Non-parametric tests were run to determine precision metric differences between V region (Kruskal-Wallis), reference databases (Kruskal-Wallis), and bioinformatics workflows (Wilcoxon Rank Sum), respectively, for each individual genus.
(DOCX)

S1 Appendix MetagenomicsPP plugin information and example scripts associated with the CutPrimers- and Cutadapt-based pipelines. (IPYNB)

S2 Appendix V2 GG CutPrimers Genus Taxonomy Table (Counts). (CSV)

S3 Appendix V2 RDP CutPrimers Genus Taxonomy Table (Counts). (CSV)

S4 Appendix V2 Silva CutPrimers Genus Taxonomy Table (Counts). (CSV)

S5 Appendix V3 GG CutPrimers Genus Taxonomy Table (Counts). (CSV)

S6 Appendix V3 RDP CutPrimers Genus Taxonomy Table (Counts). (CSV)

S7 Appendix V3 Silva CutPrimers Genus Taxonomy Table (Counts). (CSV)

S8 Appendix V4 GG CutPrimers Genus Taxonomy Table (Counts). (CSV)

S9 Appendix V4 RDP CutPrimers Genus Taxonomy Table (Counts). (CSV)

S10 Appendix V4 Silva CutPrimers Genus Taxonomy Table (Counts). (CSV)

S11 Appendix V6-7 GG CutPrimers Genus Taxonomy Table (Counts). (CSV)

S12 Appendix V6-7 RDP CutPrimers Genus Taxonomy Table (Counts). (CSV)

S13 Appendix V6-7 Silva CutPrimers Genus Taxonomy Table (Counts). (CSV)

S14 Appendix V8 GG CutPrimers Genus Taxonomy Table (Counts). (CSV)

S15 Appendix V8 RDP CutPrimers Genus Taxonomy Table (Counts). (CSV)

S16 Appendix V8 Silva CutPrimers Genus Taxonomy Table (Counts). (CSV)

S17 Appendix V9 GG CutPrimers Genus Taxonomy Table (Counts). (CSV)

S18 Appendix V9 RDP CutPrimers Genus Taxonomy Table (Counts). (CSV)

S19 Appendix V9 Silva CutPrimers Genus Taxonomy Table (Counts). (CSV)

S20 Appendix V2 GG Cutadapt Genus Taxonomy Table (Counts). (CSV)

S21 Appendix V2 RDP Cutadapt Genus Taxonomy Table (Counts). (CSV)

S22 Appendix V2 Silva Cutadapt Genus Taxonomy Table (Counts). (CSV)

S23 Appendix V3 GG Cutadapt Genus Taxonomy Table (Counts). (CSV)

S24 Appendix V3 RDP Cutadapt Genus Taxonomy Table (Counts). (CSV)

S25 Appendix V3 Silva Cutadapt Genus Taxonomy Table (Counts). (CSV)

S26 Appendix V4 GG Cutadapt Genus Taxonomy Table (Counts). (CSV)

S27 Appendix V4 RDP Cutadapt Genus Taxonomy Table (Counts). (CSV)

S28 Appendix V4 Silva Cutadapt Genus Taxonomy Table (Counts). (CSV)

S29 Appendix V6-7 GG Cutadapt Genus Taxonomy Table (Counts). (CSV)

S30 Appendix V6-7 RDP Cutadapt Genus Taxonomy Table (Counts). (CSV)

S31 Appendix V6-7 Silva Cutadapt Genus Taxonomy Table (Counts). (CSV)

S32 Appendix V8 GG Cutadapt Genus Taxonomy Table (Counts). (CSV)

S33 Appendix V8 RDP Cutadapt Genus Taxonomy Table (Counts). (CSV)

S34 Appendix V8 Silva Cutadapt Genus Taxonomy Table (Counts). (CSV)

S35 Appendix V9 GG Cutadapt Genus Taxonomy Table (Counts). (CSV)

S36 Appendix V9 RDP Cutadapt Genus Taxonomy Table (Counts). (CSV)

S37 Appendix V9 Silva Cutadapt Genus Taxonomy Table (Counts). (CSV)

## Abbreviations

DNA: Deoxyribonucleic Acid
MetagenomicsPP: Metagenomics Post Processor Plugin
O/E: Observed/Expected
RA: Relative Abundance
RDP: Ribosomal Database Project
rRNA: Ribosomal Ribonucleic Acid
V: Hypervariable (region of the 16S rRNA gene)
PCR: Polymerase Chain Reaction
