# Supplementary figures and images for: Multi-amplicon microbiome data analysis pipelines for mixed orientation sequences using QIIME2: Assessing reference database, variable region and pre-processing bias in classification of mock bacterial community samples
Source: PLoS One. 2023 Jan 13;18(1):e0280293. doi: 10.1371/journal.pone.0280293 (PMC9838852; doi:10.1371/journal.pone.0280293)

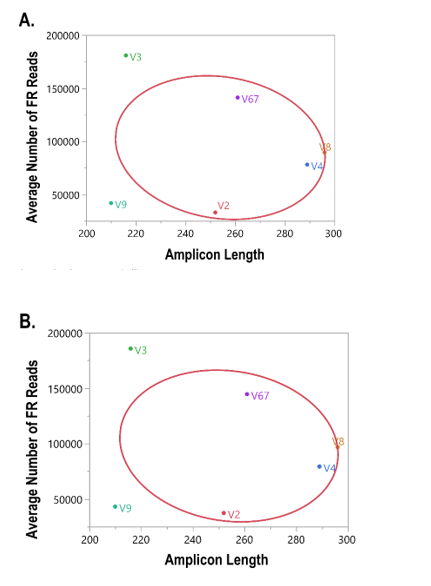

Supplement: S1 Fig — The length of each V region-specific amplicon mapping to the Escherichia coli 16S rRNA gene that was targeted by each group of forward and reverse primers in the Ion 16STM Metagenomics Kit was calculated for each V region(s). Spearman rank correlation was computed to assess the relationship between amplicon length (x-axis) and the average number of reads (y-axis) that mapped to targeted V-region(s). Amplicon length was not associated with the total number of reads that mapped to a targeted V-region A. CutPrimers (r = -0.13, p = .811) or B. Cutadapt (r = -0.11, p = .831). (PNG) [file pone.0280293.s001.png]

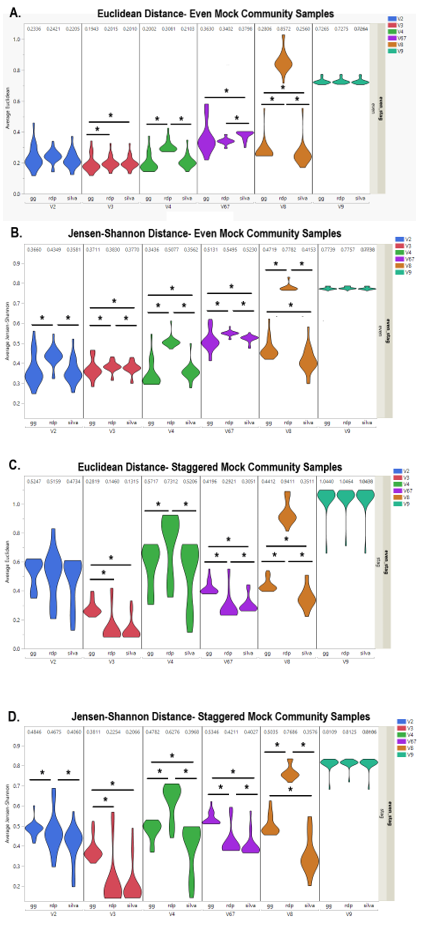

Supplement: S2 Fig — A. Average Euclidean distance from the evenly distributed mock community feature table to expected bacterial abundance stratified by V region and reference database. B. Average Jensen-Shannon distance from the evenly distributed mock community feature table to expected bacterial abundance stratified by V region and reference database. C. Average Euclidean distance from the staggered mock community feature table to expected bacterial abundance stratified by V region and reference database. D. Average Jensen-Shannon distance from the staggered mock community feature table to expected bacterial abundance stratified by V region and reference database. *p < .05 between reference database, holding V region constant. (PNG) [file pone.0280293.s002.png]

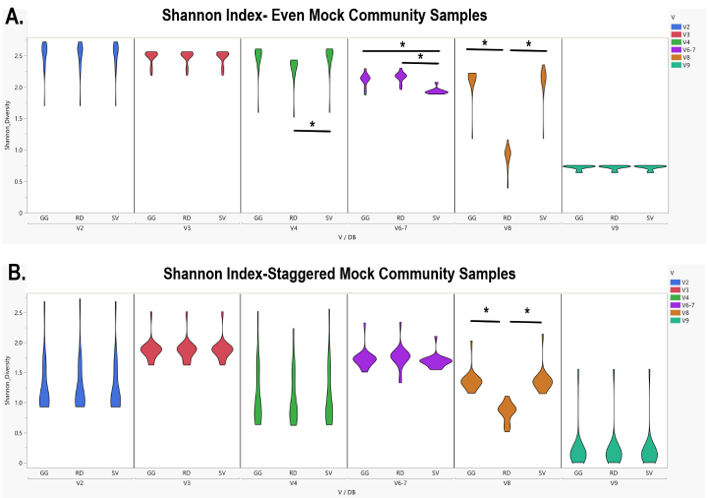

Supplement: S3 Fig — A. Comparison of alpha diversity using Shannon index in the evenly spaced mock community samples using the CutPrimers-based pipeline. B. Comparison of alpha diversity using Shannon index in the staggered mock community samples using the CutPrimers-based pipeline. *p < .05 between reference database, holding V region constant. (PNG) [file pone.0280293.s003.png]

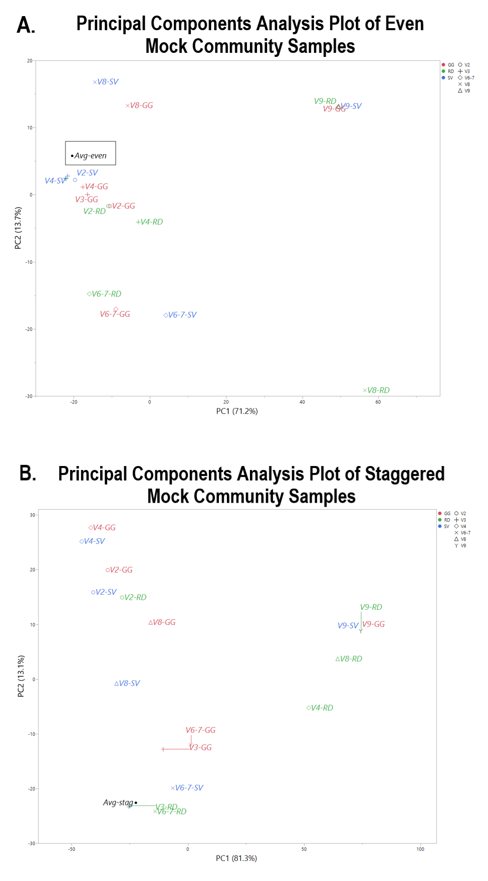

Supplement: S4 Fig — A. Principal Components Analysis plot of all evenly spaced mock community samples reduced to one point per reference database and V region using the CutPrimers-based pipeline. A. Principal Components Analysis plot of all staggered mock community samples reduced to one point per reference database and V region using the CutPrimers-based pipeline. (PNG) [file pone.0280293.s004.png]

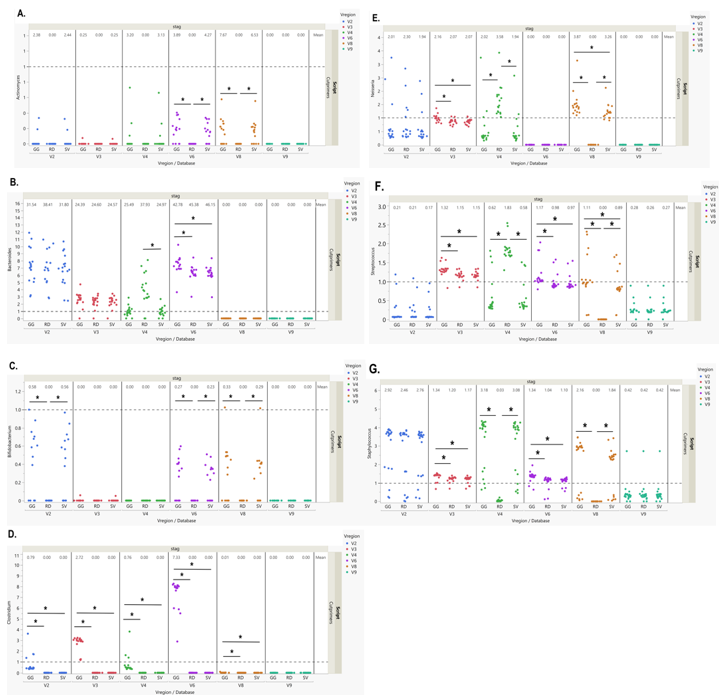

Supplement: S5 Fig — A. Average O/E ratio of Actinomyces in staggered mock community samples stratified by V region and reference database. V67 and V8 were the only feature tables that had significant O/E ratios that were significantly different across reference databases. B. Average O/E ratio of Bacteroides in staggered mock community samples stratified by V region and reference database. The V4 and V67 feature tables had significantly different O/E ratios across reference databases. C. Average O/E ratio of Bifidobacterium in staggered mock community samples stratified by V region and reference database. The V2, V67 and V8 feature tables had significant O/E ratio differences across reference databases. D. Average O/E ratio of Clostridium in staggered mock community samples stratified by V region and reference database. All V-specific feature tables, with the exception of V9, had significantly different O/E ratios across reference databases. E. Average O/E ratio of Neisseria in staggered mock community samples stratified by V region and reference database. The V3, V4, and V8 feature tables had significant O/E ratio differences across reference databases. F. Average O/E ratio of Staphylococcus in staggered mock community samples stratified by V region and reference database. The V3, V4, V67 and V8 feature tables had significant O/E ratio differences across reference databases. G. Average O/E ratio of Streptococcus in staggered mock community samples stratified by V region and reference database. The V3, V4, V67 and V8 feature tables had significant O/E ratio differences across reference databases. (PNG) [file pone.0280293.s005.png]
