# Supplementary material for: Multi-amplicon microbiome data analysis pipelines for mixed orientation sequences using QIIME2: Assessing reference database, variable region and pre-processing bias in classification of mock bacterial community samples
Source: PLoS One. 2023 Jan 13;18(1):e0280293. doi: 10.1371/journal.pone.0280293 (PMC9838852; doi:10.1371/journal.pone.0280293)
Supplement: S1 Table — Genomic DNA from American Type Culture Collection (ATCC, Manassas, VA) for even (ATCC® MSA-1002) and staggered (ATCC® MSA-1003) mock microbiome standard samples were used for analysis. The mock communities contain 20 common bacterial species [14,15], that include both gram positive and gram-negative bacteria. Additional mock community sample sequencing information and expected abundances were compiled using the product information generously provided the external labs. Evenly distributed ATCC and BEI mock bacterial community samples, and staggered ATCC, BEI and Zymo mock samples were added to the even/staggered ATCC mock microbiome standard samples from the external institutions (Table 1). (DOCX) [file pone.0280293.s006.docx]

**Supplemental Table 1. Expected Relative Abundances by Mock Bacterial Community Type**

| **Genus** | **Expected Abundance in Mock Community (%) by Mock Type** | | | | | | |
| --- | --- | --- | --- | --- | --- | --- | --- |
|  | **ATCC Even** | **BEI Even** | **ATCC Stag** | **BEI Stag** | **Zymo Stag** | **Grouped Even** | **Grouped Stag** |
| *Acinetobacter* | 5 | 5 | 0.18 | 0.22 | 0 | 5 | 0.13 |
| *Actinomyces* | 5 | 5 | 0.02 | 0.02 | 0 | 5 | 0.01 |
| *Bacillus* | 5 | 5 | 1.8 | 2.19 | 17.4 | 5 | 7.13 |
| *Bacteroides* | 5 | 5 | 0.02 | 0.02 | 0 | 5 | 0.01 |
| *Bifidobacterium* | 5 | 0 | 0.02 | 0 | 0 | 2.5 | 0.01 |
| *Clostridium* | 5 | 5 | 1.8 | 2.19 | 0 | 5 | 1.33 |
| *Cutibacterium/Propionibacterium* | 5 | 5 | 0.18 | 0.22 | 0 | 5 | 0.13 |
| *Deinococcus* | 5 | 5 | 0.02 | 0.02 | 0 | 5 | 0.01 |
| *Enterococcus* | 5 | 5 | 0.02 | 0.02 | 9.9 | 5 | 3.31 |
| *Escherichia* | 5 | 5 | 18 | 21.91 | 10.1 | 5 | 16.67 |
| *Helicobacter* | 5 | 5 | 0.18 | 0.22 | 0 | 5 | 0.13 |
| *Lactobacillus* | 5 | 5 | 0.18 | 0.22 | 18.4 | 5 | 6.27 |
| *Listeria* | 0 | 5 | 0 | 0.22 | 14.1 | 2.5 | 4.77 |
| *Neisseria* | 5 | 5 | 0.18 | 0.22 | 0 | 5 | 0.13 |
| *Porphyromonas* | 5 | 0 | 18 | 0 | 0 | 2.5 | 6.00 |
| *Pseudomonas* | 5 | 5 | 1.8 | 2.19 | 4.2 | 5 | 2.73 |
| *Rhodobacter* | 5 | 5 | 18 | 21.91 | 0 | 5 | 13.30 |
| *Salmonella* | 0 | 0 | 0 | 0 | 10.4 | 0 | 3.47 |
| *Staphylococcus* | 10 | 10 | 19.8 | 24.1 | 15.5 | 10 | 19.80 |
| *Streptococcus* | 10 | 15 | 19.8 | 24.102 | 0 | 12.5 | 14.63 |

Genomic DNA from American Type Culture Collection (ATCC, Manassas, VA) for even (ATCC® MSA-1002) and staggered (ATCC® MSA-1003) mock microbiome standard samples were used for analysis. The mock communities contain 20 common bacterial species [13, 14], that include both gram positive and gram-negative bacteria. Additional mock community sample sequencing information and expected abundances were compiled using the product information generously provided the external labs. Evenly distributed ATCC and BEI mock bacterial community samples, and staggered ATCC, BEI and Zymo mock samples were added to the even/staggered ATCC mock microbiome standard samples from the external institutions (**Table 1**).
