# Supplementary material for: Multi-amplicon microbiome data analysis pipelines for mixed orientation sequences using QIIME2: Assessing reference database, variable region and pre-processing bias in classification of mock bacterial community samples
Source: PLoS One. 2023 Jan 13;18(1):e0280293. doi: 10.1371/journal.pone.0280293 (PMC9838852; doi:10.1371/journal.pone.0280293)
Supplement: S2 Table — V: Variable region. Reads are presented as the average absolute number of reads and the percentage of the total reads for each sample. Total reads are the sum of all sequences/reads for each mock community sample (same for each sample regardless of workflow). Cutadapt searches the entire demultiplexed sequence file for each primer (forward or reverse) and discards all other reads; therefore, “no adapter” row was unable to be calculated for Cutadapt. °Summed V total is the total number of reads for regions V2, V3, V4, V67, V8 and V9 after Cutadapt or cutPrimers, respectively (note- “no adapter” sequences not included as this is not calculated with the Cutadapt pipeline). ¥ Cutadapt workflow performed without the “^” symbol preceding the primer sequence at the–p front flag (which only matches primers if they occur at the beginning of a read). *p < .05 between V regions (holding cutPrimers vs Cutadapt workflow constant); ǂp < .05 between cutPrimers vs Cutadapt workflows (holding V region constant). (DOCX) [file pone.0280293.s007.docx]

**Supplemental Table 2: Average Sequences per V Region Comparing CutPrimers versus Cutadapt (Forward Primer Amplicons Only and Forward + Reverse Primer Amplicons)**

| **V Region** | **Reads CutPrimers** | **Reads Cutadapt**  **F Only** | **Reads Cutadapt**  **F & R^¥^** | **Total Reads (Sample)** | **% Total Reads CutPrimers** | **% Total Reads Cutadapt (F Only)** | **% Total Reads Cutadapt (FR^¥^)** |
| --- | --- | --- | --- | --- | --- | --- | --- |
| **2** | 32,923.66 ± 21,356.37* | 44,598.36 ± 11,615.13* | 94,105.73 ± 58,020.67*^ǂ^ | 617,410.80 ± 452,297.61 | 5.98 ± 2.63 | 7.49 ± 4.85 | 17.33 ± 5.75 |
| **3** | 180,767.93 ± 133,940.63* | 181,773.90 ± 133,488.98 | 300,213.39 ± 216,744.57*^ǂ^ | 617,410.80 ± 452,297.61 | 29.19 ± 5.56 | 29.60 ± 5.65 | 49.35 ± 5.94 |
| **4** | 77,965.95 ± 56,527.49* | 79,196.95 ± 55,721.23* | 194,546.90 ± 149,442.02*^ǂ^ | 617,410.80 ± 452,297.61 | 12.80 ± 2.01 | 13.28 ± 2.11 | 30.40 ± 3.85 |
| **6-7** | 141,118.29 ± 119,970.73* | 144,510.41 ± 121,927.86* | 175,144.44 ± 138,952.35* | 617,410.80 ± 452,297.61 | 20.40 ± 5.83 | 21.11 ± 5.91 | 27.04 ± 3.49 |
| **8** | 89,393.07 ± 63,675.28* | 96,739.80 ± 71,689.65* | 178,019.85 ± 130,335.69*^ǂ^ | 617,410.80 ± 452,297.61 | 14.85 ± 3.67 | 16.35 ± 6.15 | 29.09 ± 5.26 |
| **9** | 41,814.22 ± 33,688.15* | 43,038.34 ± 34,245.34* | 53,508.17 ± 42,412.76* | 617,410.80 ± 452,297.61 | 6.47 ± 2.26 | 6.73 ± 2.39 | 8.49 ± 2.76 |
| **No adapter** | 53,427.68 ± 32897.05 | N/A | N/A | - | - | - | - |
| **Summed V Total°** | 563,983.12 ± 423,673.83 | 582,485.29 ± 431,025.69 | 995,538.49 ± 730,843.87 | - | 89.70 ± 5.86% | 94.03 ± 1.87% | 161.71 ± 7.70% |

V: Variable region. Reads are presented as the average absolute number of reads and the percentage of the total reads for each sample. Total reads are the sum of all sequences/reads for each mock community sample (same for each sample regardless of workflow). Cutadapt searches the entire demultiplexed sequence file for each primer (forward or reverse) and discards all other reads; therefore, “no adapter” row was unable to be calculated for Cutadapt. °Summed V total is the total number of reads for regions V2, V3, V4, V67, V8 and V9 after Cutadapt or cutPrimers, respectively (note- “no adapter” sequences not included as this is not calculated with the Cutadapt pipeline). ^¥^ Cutadapt workflow performed without the “^” symbol preceding the primer sequence at the –p front flag (which only matches primers if they occur at the beginning of a read). **p* < .05 between V regions (holding cutPrimers vs Cutadapt workflow constant); ^ǂ^*p* < .05 between cutPrimers vs Cutadapt workflows (holding V region constant).
