# Supplementary material for: Multi-amplicon microbiome data analysis pipelines for mixed orientation sequences using QIIME2: Assessing reference database, variable region and pre-processing bias in classification of mock bacterial community samples
Source: PLoS One. 2023 Jan 13;18(1):e0280293. doi: 10.1371/journal.pone.0280293 (PMC9838852; doi:10.1371/journal.pone.0280293)
Supplement: S3 Table — The average relative abundance of bacteria at the taxonomic level of genus that were not present in the mock bacterial communities, stratified by V region and reference database for both CutPrimers and Cutadapt-based pipelines. *Assigned higher is any feature that was annotated at a taxonomic level higher than genus (i.e. Family, Class etc.). If cell is blank that indicates this taxon was not annotated in that V region and reference database. (DOCX) [file pone.0280293.s008.docx]

**Supplemental Table 3: Bacteria Annotated in the Mock Community Feature Table That were Unexpected or Not Classified to the Taxonomic Level of Genus**

| **Genus** | **Average Annotated Relative Abundance (%) Stratified by V region and Reference Database** | | | | | | | | | | | | | | | | | |
| --- | --- | --- | --- | --- | --- | --- | --- | --- | --- | --- | --- | --- | --- | --- | --- | --- | --- | --- |
|  | **V2 GG** | **V2 RDP** | **V2 Silva** | **V3**  **GG** | **V3 RDP** | **V3 Silva** | **V4 GG** | **V4 RDP** | **V4 Silva** | **V67 GG** | **V67 RDP** | **V67 Silva** | **V8 GG** | **V8 RDP** | **V8 Silva** | **V9 GG** | **V9 RDP** | **V9 Silva** |
| ***CutPrimers Even*** | | | | | | | | | | | | | | | | | | |
| Assigned Higher* | 10.85 | 9.61 | .0006 | 6.72 | .17 | .0001 | 5.78 | 20.33 | 0 | 22.4 | 13.22 | 28.48 | 12.36 | 80.91 | 1.37 | 56.96 | 57.08 | 56.95 |
| *Corynebacterium* | .0003 |  |  |  |  |  |  |  |  |  |  |  |  |  |  |  |  |  |
| *Enterobacter* |  | 1.87 | 1.87 |  |  |  |  |  |  |  |  |  |  |  |  |  |  |  |
| *Finegoldia* | .0006 | .0006 | .0006 | .0004 | .0004 | .0004 |  |  |  |  |  |  |  |  |  |  |  |  |
| *Klebsiella* |  |  |  |  |  |  |  |  |  | 1.46 |  |  |  |  |  |  |  |  |
| *Lysobacter* |  |  |  |  |  |  | .0001 | .0001 | .0001 |  |  |  | .0002 |  | .0002 |  |  |  |
| ***CutPrimers Stag*** | | | | | | | | | | | | | | | | | | |
| Assigned Higher* | 10.20 | 12.44 | .0009 | 17.03 | 2.26 | .2458 | 8.30 | 64.48 | 0 | 28.72 | 14.43 | 20.75 | 20.68 | 85.56 | 2.11 | 94.91 | 95.22 | 94.90 |
| *Alistipes* |  |  |  |  | .0004 | .0004 |  |  |  |  | .0006 | .0006 |  |  |  |  |  |  |
| *Corynebacterium* |  |  |  |  |  |  | .0004 |  |  | .0004 |  |  |  |  |  |  |  |  |
| *Enterobacter* |  | 1.98 | 1.98 |  | 1.38 | 1.13 |  |  |  |  |  |  |  |  |  |  |  |  |
| *Ignatzschineria* |  |  |  |  |  |  |  |  |  | .0002 |  | .0004 |  |  |  |  |  |  |
| *Klebsiella* |  |  |  |  |  |  |  |  |  | 5.62 |  |  |  |  |  |  |  |  |
| *Lactococcus* |  |  |  | .0030 |  | .0030 |  |  |  |  |  |  |  |  |  |  |  |  |
| *Prevotella* |  |  |  | .0001 | .0001 | .0001 | .0009 | .0009 | .0009 |  |  |  |  |  |  |  |  |  |
| *Proteiniclasticum* |  |  |  |  |  |  |  |  |  |  | .0001 |  |  |  |  |  |  |  |
| *Pseudoxanthomonas* |  |  |  |  | .0010 | .0010 |  |  |  |  |  |  |  |  |  |  |  |  |
| *Rickettsia* | .0082 | .0082 | .0082 | .0072 | .0072 | .0072 |  |  |  | .0018 | .0018 | .0018 | .0014 | .0014 | .0014 |  |  |  |
| *Thioclava* |  |  |  |  |  |  |  |  |  |  |  |  |  | .0024 |  |  |  |  |

The average relative abundance of bacteria at the taxonomic level of genus that were not present in the mock bacterial communities, stratified by V region and reference database for both CutPrimers and Cutadapt-based pipelines. *Assigned higher is any feature that was annotated at a taxonomic level higher than genus (i.e. Family, Class etc.). If cell is blank that indicates this taxon was not annotated in that V region and reference database.
