# Supplementary material for: Multi-amplicon microbiome data analysis pipelines for mixed orientation sequences using QIIME2: Assessing reference database, variable region and pre-processing bias in classification of mock bacterial community samples
Source: PLoS One. 2023 Jan 13;18(1):e0280293. doi: 10.1371/journal.pone.0280293 (PMC9838852; doi:10.1371/journal.pone.0280293)
Supplement: S4 Table — Even mock samples n = 21 (atcc_even n = 18; bei_even n = 3). Staggered mock samples n = 20 (atcc_stag n = 14; bei_stag n = 4; zymo_stag n = 2). Non-parametric tests were run to determine distance metric differences between V region (Kruskal-Wallis), reference databases (Kruskal-Wallis), and bioinformatics workflows (Wilcoxon Rank Sum), respectively. *p < .05 between V regions (holding reference database and workflow constant); Φp < .05 between reference databases (holding V region and workflow constant); p < .05 between cutPrimers vs Cutadapt workflows (holding V region and reference database constant). Euclidean, Jensen-Shannon, and Bray-Curtis Dissimilarity scores range between 0–1 where a score of 0 indicates zero dissimilarity between expected and actual mock bacterial abundance (or that expected and actual abundance are identical) and a score of 1 indicates complete dissimilarity between actual and expected abundances. (DOCX) [file pone.0280293.s009.docx]

**Supplemental Table 4. Global Distance Metrics using CutPrimers**

| **Mock type, V region, Database** | **Euclidean** | **Jensen-Shannon** | **Bray-Curtis** |
| --- | --- | --- | --- |
| ***Even Mock Samples*** | | | |
| Even V2 Greengenes | 0.23 ± 0.07* | 0.37 ± 0.08*ᶲ | 0.39 ± 0.11*ᶲ |
| Even V2 Silva | 0.22 ± 0.05* | 0.36 ± 0.06*ᶲ | 0.38 ± 0.09*ᶲ |
| Even V2 RDP | 0.24 ± 0.04* | 0.43 ± 0.05*ᶲ | 0.44 ± 0.08*ᶲ |
| Even V3 Greengenes | 0.19 ± 0.05*ᶲ | 0.37 ± 0.05*ᶲ | 0.35 ± 0.08* |
| Even V3 Silva | 0.20 ± 0.04*ᶲ | 0.38 ± 0.03*ᶲ | 0.34 ± 0.07* |
| Even V3 RDP | 0.20 ± 0.04*ᶲ | 0.38 ± 0.03*ᶲ | 0.34 ± 0.07* |
| Even V4 Greengenes | 0.20 ± 0.06*ᶲ | 0.34 ± 0.06*ᶲ | 0.36 ± 0.08*ᶲ |
| Even V4 Silva | 0.21 ± 0.04*ᶲ | 0.36 ± 0.04*ᶲ | 0.36 ± 0.07*ᶲ |
| Even V4 RDP | 0.31 ± 0.04*ᶲ | 0.51 ± 0.03*ᶲ | 0.51 ± 0.05*ᶲ |
| Even V67 Greengenes | 0.36 ± 0.10*ᶲ | 0.51 ± 0.05*ᶲ | 0.54 ± 0.06* |
| Even V67 Silva | 0.38 ± 0.03*ᶲ | 0.52 ± 0.02*ᶲ | 0.54 ± 0.03* |
| Even V67 RDP | 0.34 ± 0.02*ᶲ | 0.55 ± 0.01*ᶲ | 0.56 ± 0.03* |
| Even V8 Greengenes | 0.28 ± 0.07*ᶲ | 0.47 ± 0.05*ᶲ | 0.51 ± 0.07*ᶲ |
| Even V8 Silva | 0.25 ± 0.07*ᶲ | 0.42 ± 0.06*ᶲ | 0.46 ± 0.08*ᶲ |
| Even V8 RDP | 0.86 ± 0.07*ᶲ | 0.78 ± 0.02*ᶲ | 0.91 ± 0.03*ᶲ |
| Even V9 Greengenes | 0.73 ± 0.02* | 0.77 ± 0.01* | 0.94 ± 0.00* |
| Even V9 Silva | 0.73 ± 0.02* | 0.78 ± 0.01* | 0.94 ± 0.00* |
| Even V9 RDP | 0.73 ± 0.02* | 0.78 ± 0.01* | 0.94 ± 0.00* |
| ***Stag Mock Samples*** | | | |
| Stag V2 Greengenes | 0.52 ± 0.10* | 0.48 ± 0.04*ᶲ | 0.54 ± 0.08*ᶲ |
| Stag V2 Silva | 0.47 ± 0.16* | 0.41 ± 0.09*ᶲ | 0.47 ± 0.13*ᶲ |
| Stag V2 RDP | 0.52 ± 0.17* | 0.48 ± 0.10*ᶲ | 0.54 ± 0.14*ᶲ |
| Stag V3 Greengenes | 0.28 ± 0.05*ᶲ | 0.38 ± 0.05*ᶲ | 0.30 ± 0.09*ᶲ |
| Stag V3 Silva | 0.13 ± 0.07*ᶲ | 0.21 ± 0.09*ᶲ | 0.17 ± 0.12*ᶲ |
| Stag V3 RDP | 0.15 ± 0.11*ᶲ | 0.23 ± 0.14*ᶲ | 0.19 ± 0.17*ᶲ |
| Stag V4 Greengenes | 0.57 ± 0.14*ᶲ | 0.48 ± 0.05*ᶲ | 0.56 ± 0.10*ᶲ |
| Stag V4 Silva | 0.52 ± 0.21*ᶲ | 0.40 ± 0.12*ᶲ | 0.50 ± 0.17*ᶲ |
| Stag V4 RDP | 0.73 ± 0.20*ᶲ | 0.63 ± 0.08*ᶲ | 0.71 ± 0.15*ᶲ |
| Stag V67 Greengenes | 0.42 ± 0.05*ᶲ | 0.53 ± 0.03*ᶲ | 0.49 ± 0.06*ᶲ |
| Stag V67 Silva | 0.31 ± 0.05*ᶲ | 0.40 ± 0.05*ᶲ | 0.32 ± 0.10*ᶲ |
| Stag V67 RDP | 0.29 ± 0.09*ᶲ | 0.42 ± 0.07*ᶲ | 0.34 ± 0.12*ᶲ |
| Stag V8 Greengenes | 0.44 ± 0.04*ᶲ | 0.50 ± 0.05*ᶲ | 0.50 ± 0.09*ᶲ |
| Stag V8 Silva | 0.35 ± 0.07*ᶲ | 0.36 ± 0.09*ᶲ | 0.35 ± 0.13*ᶲ |
| Stag V8 RDP | 0.94 ± 0.08*ᶲ | 0.77 ± 0.04*ᶲ | 0.86 ± 0.08*ᶲ |
| Stag V9 Greengenes | 1.04 ± 0.09* | 0.81 ± 0.03* | 0.98 ± 0.04* |
| Stag V9 Silva | 1.04 ± 0.09* | 0.81 ± 0.03* | 0.98 ± 0.04* |
| Stag V9 RDP | 1.05 ± 0.08* | 0.81 ± 0.02* | 0.98 ± 0.03* |

Even mock samples n = 21 (atcc_even n= 18; bei_even n= 3). Staggered mock samples n = 20 (atcc_stag n= 14; bei_stag n= 4; zymo_stag n= 2). Non-parametric tests were run to determine distance metric differences between V region (Kruskal-Wallis), reference databases (Kruskal-Wallis), and bioinformatics workflows (Wilcoxon Rank Sum), respectively. **p* < .05 between V regions (holding reference database and workflow constant); ᶲ*p* < .05 between reference databases (holding V region and workflow constant); *p* < .05 between cutPrimers vs Cutadapt workflows (holding V region and reference database constant). Euclidean, Jensen-Shannon, and Bray-Curtis Dissimilarity scores range between 0-1 where a score of 0 indicates zero dissimilarity between expected and actual mock bacterial abundance (or that expected and actual abundance are identical) and a score of 1 indicates complete dissimilarity between actual and expected abundances
