# Supplementary material for: Multi-amplicon microbiome data analysis pipelines for mixed orientation sequences using QIIME2: Assessing reference database, variable region and pre-processing bias in classification of mock bacterial community samples
Source: PLoS One. 2023 Jan 13;18(1):e0280293. doi: 10.1371/journal.pone.0280293 (PMC9838852; doi:10.1371/journal.pone.0280293)
Supplement: S5 Table — Even mock samples n = 21 (atcc_even n = 18 samples; bei_even n = 3 samples). n/a = Bacteria listed was not in the specified mock community. Values (mean or standard deviation) were rounded to two decimal places, and values < 0.005 were rounded to 0.0 (not true zero in every case). Taxon-specific agreement was defined as the observed/expected ratio and calculated as the observed relative abundance (%) / expected relative abundance (%) for each genus. A value of 1 indicates perfect agreement, a value under 0–0.999 indicates the actual relative abundance (%) is less than expected, and a value over 1 indicates the actual relative abundance (%) is higher than expected in the mock community for that individual taxon. Non-parametric tests were run to determine precision metric differences between V region (Kruskal-Wallis), reference databases (Kruskal-Wallis), and bioinformatics workflows (Wilcoxon Rank Sum), respectively, for each individual genus. *p < .05 between V regions (holding reference database and workflow constant). (DOCX) [file pone.0280293.s010.docx]

**Supplemental Table 5. Taxon-Specific Metrics for All Evenly-Spaced Mock Bacterial Communities**

**Evenly-Spaced Mock Bacterial Communities V2, V3, V4**

| **Genus (Expected Abundance %)** | **Even V2**  **GG** | **Even V2 Silva** | **Even V2 RDP** | **Even V3**  **GG** | **Even V3 Silva** | **Even V3 RDP** | **Even V4 GG** | **Even V4 Silva** | **Even V4 RDP** |
| --- | --- | --- | --- | --- | --- | --- | --- | --- | --- |
| **CutPrimers** | | | | | | | | | |
| Acinetobacter (5%) | 1.73± 0.62*ᶲ | 1.66 ± 0.48*ᶲ | 1.85 ± 0.55*ᶲ | 1.49 ± 0.35* | 1.50 ± 0.23* | 1.50 ± 0.23* | 1.69 ± 0.59*ᶲ | 1.74 ± 0.51*ᶲ | 2.23 ± 0.62*ᶲ |
| Actinomyces (5%) | 0.18± 0.07*ᶲ | 0.18 ± 0.07*ᶲ | 0.0 ± 0.0ᶲ | 0.01 ± 0.01*ᶲ | 0.01 ± 0.01*ᶲ | 0.0 ± 0.0ᶲ | 0.19 ± 0.10*ᶲ | 0.19 ± 0.09*ᶲ | 0.0 ± 0.0ᶲ |
| Bacillus (5%) | 1.44± 1.48* | 1.33 ± 1.24* | 1.48 ± 1.38* | 2.43 ± 1.46* | 2.40 ± 1.22* | 2.41 ± 1.22* | 2.01 ± 1.14*ᶲ | 2.03 ± 0.96*ᶲ | 0.0 ± 0.0*ᶲ |
| Bacteroides (5%) | 2.81 ± 1.54* | 2.82 ± 1.59* | 3.12 ± 1.75* | 2.30 ± 0.96* | 2.41 ± 1.05* | 2.41 ± 1.06* | 2.38 ± 1.11*ᶲ | 2.57 ± 1.27*ᶲ | 3.28 ± 1.58*ᶲ |
| Bifidobacterium (2.5%) | 0.61 ± 0.29*ᶲ | 0.62 ± 0.30*ᶲ | 0.0 ± 0.0ᶲ | 0.01 ± 0.01*ᶲ | 0.01 ± 0.01*ᶲ | 0.0 ± 0.0ᶲ | 0.47 ± 0.21*ᶲ | 0.51 ± 0.24*ᶲ | 0.0 ± 0.0ᶲ |
| Clostridium (5%) | 1.24 ± 0.51*ᶲ | 0.0 ± 0.0ᶲ | 0.0 ± 0.0ᶲ | 1.78 ± 0.76*ᶲ | 0.0 ± 0.0ᶲ | 0.0 ± 0.0ᶲ | 1.81 ± 0.87*ᶲ | 0.0 ± 0.0ᶲ | 0.0 ± 0.0ᶲ |
| Cutibacterium/ Propionibacterium (5%) | 0.18 ± 0.08*ᶲ | 0.18 ± 0.09*ᶲ | 0.0 ± 0.0ᶲ | 0.01 ± 0.01*ᶲ | 0.01 ± 0.01*ᶲ | 0.0 ± 0.0ᶲ | 0.03 ± 0.07*ᶲ | 0.03 ± 0.07*ᶲ | 0.0 ± 0.0ᶲ |
| Deinococcus (5%) | 2.01 ± 0.91* | 1.96 ± 0.81* | 2.18 ± 0.92* | 0.0 ± 0.0* | 0.0 ± 0.0* | 0.0 ± 0.0* | 0.14 ± 0.12*ᶲ | 0.15 ± 0.10*ᶲ | 0.19 ± 0.14*ᶲ |
| Enterococcus (5%) | 0.66 ± 0.69* | 0.61 ± 0.58* | 0.68 ± 0.65* | 0.98 ± 0.40*ᶲ | 0.97 ± 0.31*ᶲ | 0.97 ± 0.31*ᶲ | 0.60 ± 0.39*ᶲ | 0.60 ± 0.32*ᶲ | 0.78 ± 0.45*ᶲ |
| Escherichia-Shigella (5%) | 0.0 ± 0.0ᶲ | 1.94 ± 1.40*ᶲ | 0.94 ± 0.61*ᶲ | 0.0 ± 0.0ᶲ | 1.45 ± 0.59*ᶲ | 1.45 ± 0.59*ᶲ | 0.0 ± 0.0ᶲ | 1.15 ± 1.01*ᶲ | 1.49 ± 1.34*ᶲ |
| Helicobacter (5%) | 1.60 ± 0.84* | 1.60 ± 0.87* | 1.77 ± 0.95* | 0.56 ± 0.37* | 0.58 ± 0.38* | 0.58 ± 0.38* | 0.75 ± 0.41*ᶲ | 0.80 ± 0.44*ᶲ | 1.05 ± 0.63*ᶲ |
| Lactobacillus (5%) | 0.96 ± 0.39* | 0.95 ± 0.42* | 1.05 ± 0.45* | 1.53 ± 0.61* | 1.60 ± 0.67* | 1.61 ± 0.67* | 1.09 ± 0.41*ᶲ | 1.16 ± 0.47*ᶲ | 1.50 ± 0.63*ᶲ |
| Listeria (2.5%) | 0.57 ± 0.47* | 0.58 ± 0.48 | 0.61 ± 0.50* | 1.32 ± 0.07* | 1.36 ± 0.06 | 1.36 ± 0.06* | 0.0 ± 0.0*ᶲ | 0.50 ± 0.41ᶲ | 0.0 ± 0.0*ᶲ |
| Neisseria (5%) | 1.00 ± 0.25*ᶲ | 0.97 ± 0.27*ᶲ | 1.08± 0.28*ᶲ | 0.84 ± 0.09* | 0.86 ± 0.13* | 0.87 ± 0.13* | 1.15 ± 0.26*ᶲ | 1.20 ± 0.29*ᶲ | 1.54 ± 0.36*ᶲ |
| Porphyromonas (2.5%) | 0.69 ± 0.33* | 0.70 ± 0.34* | 0.77 ± 0.37* | 0.86 ± 0.24* | 0.89 ± 0.28* | 0.89 ± 0.28* | 1.42 ± 0.47*ᶲ | 1.53 ± 0.56*ᶲ | 1.92 ± 0.70*ᶲ |
| Pseudomonas (5%) | 1.75 ± 0.84*ᶲ | 1.62 ± 0.59*ᶲ | 1.82± 0.70*ᶲ | 1.30 ± 0.50* | 1.30 ± 0.37* | 1.30 ± 0.37* | 0.71 ± 0.45*ᶲ | 0.70 ± 0.37*ᶲ | 0.90 ± 0.45*ᶲ |
| Rhodobacter (5%) | 0.77 ± 0.46* | 0.70 ± 0.30* | 0.80± 0.39* | 0.79 ± 0.37* | 0.79 ± 0.28* | 0.79 ± 0.28* | 0.76 ± 0.60*ᶲ | 0.74 ± 0.45*ᶲ | 0.96 ± 0.62*ᶲ |
| Salmonella (0%) | n/a | n/a | n/a | n/a | n/a | n/a | n/a | n/a | n/a |
| Staphylococcus (10%) | 0.87 ± 0.88* | 0.80 ± 0.61* | 0.87 ± 0.81* | 1.04 ± 0.39*ᶲ | 1.04 ± 0.39*ᶲ | 0.87 ± 0.81*ᶲ | 0.87 ± 0.64*ᶲ | 0.89 ± 0.55*ᶲ | 0.06 ± 0.03*ᶲ |
| Streptococcus (12.5%) | 0.31 ± 0.25* | 0.29 ± 0.20* | 0.32 ± 0.23* | 1.49 ± 0.27* | 1.48 ± 0.27* | 0.32 ± 0.23* | 1.54 ± 0.58*ᶲ | 1.55 ± 0.40*ᶲ | 2.00 ± 0.52*ᶲ |

**Evenly-Spaced Mock Bacterial Communities V6-7, V8, V9**

| **Genus (Expected Abundance %)** | **Even V6-7 GG** | **Even V6-7 Silva** | **Even V6-7 RDP** | **Even V8 GG** | **Even V8 Silva** | **Even V8 RDP** | **Even V9 GG** | **Even V9 Silva** | **Even V9 RDP** |
| --- | --- | --- | --- | --- | --- | --- | --- | --- | --- |
| **CutPrimers** | | | | | | | | | |
| Acinetobacter (5%) | 0.02 ± 0.02* | 0.02 ± 0.01* | 0.02 ± 0.02* | 2.78±0.69*ᶲ | 2.47±0.62*ᶲ | 0.0 ±0.0*ᶲ | 19.69±0.15* | 19.69±0.15* | 19.74±0.15* |
| Actinomyces (5%) | 0.23 ± 0.24*ᶲ | 0.26 ±0.32*ᶲ | 0.0 ± 0.0ᶲ | 0.33±0.15*ᶲ | 0.30 ±0.14*ᶲ | 0.0 ± 0.0ᶲ | 0.0 ± 0.0* | 0.0 ± 0.0* | 0.0 ± 0.0 |
| Bacillus (5%) | 0.0 ± 0.0*ᶲ | 2.34±1.55*ᶲ | 2.42±1.70*ᶲ | 0.14±0.14*ᶲ | 0.13±0.10*ᶲ | 0.0 ±0.0*ᶲ | 0.02±0.06*ᶲ | 0.03±0.06*ᶲ | 0.0 ± 0.0*ᶲ |
| Bacteroides (5%) | 5.01±1.33*ᶲ | 5.60 ±2.18*ᶲ | 5.62 ±2.09*ᶲ | 0.0 ± 0.0* | 0.0 ± 0.0* | 0.0 ± 0.0* | 0.0 ± 0.0* | 0.0 ± 0.0* | 0.0 ± 0.0* |
| Bifidobacterium (2.5%) | 0.25 ± 0.10*ᶲ | 0.28 ± 0.14*ᶲ | 0.0 ± 0.0ᶲ | 0.68±0.37*ᶲ | 0.63±0.35*ᶲ | 0.0 ± 0.0ᶲ | 0.0 ± 0.0* | 0.0 ± 0.0* | 0.0 ± 0.0 |
| Clostridium (5%) | 4.05 ±1.63*ᶲ | 0.0 ± 0.0ᶲ | 0.0 ± 0.0ᶲ | 0.01±0.01*ᶲ | 0.0 ± 0.0ᶲ | 0.0 ± 0.0ᶲ | 0.0 ± 0.0* | 0.0 ± 0.0 | 0.0 ± 0.0 |
| Cutibacterium/  Propionibacterium (5%) | 0.39 ± 0.31*ᶲ | 0.47 ± 0.41*ᶲ | 0.0 ± 0.0ᶲ | 1.29±0.65*ᶲ | 1.17±0.60*ᶲ | 0.0 ± 0.0ᶲ | 0.0 ± 0.0* | 0.0 ± 0.0* | 0.0 ± 0.0 |
| Deinococcus (5%) | 1.00 ± 0.58*ᶲ | 0.99 ± 0.27*ᶲ | 1.01 ±0.30*ᶲ | 1.46±0.52*ᶲ | 1.31 ±0.48*ᶲ | 5.99±2.78*ᶲ | 0.04 ± 0.03* | 0.04 ± 0.03* | 0.04 ± 0.03* |
| Enterococcus (5%) | 1.00 ±0.97*ᶲ | 0.0 ± 0.0*ᶲ | 0.0 ± 0.0*ᶲ | 1.00±0.42*ᶲ | 0.88±0.33*ᶲ | 0.0 ± 0.0*ᶲ | 0.01 ± 0.05* | 0.01 ± 0.05* | 0.01 ± 0.05* |
| Escherichia-Shigella (5%) | 0.0 ± 0.0ᶲ | 2.45 ± 1.21*ᶲ | 2.53 ±1.40*ᶲ | 0.0 ± 0.0ᶲ | 2.32±0.71*ᶲ | 0.0 ± 0.0*ᶲ | 0.0 ± 0.0 | 0.0 ± 0.0* | 0.0 ± 0.0* |
| Helicobacter (5%) | 0.56 ± 0.35*ᶲ | 0.65 ±0.49*ᶲ | 0.65 ±0.50*ᶲ | 0.08±0.10*ᶲ | 0.07±0.08*ᶲ | 2.27±5.76*ᶲ | 0.0 ± 0.0* | 0.0 ± 0.0* | 0.0 ± 0.0* |
| Lactobacillus (5%) | 1.48 ± 0.34*ᶲ | 0.0 ± 0.0*ᶲ | 1.64 ±0.61*ᶲ | 0.0 ± 0.0* | 0.0 ± 0.0* | 0.0 ± 0.0* | 0.0 ± 0.0* | 0.0 ± 0.0* | 0.0 ± 0.0* |
| Listeria (2.5%) | 2.73 ± 0.19* | 3.50 ± 0.36 | 3.59 ± 0.32* | 0.0 ± 0.0*ᶲ | 1.28 ± 1.08ᶲ | 0.0 ± 0.0*ᶲ | 0.0 ± 0.0* | 0.0 ± 0.0 | 0.0 ± 0.0* |
| Neisseria (5%) | 0.0 ± 0.0* | 0.0 ± 0.0* | 0.0 ± 0.0* | 1.93 ±0.53*ᶲ | 1.73 ±0.52*ᶲ | 0.0 ± 0.0*ᶲ | 0.0 ± 0.0*ᶲ | 0.0 ± 0.0*ᶲ | 0.0 ± 0.0*ᶲ |
| Porphyromonas (2.5%) | 1.51 ± 0.29* | 1.55 ± 0.32* | 1.57 ± 0.29* | 0.0 ± 0.0* | 0.0 ± 0.0* | 0.0 ± 0.0* | 0.0 ± 0.0* | 0.0 ± 0.0* | 0.0 ± 0.0 |
| Pseudomonas (5%) | 0.02 ± 0.08*ᶲ | 0.79 ± 0.71*ᶲ | 0.01 ± 0.04*ᶲ | 1.87 ±0.61*ᶲ | 1.65 ±0.47*ᶲ | 0.0 ± 0.0*ᶲ | 0.02 ± 0.01* | 0.02 ± 0.01* | 0.02 ± 0.01* |
| Rhodobacter (5%) | 0.0 ± 0.0* | 0.0 ± 0.0* | 0.0 ± 0.0* | 3.27 ±2.14*ᶲ | 2.61 ±2.13*ᶲ | 11.74±4.54*ᶲ | 0.15 ± 0.09* | 0.15 ± 0.09* | 0.15 ± 0.09* |
| Salmonella (0%) | n/a | n/a | n/a | n/a | n/a | n/a | n/a | n/a | n/a |
| Staphylococcus (10%) | 0.89 ± 0.23*ᶲ | 0.99 ± 0.35*ᶲ | 0.92 ± 0.32*ᶲ | 0.17 ±0.12*ᶲ | 0.15 ±0.10*ᶲ | 0.0 ± 0.0*ᶲ | 0.01 ± 0.01* | 0.01 ± 0.01* | 0.01 ± 0.01* |
| Streptococcus (12.5%) | 1.25 ± 0.75*ᶲ | 1.17 ± 0.23*ᶲ | 1.20 ± 0.29*ᶲ | 2.30 ±0.32*ᶲ | 2.04 ±0.25*ᶲ | 0.0 ± 0.0*ᶲ | 0.02±0.01*ᶲ | 0.02±0.01*ᶲ | 0.01±0.01*ᶲ |

Even mock samples n = 21 (atcc_even n= 18 samples; bei_even n= 3 samples). n/a = Bacteria listed was not in the specified mock community. Values (mean or standard deviation) were rounded to two decimal places, and values < 0.005 were rounded to 0.0 (not true zero in every case). Taxon-specific agreement was defined as the observed/expected ratio and calculated as the observed relative abundance (%) / expected relative abundance (%) for each genus. A value of 1 indicates perfect agreement, a value under 0-0.999 indicates the actual relative abundance (%) is less than expected, and a value over 1 indicates the actual relative abundance (%) is higher than expected in the mock community for that individual taxon. Non-parametric tests were run to determine precision metric differences between V region (Kruskal-Wallis), reference databases (Kruskal-Wallis), and bioinformatics workflows (Wilcoxon Rank Sum), respectively, for each individual genus. **p* < .05 between V regions (holding reference database and workflow constant).
