# Supplementary material for: Multi-amplicon microbiome data analysis pipelines for mixed orientation sequences using QIIME2: Assessing reference database, variable region and pre-processing bias in classification of mock bacterial community samples
Source: PLoS One. 2023 Jan 13;18(1):e0280293. doi: 10.1371/journal.pone.0280293 (PMC9838852; doi:10.1371/journal.pone.0280293)
Supplement: S6 Table — Staggered mock samples n = 20 (atcc_stag n = 14 samples; bei_stag n = 4 samples; zymo_stag n = 2 samples). n/a = Bacteria listed was not in the specified mock community. Values (mean or standard deviation) were rounded to two decimal places, and values < 0.005 were rounded to 0.0 (not true zero in every case). Taxon-specific agreement was defined as the observed/expected ratio and calculated as the observed relative abundance (%) / expected relative abundance (%) for each genus. A value of 1 indicates perfect agreement, a value under 0–0.999 indicates the actual relative abundance (%) is less than expected, and a value over 1 indicates the actual relative abundance (%) is higher than expected in the mock community for that individual taxon. Non-parametric tests were run to determine precision metric differences between V region (Kruskal-Wallis), reference databases (Kruskal-Wallis), and bioinformatics workflows (Wilcoxon Rank Sum), respectively, for each individual genus. *p < .05 between V regions (holding reference database and workflow constant); Φp < .05 between reference databases (holding V region and workflow constant). (DOCX) [file pone.0280293.s011.docx]

**Supplemental Table 6.** **Accuracy Metrics for All Staggered Mock Bacterial Communities**

**Staggered Mock Communities V2, V3, V4**

| **Genus (Expected Abundance %)** | **Stag V2 GG** | **Stag V2 Silva** | **Stag V2 RDP** | **Stag V3 GG** | **Stag V3 Silva** | **Stag V3 RDP** | **Stag V4 GG** | **Stag V4 Silva** | **Stag V4 RDP** |
| --- | --- | --- | --- | --- | --- | --- | --- | --- | --- |
| **CutPrimers** | | | | | | | | | |
| Acinetobacter (0.13%) | 2.38 ± 6.80* | 2.34 ± 7.01* | 2.80 ± 8.06* | 3.06±5.65*ᶲ | 2.89±5.84*ᶲ | 2.89±5.85*ᶲ | 1.65±5.02*ᶲ | 1.60±4.92*ᶲ | 3.23±6.75*ᶲ |
| Actinomyces (0.01%) | 2.38 ± 9.95* | 2.44 ±10.24* | 0.0 ± 0.0 | 0.25 ± 1.03* | 0.25 ± 1.05* | 0.0 ± 0.0 | 3.20±13.28* | 3.13 ±13.02* | 0.0 ± 0.0 |
| Bacillus (7.13%) | 0.60 ± 0.85* | 0.54 ± 0.80* | 0.66 ±0.92* | 1.88 ±0.39*ᶲ | 1.66±0.43*ᶲ | 1.55±0.65*ᶲ | 0.66±0.86*ᶲ | 0.58 ±0.78*ᶲ | 0.11±0.36*ᶲ |
| Bacteroides (0.01%) | 31.54±103.05* | 31.80±106.23* | 38.41±122.16* | 24.39±92.28* | 24.57±94.50* | 24.60±94.66* | 25.49±103.97*ᶲ | 24.97±101.89*ᶲ | 37.93±144.06*ᶲ |
| Bifidobacterium (0.01%) | 0.58 ± 0.53*ᶲ | 0.56 ± 0.51*ᶲ | 0.0 ± 0.0ᶲ | 0.00 ± 0.02* | 0.00 ± 0.01* | 0.0 ± 0.0 | 0.0 ± 0.0* | 0.0 ± 0.0* | 0.0 ± 0.0 |
| Clostridium (1.33%) | 0.79 ± 0.85*ᶲ | 0.0 ± 0.0ᶲ | 0.0 ± 0.0ᶲ | 2.72 ± 0.71*ᶲ | 0.0 ± 0.0ᶲ | 0.0 ± 0.0ᶲ | 0.76 ± 0.84*ᶲ | 0.0 ± 0.0ᶲ | 0.0 ± 0.0ᶲ |
| Cutibacterium/ Propionibacterium (0.13%) | 0.50 ± 1.32*ᶲ | 0.49 ± 1.36*ᶲ | 0.0 ± 0.0ᶲ | 0.03 ± 0.08*ᶲ | 0.03 ± 0.08*ᶲ | 0.0 ± 0.0ᶲ | 0.12 ± 0.40* | 0.11 ± 0.39* | 0.0 ± 0.0 |
| Deinococcus (0.01%) | 19.05 ± 61.96* | 19.04 ± 63.87* | 22.81 ± 73.45* | 0.0 ± 0.0* | 0.0 ± 0.0* | 0.0 ± 0.0* | 1.46 ± 5.96* | 1.43 ± 5.84* | 2.04 ± 8.29* |
| Enterococcus (3.31%) | 14.28 ± 63.22* | 14.67 ± 65.08* | 16.90 ± 74.98* | 16.10±67.63* | 16.32±69.23* | 16.43±69.33* | 11.54 ± 50.77* | 11.24 ± 49.77* | 15.96 ± 70.66* |
| Escherichia-Shigella (16.67%) | 0.0 ± 0.0ᶲ | 0.51 ± 0.49*ᶲ | 0.26 ± 0.22*ᶲ | 0.0 ± 0.0ᶲ | 0.96 ± 0.36*ᶲ | 1.03 ± 0.53*ᶲ | 0.0 ± 0.0ᶲ | 0.37 ± 0.50*ᶲ | 0.84 ± 0.57*ᶲ |
| Helicobacter (0.13%) | 5.13 ± 7.72* | 4.92 ± 7.95* | 6.22 ± 9.29* | 2.57 ± 6.72* | 2.48 ± 6.90* | 2.48 ± 6.91* | 1.88 ± 5.95*ᶲ | 1.81 ± 5.83*ᶲ | 3.03 ± 8.17*ᶲ |
| Lactobacillus (6.27%) | 3.16 ± 10.74* | 3.12 ± 11.07* | 3.66 ± 12.74* | 3.08 ± 3.80*ᶲ | 2.84 ± 3.94*ᶲ | 2.80 ± 3.97*ᶲ | 2.28 ± 7.27*ᶲ | 2.14 ± 7.13*ᶲ | 3.56 ± 9.99*ᶲ |
| Listeria (4.77%) | 3.59 ± 7.44* | 3.58 ± 7.70* | 4.13 ± 8.87* | 5.51 ± 11.43* | 5.50 ± 11.76* | 5.56 ± 11.75* | 0.0 ± 0.0*ᶲ | 5.02 ± 10.87*ᶲ | 0.0 ± 0.0*ᶲ |
| Neisseria (0.13%) | 2.01 ± 5.45* | 1.94 ± 5.61* | 2.30 ± 6.45* | 2.16 ± 4.92*ᶲ | 2.07 ± 5.07*ᶲ | 2.07 ± 5.08*ᶲ | 2.02 ± 6.10*ᶲ | 1.94 ± 5.97*ᶲ | 3.58 ± 8.25*ᶲ |
| Porphyromonas (6%) | 1.07 ± 0.16* | 1.03 ± 0.15* | 1.44 ± 0.91* | 1.10 ± 0.05*ᶲ | 0.97 ± 0.06*ᶲ | 0.97 ± 0.06*ᶲ | 0.51 ± 0.13*ᶲ | 0.51 ± 0.12*ᶲ | 2.41 ± 0.10*ᶲ |
| Pseudomonas (2.73%) | 1.32 ± 1.60* | 1.14 ± 1.28* | 1.42 ± 1.48* | 1.63 ± 0.90*ᶲ | 1.42 ± 0.76*ᶲ | 1.54 ± 1.07*ᶲ | 0.60 ± 0.99*ᶲ | 0.46 ± 0.69*ᶲ | 0.84 ± 0.86*ᶲ |
| Rhodobacter (13.3%) | 0.30 ± 0.30* | 0.26 ± 0.21* | 0.34 ± 0.30* | 0.74 ± 0.16*ᶲ | 0.65 ± 0.13*ᶲ | 0.65 ± 0.13*ᶲ | 0.18 ± 0.21*ᶲ | 0.16 ± 0.17*ᶲ | 0.48 ± 0.17*ᶲ |
| Salmonella (3.47%) | 2.95 ± 0.13 | 2.50 ± 0.08 | 2.81 ± 0.12 | 0.0 ± 0.0 | 0.0 ± 0.0 | 0.0 ± 0.0 | 0.0 ± 0.0 | 1.85 ± 0.12 | 0.30 ± 0.01 |
| Staphylococcus (19.8%) | 2.92 ± 1.31* | 2.76 ± 1.32* | 2.46 ± 1.55* | 1.34 ± 0.21*ᶲ | 1.17 ± 0.19*ᶲ | 1.20 ± 0.15*ᶲ | 3.18 ± 1.40*ᶲ | 3.08 ± 1.47*ᶲ | 0.03 ± 0.05*ᶲ |
| Streptococcus (14.63%) | 0.21 ± 0.33* | 0.17 ± 0.26* | 0.21 ± 0.29* | 1.32 ± 0.17*ᶲ | 1.15 ± 0.11*ᶲ | 1.15 ± 0.11*ᶲ | 0.62 ± 0.50*ᶲ | 0.58 ± 0.43*ᶲ | 1.83 ± 0.28*ᶲ |

**Staggered Mock Communities V6-7, V8, V9**

| **Genus (Expected**  **Abundance %)** | **Stag V6-7 GG** | **Stag V6-7 Silva** | **Stag V6-7 RDP** | **Stag V8 GG** | **Stag V8 Silva** | **Stag V8 RDP** | **Stag V9 GG** | **Stag V9 Silva** | **Stag V9 RDP** |
| --- | --- | --- | --- | --- | --- | --- | --- | --- | --- |
| **CutPrimers** | | | | | | | | | |
| Acinetobacter (0.13%) | 0.19 ± 0.78* | 0.21 ± 0.89* | 0.21 ± 0.87* | 4.40±11.25*ᶲ | 3.72±9.64*ᶲ | 0.0 ± 0.0*ᶲ | 304.13±42.40* | 302.48±42.07* | 304.90±41.68* |
| Actinomyces (0.01%) | 3.89±14.19*ᶲ | 4.27±16.25*ᶲ | 0.0 ± 0.0ᶲ | 7.67±30.85*ᶲ | 6.53±26.42*ᶲ | 0.0 ± 0.0ᶲ | 0.0 ± 0.0* | 0.0 ± 0.0* | 0.0 ± 0.0 |
| Bacillus (7.13%) | 0.11 ± 0.38*ᶲ | 1.62 ± 0.62*ᶲ | 1.65±0.57*ᶲ | 0.08 ± 0.12*ᶲ | 0.07 ± 0.10*ᶲ | 0.0±0.0*ᶲ | 0.15 ± 0.47*ᶲ | 0.19 ± 0.48*ᶲ | 0.0 ± 0.0*ᶲ |
| Bacteroides (0.01%) | 42.78±144.98*ᶲ | 46.15±166.48*ᶲ | 45.38±162.82*ᶲ | 0.0 ± 0.0* | 0.0 ± 0.0* | 0.0±0.0* | 0.0 ± 0.0* | 0.0 ± 0.0* | 0.0 ± 0.0* |
| Bifidobacterium (0.01%) | 0.27 ± 0.23*ᶲ | 0.23 ± 0.19*ᶲ | 0.0 ± 0.0ᶲ | 0.33 ± 0.30*ᶲ | 0.29 ± 0.28*ᶲ | 0.0 ±0.0ᶲ | 0.0 ± 0.0* | 0.0 ± 0.0* | 0.0 ± 0.0 |
| Clostridium (1.33%) | 7.33 ± 1.39*ᶲ | 0.0 ± 0.0ᶲ | 0.0 ± 0.0ᶲ | 0.01 ± 0.02*ᶲ | 0.0 ± 0.0ᶲ | 0.0 ±0.0ᶲ | 0.0 ± 0.0* | 0.0 ± 0.0 | 0.0 ± 0.0 |
| Cutibacterium/  Propionibacterium (0.13%) | 1.41 ± 1.88*ᶲ | 1.28 ± 2.10*ᶲ | 0.0 ± 0.0ᶲ | 3.33 ± 9.26*ᶲ | 2.79 ± 7.94*ᶲ | 0.0 ±0.0ᶲ | 0.0 ± 0.0* | 0.0 ± 0.0* | 0.0 ± 0.0 |
| Deinococcus (0.01%) | 13.99 ± 57.36* | 15.88 ± 65.65* | 15.55 ± 64.23* | 5.66±21.17*ᶲ | 4.82±18.13*ᶲ | 52.81±214.20*ᶲ | 14.48 ± 61.44* | 14.48 ± 61.44* | 16.22 ± 68.80* |
| Enterococcus (3.31%) | 15.06 ± 64.99*ᶲ | 0.0 ± 0.0*ᶲ | 0.0 ± 0.0*ᶲ | 10.86±46.27*ᶲ | 9.24±39.62*ᶲ | 0.0 ± 0.0*ᶲ | 8.76 ± 39.17* | 8.76 ± 39.17* | 9.81 ± 43.87* |
| Escherichia-Shigella (16.67%) | 0.0 ± 0.0ᶲ | 1.49 ± 0.73*ᶲ | 1.59 ± 0.98*ᶲ | 0.0 ± 0.0ᶲ | 1.20 ± 0.56*ᶲ | 0.0 ± 0.0*ᶲ | 0.0 ± 0.0 | 0.0 ± 0.0* | 0.00 ± 0.02* |
| Helicobacter (0.13%) | 2.46 ± 5.16*ᶲ | 2.39 ± 5.91*ᶲ | 2.37 ± 5.78*ᶲ | 0.30 ± 0.88*ᶲ | 0.24 ± 0.76*ᶲ | 64.13±179.00*ᶲ | 0.07 ± 0.29* | 0.07 ± 0.29* | 0.08 ± 0.33* |
| Lactobacillus (6.27%) | 4.27 ± 9.72*ᶲ | 0.13 ± 0.43*ᶲ | 4.21 ± 10.99*ᶲ | 0.01 ± 0.04* | 0.01 ± 0.03* | 0.0 ± 0.0* | 0.0 ± 0.0* | 0.0 ± 0.0* | 0.0 ± 0.0* |
| Listeria (4.77%) | 11.19 ± 20.73* | 11.88 ± 24.11* | 11.74 ± 23.53* | 0.0 ± 0.0*ᶲ | 5.85±12.37*ᶲ | 0.0 ± 0.0*ᶲ | 0.0 ± 0.0* | 0.0 ± 0.0* | 0.0 ± 0.0* |
| Neisseria (0.13%) | 0.0 ± 0.0* | 0.0 ± 0.0* | 0.0 ± 0.0* | 3.87 ± 10.00*ᶲ | 3.26 ± 8.57*ᶲ | 0.0 ± 0.0*ᶲ | 0.0 ± 0.0* | 0.0 ± 0.0* | 0.0 ± 0.0* |
| Porphyromonas (6%) | 2.05 ± 0.10*ᶲ | 1.74 ± 0.09*ᶲ | 1.76 ± 0.09*ᶲ | 0.0 ± 0.0* | 0.0 ± 0.0* | 0.0 ± 0.0* | 0.02 ± 0.03*ᶲ | 0.06 ± 0.02*ᶲ | 0.06 ± 0.02*ᶲ |
| Pseudomonas (2.73%) | 0.0 ± 0.0*ᶲ | 0.84 ± 0.83*ᶲ | 0.0 ± 0.0*ᶲ | 1.72 ± 1.68*ᶲ | 1.32 ± 1.14*ᶲ | 0.0 ± 0.0*ᶲ | 2.19 ± 5.92* | 2.19 ± 5.92* | 2.17 ± 5.93* |
| Rhodobacter (13.3%) | 0.0 ± 0.0* | 0.0 ± 0.0* | 0.0 ± 0.0* | 1.21 ± 0.31*ᶲ | 0.84 ± 0.36*ᶲ | 4.65 ± 1.79*ᶲ | 1.55 ± 0.57* | 1.54 ± 0.56* | 1.54 ± 0.56* |
| Salmonella (3.47%) | 0.0 ± 0.0 | 0.0 ± 0.0 | 0.0 ± 0.0 | 4.54 ± 0.36 | 3.09 ± 0.16 | n/a | 0.0 ± 0.0 | 0.0 ± 0.0 | 0.0 ± 0.0 |
| Staphylococcus (19.8%) | 1.34 ± 0.26*ᶲ | 1.10 ± 0.20*ᶲ | 1.04 ± 0.34*ᶲ | 2.16 ± 1.32*ᶲ | 1.84 ± 1.17*ᶲ | 0.0 ± 0.0*ᶲ | 0.42 ± 0.56* | 0.42 ± 0.56* | 0.42 ± 0.56* |
| Streptococcus (14.63%) | 1.17 ± 0.34*ᶲ | 0.97 ± 0.21*ᶲ | 0.98 ± 0.22*ᶲ | 1.11 ± 0.59*ᶲ | 0.89 ± 0.42*ᶲ | 0.0 ± 0.0*ᶲ | 0.28 ± 0.19* | 0.27 ± 0.19* | 0.26 ± 0.19* |

Staggered mock samples n = 20 (atcc_stag n= 14 samples; bei_stag n= 4 samples; zymo_stag n= 2 samples). n/a = Bacteria listed was not in the specified mock community. Values (mean or standard deviation) were rounded to two decimal places, and values < 0.005 were rounded to 0.0 (not true zero in every case). Taxon-specific agreement was defined as the observed/expected ratio and calculated as the observed relative abundance (%) / expected relative abundance (%) for each genus. A value of 1 indicates perfect agreement, a value under 0-0.999 indicates the actual relative abundance (%) is less than expected, and a value over 1 indicates the actual relative abundance (%) is higher than expected in the mock community for that individual taxon. Non-parametric tests were run to determine precision metric differences between V region (Kruskal-Wallis), reference databases (Kruskal-Wallis), and bioinformatics workflows (Wilcoxon Rank Sum), respectively, for each individual genus. **p* < .05 between V regions (holding reference database and workflow constant); ᶲ*p* < .05 between reference databases (holding V region and workflow constant
