# Supplementary material for: Multi-amplicon microbiome data analysis pipelines for mixed orientation sequences using QIIME2: Assessing reference database, variable region and pre-processing bias in classification of mock bacterial community samples
Source: PLoS One. 2023 Jan 13;18(1):e0280293. doi: 10.1371/journal.pone.0280293 (PMC9838852; doi:10.1371/journal.pone.0280293)
Supplement: S7 Table — Even mock samples n = 21 (atcc_even n = 18 samples; bei_even n = 3 samples). Staggered mock samples n = 20 (atcc_stag n = 14 samples; bei_stag n = 4 samples; zymo_stag n = 2 samples). Euclidean, Jensen-Shannon, and Bray-Curtis Dissimilarity scores range between 0–1 where a score of 0 indicates zero dissimilarity between expected and actual mock bacterial abundance (or that expected and actual abundance are identical) and a score of 1 indicates complete dissimilarity between actual and expected abundances. (DOCX) [file pone.0280293.s012.docx]

**Supplemental Table 7. Global Distance Metrics by Mock Type**

| **Mock type, V region, Database** | **Euclidean** | **Jensen-Shannon** | **Bray-Curtis** |
| --- | --- | --- | --- |
| ***Even Mock Community Samples*** | | | |
| Even_atcc V2 Greengenes | 0.23 ± 0.05 | 0.37 ± 0.06 | 0.39 ± 0.08 |
| Even_atcc V2 Silva | 0.22 ± 0.03 | 0.36 ± 0.04 | 0.38 ± 0.07 |
| Even_atcc V2 RDP | 0.25 ± 0.02 | 0.44 ± 0.02 | 0.45 ± 0.04 |
| Even_bei V2 Greengenes | 0.24 ± 0.19 | 0.36 ± 0.18 | 0.35 ± 0.23 |
| Even_bei V2 Silva | 0.21 ± 0.14 | 0.35 ± 0.15 | 0.35 ± 0.23 |
| Even_bei V2 RDP | 0.21 ± 0.11 | 0.40 ± 0.13 | 0.38 ± 0.20 |
| Even_atcc V3 Greengenes | 0.20 ± 0.05 | 0.38 ± 0.04 | 0.36 ± 0.08 |
| Even_atcc V3 Silva | 0.21 ± 0.04 | 0.39 ± 0.02 | 0.35 ± 0.06 |
| Even_atcc V3 RDP | 0.21 ± 0.04 | 0.39 ± 0.02 | 0.36 ± 0.06 |
| Even_bei V3 Greengenes | 0.12 ± 0.00 | 0.29 ± 0.00 | 0.22 ± 0.00 |
| Even_bei V3 Silva | 0.14 ± 0.00 | 0.30 ± 0.00 | 0.23 ± 0.00 |
| Even_bei V3 RDP | 0.14 ± 0.00 | 0.32 ± 0.00 | 0.23 ± 0.00 |
| Even_atcc V4 Greengenes | 0.20 ± 0.04 | 0.34 ± 0.04 | 0.35 ± 0.05 |
| Even_atcc V4 Silva | 0.21 ± 0.02 | 0.36 ± 0.01 | 0.36 ± 0.03 |
| Even_atcc V4 RDP | 0.30 ± 0.02 | 0.50 ± 0.01 | 0.50 ± 0.02 |
| Even_bei V4 Greengenes | 0.22 ± 0.13 | 0.38 ± 0.14 | 0.40 ± 0.20 |
| Even_bei V4 Silva | 0.22 ± 0.11 | 0.36 ± 0.12 | 0.38 ± 0.19 |
| Even_bei V4 RDP | 0.38 ± 0.05 | 0.55 ± 0.05 | 0.56 ± 0.13 |
| Even_atcc V67 Greengenes | 0.38 ± 0.09 | 0.52 ± 0.04 | 0.55 ± 0.04 |
| Even_atcc V67 Silva | 0.38 ± 0.03 | 0.52 ± 0.02 | 0.54 ± 0.03 |
| Even_atcc V67 RDP | 0.35 ± 0.02 | 0.55 ± 0.01 | 0.56 ± 0.03 |
| Even_bei V67 Greengenes | 0.23 ± 0.00 | 0.42 ± 0.00 | 0.41 ± 0.01 |
| Even_bei V67 Silva | 0.35 ± 0.01 | 0.48 ± 0.01 | 0.46 ± 0.00 |
| Even_bei V67 RDP | 0.30 ± 0.00 | 0.52 ± 0.00 | 0.52 ± 0.00 |
| Even_atcc V8 Greengenes | 0.27 ± 0.03 | 0.47 ± 0.03 | 0.51 ± 0.04 |
| Even_atcc V8 Silva | 0.25 ± 0.02 | 0.42 ± 0.03 | 0.46 ± 0.04 |
| Even_atcc V8 RDP | 0.86 ± 0.06 | 0.78 ± 0.02 | 0.91 ± 0.03 |
| Even_bei V8 Greengenes | 0.36 ± 0.18 | 0.49 ± 0.11 | 0.52 ± 0.18 |
| Even_bei V8 Silva | 0.31 ± 0.21 | 0.40 ± 0.16 | 0.44 ± 0.21 |
| Even_bei V8 RDP | 0.85 ± 0.11 | 0.78 ± 0.00 | 0.91 ± 0.03 |
| Even_atcc V9 Greengenes | 0.73 ± 0.02 | 0.77 ± 0.01 | 0.94 ± 0.00 |
| Even_atcc V9 Silva | 0.73 ± 0.02 | 0.77 ± 0.01 | 0.94 ± 0.00 |
| Even_atcc V9 RDP | 0.73 ± 0.02 | 0.78 ± 0.01 | 0.94 ± 0.00 |
| Even_bei V9 Greengenes | 0.72 ± 0.00 | 0.77 ± 0.00 | 0.94 ± 0.00 |
| Even_bei V9 Silva | 0.72 ± 0.00 | 0.77 ± 0.00 | 0.94 ± 0.00 |
| Even_bei V9 RDP | 0.73 ± 0.01 | 0.78 ± 0.01 | 0.94 ± 0.01 |
| ***Staggered Mock Community Samples*** | | | |
| Stag_atcc V2 Greengenes | 0.58 ± 0.02 | 0.49 ± 0.01 | 0.57 ± 0.01 |
| Stag_atcc V2 Silva | 0.57 ± 0.02 | 0.44 ± 0.01 | 0.53 ± 0.01 |
| Stag_atcc V2 RDP | 0.61 ± 0.09 | 0.49 ± 0.08 | 0.58 ± 0.08 |
| Stag_bei V2 Greengenes | 0.40 ± 0.03 | 0.48 ± 0.08 | 0.47 ± 0.16 |
| Stag_bei V2 Silva | 0.22 ± 0.12 | 0.31 ± 0.17 | 0.32 ± 0.24 |
| Stag_bei V2 RDP | 0.29 ± 0.11 | 0.41 ± 0.15 | 0.42 ± 0.24 |
| Stag_zymo V2 Greengenes | 0.36 ± 0.01 | 0.45 ± 0.00 | 0.48 ± 0.02 |
| Stag_zymo V2 Silva | 0.29 ± 0.00 | 0.35 ± 0.00 | 0.38 ± 0.02 |
| Stag_zymo V2 RDP | 0.31 ± 0.00 | 0.40 ± 0.00 | 0.44 ± 0.02 |
| Stag_atcc V3 Greengenes | 0.25 ± 0.01 | 0.36 ± 0.01 | 0.26 ± 0.01 |
| Stag_atcc V3 Silva | 0.10 ± 0.01 | 0.17 ± 0.01 | 0.12 ± 0.02 |
| Stag_atcc V3 RDP | 0.10 ± 0.01 | 0.17 ± 0.01 | 0.12 ± 0.02 |
| Stag_bei V3 Greengenes | 0.32 ± 0.03 | 0.42 ± 0.07 | 0.37 ± 0.12 |
| Stag_bei V3 Silva | 0.18 ± 0.10 | 0.24 ± 0.16 | 0.24 ± 0.19 |
| Stag_bei V3 RDP | 0.18 ± 0.10 | 0.24 ± 0.16 | 0.24 ± 0.19 |
| Stag_zymo V3 Greengenes | 0.39 ± 0.01 | 0.48 ± 0.01 | 0.49 ± 0.00 |
| Stag_zymo V3 Silva | 0.27 ± 0.01 | 0.38 ± 0.02 | 0.39 ± 0.00 |
| Stag_zymo V3 RDP | 0.42 ± 0.00 | 0.56 ± 0.00 | 0.59 ± 0.01 |
| Stag_atcc V4 Greengenes | 0.65 ± 0.05 | 0.49 ± 0.02 | 0.61 ± 0.04 |
| Stag_atcc V4 Silva | 0.65 ± 0.05 | 0.46 ± 0.03 | 0.59 ± 0.05 |
| Stag_atcc V4 RDP | 0.86 ± 0.05 | 0.68 ± 0.02 | 0.79 ± 0.05 |
| Stag_bei V4 Greengenes | 0.33 ± 0.02 | 0.42 ± 0.07 | 0.40 ± 0.10 |
| Stag_bei V4 Silva | 0.24 ± 0.08 | 0.28 ± 0.15 | 0.30 ± 0.17 |
| Stag_bei V4 RDP | 0.47 ± 0.07 | 0.53 ± 0.08 | 0.54 ± 0.13 |
| Stag_zymo V4 Greengenes | 0.47 ± 0.03 | 0.53 ± 0.00 | 0.50 ± 0.04 |
| Stag_zymo V4 Silva | 0.20 ± 0.03 | 0.21 ± 0.04 | 0.25 ± 0.06 |
| Stag_zymo V4 RDP | 0.39 ± 0.02 | 0.48 ± 0.01 | 0.45 ± 0.03 |
| Stag_atcc V67 Greengenes | 0.39 ± 0.01 | 0.52 ± 0.00 | 0.47 ± 0.01 |
| Stag_atcc V67 Silva | 0.28 ± 0.01 | 0.38 ± 0.00 | 0.28 ± 0.01 |
| Stag_atcc V67 RDP | 0.25 ± 0.01 | 0.39 ± 0.00 | 0.29 ± 0.01 |
| Stag_bei V67 Greengenes | 0.49 ± 0.05 | 0.58 ± 0.03 | 0.55 ± 0.09 |
| Stag_bei V67 Silva | 0.35 ± 0.06 | 0.45 ± 0.08 | 0.37 ± 0.17 |
| Stag_bei V67 RDP | 0.32 ± 0.06 | 0.47 ± 0.08 | 0.39 ± 0.18 |
| Stag_zymo V67 Greengenes | 0.46 ± 0.01 | 0.56 ± 0.01 | 0.58 ± 0.01 |
| Stag_zymo V67 Silva | 0.42 ± 0.00 | 0.49 ± 0.01 | 0.52 ± 0.01 |
| Stag_zymo V67 RDP | 0.54 ± 0.01 | 0.57 ± 0.01 | 0.62 ± 0.00 |
| Stag_atcc V8 Greengenes | 0.43 ± 0.04 | 0.49 ± 0.03 | 0.47 ± 0.05 |
| Stag_atcc V8 Silva | 0.36 ± 0.06 | 0.36 ± 0.08 | 0.33 ± 0.11 |
| Stag_atcc V8 RDP | 0.94 ± 0.06 | 0.77 ± 0.03 | 0.85 ± 0.06 |
| Stag_bei V8 Greengenes | 0.43 ± 0.02 | 0.51 ± 0.05 | 0.50 ± 0.09 |
| Stag_bei V8 Silva | 0.28 ± 0.06 | 0.31 ± 0.12 | 0.32 ± 0.16 |
| Stag_bei V8 RDP | 0.90 ± 0.08 | 0.74 ± 0.03 | 0.82 ± 0.08 |
| Stag_zymo V8 Greengenes | 0.52 ± 0.02 | 0.61 ± 0.02 | 0.72 ± 0.02 |
| Stag_zymo V8 Silva | 0.42 ± 0.01 | 0.46 ± 0.01 | 0.56 ± 0.00 |
| Stag_zymo V8 RDP | 1.07 ± 0.00 | 0.83 ± 0.00 | 1.00 ± 0.00 |
| Stag_atcc V9 Greengenes | 1.06 ± 0.00 | 0.81 ± 0.00 | 0.99 ± 0.00 |
| Stag_atcc V9 Silva | 1.06 ± 0.00 | 0.81 ± 0.00 | 0.99 ± 0.00 |
| Stag_atcc V9 RDP | 1.06 ± 0.00 | 0.81 ± 0.00 | 0.99 ± 0.00 |
| Stag_bei V9 Greengenes | 0.98 ± 0.21 | 0.79 ± 0.07 | 0.95 ± 0.09 |
| Stag_bei V9 Silva | 0.98 ± 0.21 | 0.79 ± 0.07 | 0.95 ± 0.09 |
| Stag_bei V9 RDP | 1.00 ± 0.19 | 0.80 ± 0.05 | 0.96 ± 0.06 |
| Stag_zymo V9 Greengenes | 1.07 ± 0.00 | 0.83 ± 0.00 | 1.00 ± 0.00 |
| Stag_zymo V9 Silva | 1.07 ± 0.00 | 0.83 ± 0.00 | 1.00 ± 0.00 |
| Stag_zymo V9 RDP | 1.07 ± 0.00 | 0.83 ± 0.00 | 1.00 ± 0.00 |

Even mock samples n = 21 (atcc_even n= 18 samples; bei_even n= 3 samples). Staggered mock samples n = 20 (atcc_stag n= 14 samples; bei_stag n= 4 samples; zymo_stag n= 2 samples). Euclidean, Jensen-Shannon, and Bray-Curtis Dissimilarity scores range between 0-1 where a score of 0 indicates zero dissimilarity between expected and actual mock bacterial abundance (or that expected and actual abundance are identical) and a score of 1 indicates complete dissimilarity between actual and expected abundances.
