# Supplementary material for: Multi-amplicon microbiome data analysis pipelines for mixed orientation sequences using QIIME2: Assessing reference database, variable region and pre-processing bias in classification of mock bacterial community samples
Source: PLoS One. 2023 Jan 13;18(1):e0280293. doi: 10.1371/journal.pone.0280293 (PMC9838852; doi:10.1371/journal.pone.0280293)
Supplement: S8 Table — Even mock samples atcc_even n = 18. n/a = Bacteria listed was not in the specified mock community. Values (mean or standard deviation) were rounded to two decimal places, and values < 0.005 were rounded to 0.0 (not true zero in every case). Taxon-specific agreement was defined as the observed/expected ratio and calculated as the observed relative abundance (%) / expected relative abundance (%) for each genus. A value of 1 indicates perfect agreement, a value under 0–0.999 indicates the actual relative abundance (%) is less than expected, and a value over 1 indicates the actual relative abundance (%) is higher than expected in the mock community for that individual taxon. Non-parametric tests were run to determine precision metric differences between V region (Kruskal-Wallis), reference databases (Kruskal-Wallis), and bioinformatics workflows (Wilcoxon Rank Sum), respectively, for each individual genus. (DOCX) [file pone.0280293.s013.docx]

**Supplemental Table 8: Taxon-Specific Metrics by Mock Type**

**Evenly-Spaced ATCC Mock Bacterial Community Samples V2, V3, V4**

| **Genus (Expected Abundance %)** | **Even ATCC V2 GG** | **Even ATCC V2 Silva** | **Even ATCC V2 RDP** | **Even ATCC V3 GG** | **Even ATCC V3 Silva** | **Even ATCC V3 RDP** | **Even ATCC V4 GG** | **Even ATCC V4 Silva** | **Even ATCC V4 RDP** |
| --- | --- | --- | --- | --- | --- | --- | --- | --- | --- |
| **CutPrimers** | | | | | | | | | |
| Acinetobacter (5%) | 1.82 ± 0.54 | 1.74 ± 0.36 | 1.95 ± 0.42 | 1.51 ± 0.36 | 1.51 ± 0.24 | 1.52 ± 0.24 | 1.85 ± 0.43 | 1.91 ± 0.25 | 2.41 ± 0.31 |
| Actinomyces (5%) | 0.20 ± 0.06 | 0.19 ± 0.05 | 0.0 ± 0.0 | 0.01 ± 0.00 | 0.01 ± 0.0 | 0.0 ± 0.0 | 0.17 ± 0.06 | 0.18 ± 0.05 | 0.0 ± 0.0 |
| Bacillus (5%) | 1.54 ± 1.57 | 1.41 ± 1.32 | 1.57 ± 1.46 | 2.55 ± 1.49 | 2.52 ± 1.23 | 2.52 ± 1.23 | 2.15 ± 1.17 | 2.17 ± 0.94 | 0.0 ± 0.0 |
| Bacteroides (5%) | 3.12 ± 1.42 | 3.12 ± 1.48 | 3.47 ± 1.62 | 2.39 ± 0.98 | 2.50 ± 1.07 | 2.50 ± 1.08 | 2.57 ± 1.02 | 2.78 ± 1.19 | 3.50 ± 1.50 |
| Bifidobacterium (5%) | 0.61 ± 0.29 | 0.62 ± 0.30 | 0.0 ± 0.0 | 0.01 ± 0.01 | 0.01 ± 0.01 | 0.0 ± 0.0 | 0.47 ± 0.21 | 0.51 ± 0.24 | 0.0 ± 0.0 |
| Clostridium (5%) | 1.23 ± 0.54 | 0.0 ± 0.0 | 0.0 ± 0.0 | 1.81 ± 0.80 | 0.0 ± 0.0 | 0.0 ± 0.0 | 1.89 ± 0.87 | 0.0 ± 0.0 | 0.0 ± 0.0 |
| Cutibacterium/ Propionibacterium (5%) | 0.18 ± 0.08 | 0.18 ± 0.09 | 0.0 ± 0.0 | 0.01 ± 0.00 | 0.01 ± 0.0 | 0.0 ± 0.0 | 0.01 ± 0.01 | 0.01 ± 0.01 | 0.0 ± 0.0 |
| Deinococcus (5%) | 2.19 ± 0.81 | 2.13 ± 0.70 | 2.37 ± 0.79 | 0.0 ± 0.0 | 0.0 ± 0.0 | 0.0 ± 0.0 | 0.13 ± 0.11 | 0.14 ± 0.09 | 0.17 ± 0.11 |
| Enterococcus (5%) | 0.81 ± 0.66 | 0.60 ± 0.59 | 0.67 ± 0.67 | 0.97 ± 0.42 | 0.96 ± 0.32 | 0.96 ± 0.32 | 0.61 ± 0.39 | 0.61 ± 0.31 | 0.78 ± 0.42 |
| Escherichia-Shigella (5%) | 0.0 ± 0.0 | 1.75 ± 0.83 | 0.87 ± 0.38 | 0.0 ± 0.0 | 1.51 ± 0.59 | 1.51 ± 0.59 | 0.0 ± 0.0 | 1.06 ± 0.80 | 1.35 ± 1.01 |
| Helicobacter (5%) | 1.70 ± 0.85 | 1.70 ± 0.87 | 1.89 ± 0.95 | 0.45 ± 0.13 | 0.47 ± 0.15 | 0.47 ± 0.15 | 0.69 ± 0.25 | 0.74 ± 0.30 | 0.93 ± 0.37 |
| Lactobacillus (5%) | 0.94 ± 0.31 | 0.93 ± 0.33 | 1.04 ± 0.38 | 1.56 ± 0.64 | 1.63 ± 0.70 | 1.64 ± 0.70 | 1.09 ± 0.32 | 1.17 ± 0.40 | 1.48 ± 0.49 |
| Listeria (0%) | n/a | n/a | n/a | n/a | n/a | n/a | n/a | n/a | n/a |
| Neisseria (5%) | 0.99 ± 0.09 | 0.96 ± 0.09 | 1.07 ± 0.10 | 0.84 ± 0.09 | 0.86 ± 0.14 | 0.86 ± 0.14 | 1.22 ± 0.07 | 1.28 ± 0.13 | 1.62 ± 0.17 |
| Porphyromonas (5%) | 0.69 ± 0.33 | 0.70 ± 0.34 | 0.77 ± 0.37 | 0.86 ± 0.24 | 0.89 ± 0.28 | 0.89 ± 0.28 | 1.42 ± 0.47 | 1.53 ± 0.56 | 1.92 ± 0.70 |
| Pseudomonas (5%) | 1.71 ± 0.86 | 1.61 ± 0.64 | 1.80 ± 0.73 | 1.32 ± 0.52 | 1.31 ± 0.39 | 1.31 ± 0.39 | 0.74 ± 0.48 | 0.74 ± 0.38 | 0.93 ± 0.47 |
| Rhodobacter (5%) | 0.70 ± 0.32 | 0.66 ± 0.24 | 0.74 ± 0.28 | 0.80 ± 0.38 | 0.80 ± 0.30 | 0.80 ± 0.30 | 0.66 ± 0.28 | 0.68 ± 0.21 | 0.85 ± 0.24 |
| Salmonella (0%) | n/a | n/a | n/a | n/a | n/a | n/a | n/a | n/a | n/a |
| Staphylococcus (10%) | 0.63 ± 0.25 | 0.63 ± 0.26 | 0.65 ± 0.30 | 0.95 ± 0.35 | 1.00 ± 0.39 | 1.00 ± 0.39 | 0.65 ± 0.25 | 0.70 ± 0.28 | 0.07 ± 0.03 |
| Streptococcus (10%) | 0.23 ± 0.13 | 0.22 ± 0.10 | 0.24 ± 0.12 | 1.50 ± 0.37 | 1.51 ± 0.27 | 1.51 ± 0.27 | 1.51 ± 0.51 | 1.55 ± 0.36 | 1.96 ± 0.45 |

**Evenly-Spaced ATCC Mock Bacterial Community Samples V6-7, V8, V9**

| **Genus (Expected Abundance %)** | **Even ATCC V6-7 GG** | **Even ATCC V6-7 Silva** | **Even ATCC V6-7 RDP** | **Even ATCC V8 GG** | **Even ATCC V8 Silva** | **Even ATCC V8 RDP** | **Even ATCC V9 GG** | **Even ATCC V9 Silva** | **Even ATCC V9 RDP** |
| --- | --- | --- | --- | --- | --- | --- | --- | --- | --- |
| **CutPrimers** | | | | | | | | | |
| Acinetobacter (5%) | 0.02 ± 0.02 | 0.02 ± 0.01 | 0.02 ± 0.01 | 2.97 ± 0.25 | 2.66 ± 0.21 | 0.0 ± 0.0 | 19.70 ± 0.16 | 19.69 ± 0.16 | 19.74 ± 0.14 |
| Actinomyces (5%) | 0.15 ± 0.06 | 0.15 ± 0.03 | 0.0 ± 0.0 | 0.34 ± 0.14 | 0.31 ± 0.13 | 0.0 ± 0.0 | 0.0 ± 0.0 | 0.0 ± 0.0 | 0.0 ± 0.0 |
| Bacillus (5%) | 0.0 ± 0.0 | 2.47 ± 1.58 | 2.55 ± 1.74 | 0.14 ± 0.14 | 0.13 ± 0.11 | 0.0 ± 0.0 | 0.01 ± 0.01 | 0.02 ± 0.02 | 0.0 ± 0.0 |
| Bacteroides (5%) | 5.33 ± 1.02 | 5.89 ± 2.12 | 5.89 ± 2.01 | 0.0 ± 0.0 | 0.0 ± 0.0 | 0.0 ± 0.0 | 0.0 ± 0.0 | 0.0 ± 0.0 | 0.0 ± 0.0 |
| Bifidobacterium (5%) | 0.25 ± 0.10 | 0.28 ± 0.14 | 0.0 ± 0.0 | 0.68 ± 0.37 | 0.63 ± 0.35 | 0.0 ± 0.0 | 0.0 ± 0.0 | 0.0 ± 0.0 | 0.0 ± 0.0 |
| Clostridium (5%) | 4.12 ± 1.70 | 0.0 ± 0.0 | 0.0 ± 0.0 | 0.01 ± 0.01 | 0.0 ± 0.0 | 0.0 ± 0.0 | 0.0 ± 0.0 | 0.0 ± 0.0 | 0.0 ± 0.0 |
| Cutibacterium/  Propionibacterium (5%) | 0.30 ± 0.12 | 0.34 ± 0.16 | 0.0 ± 0.0 | 1.30 ± 0.62 | 1.19 ± 0.58 | 0.0 ± 0.0 | 0.0 ± 0.0 | 0.0 ± 0.0 | 0.0 ± 0.0 |
| Deinococcus (5%) | 1.04 ± 0.60 | 1.01 ± 0.28 | 1.02 ± 0.31 | 1.58 ± 0.40 | 1.43 ± 0.37 | 6.19 ± 2.60 | 0.04 ± 0.03 | 0.04 ± 0.03 | 0.04 ± 0.03 |
| Enterococcus (5%) | 1.03 ± 1.02 | 0.0 ± 0.0 | 0.0 ± 0.0 | 1.01 ± 0.36 | 0.89 ± 0.27 | 0.0 ± 0.0 | 0.0 ± 0.0 | 0.0 ± 0.0 | 0.0 ± 0.0 |
| Escherichia-Shigella (5%) | 0.0 ± 0.0 | 2.62 ± 1.16 | 2.71 ± 1.37 | 0.0 ± 0.0 | 2.44 ± 0.70 | 0.0 ± 0.0 | 0.0 ± 0.0 | 0.0 ± 0.0 | 0.0 ± 0.0 |
| Helicobacter (5%) | 0.46 ± 0.10 | 0.50 ± 0.18 | 0.50 ± 0.17 | 0.05 ± 0.02 | 0.05 ± 0.02 | 2.37 ± 6.22 | 0.0 ± 0.0 | 0.0 ± 0.0 | 0.0 ± 0.0 |
| Lactobacillus (5%) | 1.39 ± 0.21 | 0.0 ± 0.0 | 1.49 ± 0.41 | 0.0 ± 0.0 | 0.0 ± 0.0 | 0.0 ± 0.0 | 0.0 ± 0.0 | 0.0 ± 0.0 | 0.0 ± 0.0 |
| Listeria (0%) | n/a | n/a | n/a | n/a | n/a | n/a | n/a | n/a | n/a |
| Neisseria (5%) | 0.0 ± 0.0 | 0.0 ± 0.0 | 0.0 ± 0.0 | 2.04 ± 0.35 | 1.84 ± 0.39 | 0.0 ± 0.0 | 0.0 ± 0.0 | 0.0 ± 0.0 | 0.0 ± 0.0 |
| Porphyromonas (5%) | 1.51 ± 0.29 | 1.55 ± 0.32 | 1.57 ± 0.29 | 0.0 ± 0.0 | 0.0 ± 0.0 | 0.0 ± 0.0 | 0.0 ± 0.0 | 0.0 ± 0.0 | 0.0 ± 0.0 |
| Pseudomonas (5%) | 0.02 ± 0.08 | 0.82 ± 0.74 | 0.01 ± 0.04 | 1.95 ± 0.59 | 1.73 ± 0.43 | 0.0 ± 0.0 | 0.02 ± 0.01 | 0.02 ± 0.01 | 0.02 ± 0.01 |
| Rhodobacter (5%) | 0.0 ± 0.0 | 0.0 ± 0.0 | 0.0 ± 0.0 | 2.96 ± 0.50 | 2.29 ± 0.91 | 11.44 ± 4.46 | 0.16 ± 0.09 | 0.16 ± 0.09 | 0.16 ± 0.09 |
| Salmonella (0%) | n/a | n/a | n/a | n/a | n/a | n/a | n/a | n/a | n/a |
| Staphylococcus (10%) | 0.89 ± 0.24 | 0.98 ± 0.36 | 0.90 ± 0.33 | 0.14 ± 0.05 | 0.12 ± 0.05 | 0.0 ± 0.0 | 0.01 ± 0.01 | 0.01 ± 0.01 | 0.01 ± 0.01 |
| Streptococcus (10%) | 1.30 ± 0.77 | 1.19 ± 0.23 | 1.22 ± 0.30 | 2.35 ± 0.32 | 2.09 ± 0.23 | 0.0 ± 0.0 | 0.02 ± 0.01 | 0.02 ± 0.01 | 0.01 ± 0.01 |

Even mock samples atcc_even n= 18. n/a = Bacteria listed was not in the specified mock community. Values (mean or standard deviation) were rounded to two decimal places, and values < 0.005 were rounded to 0.0 (not true zero in every case). Taxon-specific agreement was defined as the observed/expected ratio and calculated as the observed relative abundance (%) / expected relative abundance (%) for each genus. A value of 1 indicates perfect agreement, a value under 0-0.999 indicates the actual relative abundance (%) is less than expected, and a value over 1 indicates the actual relative abundance (%) is higher than expected in the mock community for that individual taxon. Non-parametric tests were run to determine precision metric differences between V region (Kruskal-Wallis), reference databases (Kruskal-Wallis), and bioinformatics workflows (Wilcoxon Rank Sum), respectively, for each individual genus.
