# Supplementary material for: Multi-amplicon microbiome data analysis pipelines for mixed orientation sequences using QIIME2: Assessing reference database, variable region and pre-processing bias in classification of mock bacterial community samples
Source: PLoS One. 2023 Jan 13;18(1):e0280293. doi: 10.1371/journal.pone.0280293 (PMC9838852; doi:10.1371/journal.pone.0280293)
Supplement: S9 Table — Even mock samples bei_even n = 3. n/a = Bacteria listed was not in the specified mock community. Values (mean or standard deviation) were rounded to two decimal places, and values < 0.005 were rounded to 0.0 (not true zero in every case). Taxon-specific agreement was defined as the observed/expected ratio and calculated as the observed relative abundance (%) / expected relative abundance (%) for each genus. A value of 1 indicates perfect agreement, a value under 0–0.999 indicates the actual relative abundance (%) is less than expected, and a value over 1 indicates the actual relative abundance (%) is higher than expected in the mock community for that individual taxon. Non-parametric tests were run to determine precision metric differences between V region (Kruskal-Wallis), reference databases (Kruskal-Wallis), and bioinformatics workflows (Wilcoxon Rank Sum), respectively, for each individual genus. (DOCX) [file pone.0280293.s014.docx]

**Supplemental Table 9:** **Taxon-Specific Metrics by Mock Type**

**Evenly-Spaced BEI Mock Bacterial Community Samples V2, V3, V4**

| **Genus (Expected Abundance %)** | **Even BEI V2 GG** | **Even BEI V2 Silva** | **Even BEI V2 RDP** | **Even BEI V3 GG** | **Even BEI V3 Silva** | **Even BEI V3 RDP** | **Even BEI V4 GG** | **Even BEI V4 Silva** | **Even BEI V4 RDP** |
| --- | --- | --- | --- | --- | --- | --- | --- | --- | --- |
| **CutPrimers** | | | | | | | | | |
| Acinetobacter (5%) | 1.17 ± 0.86 | 1.17 ± 0.91 | 1.24 ± 0.94 | 1.31 ± 0.08 | 1.38 ± 0.10 | 1.39 ± 0.10 | 0.74 ± 0.60 | 0.76 ± 0.63 | 1.12 ± 0.92 |
| Actinomyces (5%) | 0.11 ± 0.10 | 0.11 ± 0.10 | 0.0 ± 0.0 | 0.04 ± 0.00 | 0.04 ± 0.00 | 0.0 ± 0.0 | 0.26 ± 0.23 | 0.27 ± 0.23 | 0.0 ± 0.0 |
| Bacillus (5%) | 0.85 ± 0.38 | 0.81 ± 0.46 | 0.88 ± 0.45 | 1.32 ± 0.02 | 1.36 ± 0.03 | 1.37 ± 0.03 | 1.22 ± 0.53 | 1.21 ± 0.63 | 0.0 ± 0.0 |
| Bacteroides (5%) | 0.98 ± 0.78 | 0.98 ± 0.80 | 1.04 ± 0.84 | 1.54 ± 0.14 | 1.59 ± 0.14 | 1.60 ± 0.14 | 1.30 ± 1.12 | 1.34 ± 1.15 | 1.97 ± 1.69 |
| Bifidobacterium (0%) | n/a | n/a | n/a | n/a | n/a | n/a | n/a | n/a | n/a |
| Clostridium (5%) | 1.31 ± 0.21 | 0.0 ± 0.0 | 0.0 ± 0.0 | 1.44 ± 0.08 | 0.0 ± 0.0 | 0.0 ± 0.0 | 1.34 ± 0.84 | 0.0 ± 0.0 | 0.0 ± 0.0 |
| Cutibacterium/  Propionibacterium (5%) | 0.18 ± 0.11 | 0.17 ± 0.12 | 0.0 ± 0.0 | 0.04 ± 0.00 | 0.04 ± 0.00 | 0.0 ± 0.0 | 0.15 ± 0.13 | 0.16 ± 0.13 | 0.0 ± 0.0 |
| Deinococcus (5%) | 0.94 ± 0.76 | 0.95 ± 0.78 | 1.00 ± 0.82 | 0.0 ± 0.0 | 0.0 ± 0.0 | 0.0 ± 0.0 | 0.19 ± 0.16 | 0.19 ± 0.17 | 0.29 ± 0.25 |
| Enterococcus (5%) | 0.68 ± 0.59 | 0.68 ± 0.60 | 0.72 ± 0.63 | 1.05 ± 0.08 | 1.08 ± 0.10 | 1.09 ± 0.10 | 0.52 ± 0.45 | 0.54 ± 0.46 | 0.79 ± 0.69 |
| Escherichia-Shigella (5%) | 0.0 ± 0.0 | 3.08 ± 3.40 | 1.36 ± 1.47 | 0.0 ± 0.0 | 0.87 ± 0.08 | 0.87 ± 0.08 | 0.0 ± 0.0 | 1.71 ± 2.05 | 2.39 ± 2.81 |
| Helicobacter (5%) | 1.00 ± 0.52 | 0.96 ± 0.59 | 1.04 ± 0.59 | 1.57 ± 0.01 | 1.62 ± 0.02 | 1.62 ± 0.02 | 1.14 ± 0.92 | 1.17 ± 0.96 | 1.71 ± 1.41 |
| Lactobacillus (5%) | 1.07 ± 0.84 | 1.08 ± 0.88 | 1.14 ± 0.91 | 1.29 ± 0.02 | 1.33 ± 0.00 | 1.33 ± 0.01 | 1.08 ± 0.88 | 1.11 ± 0.92 | 1.64 ± 1.36 |
| Listeria (5%) | 0.57 ± 0.47 | 0.58 ± 0.48 | 0.61 ± 0.50 | 1.32 ± 0.07 | 1.36 ± 0.06 | 1.36 ± 0.06 | 0.0 ± 0.0 | 0.50 ± 0.41 | 0.0 ± 0.0 |
| Neisseria (5%) | 1.06 ± 0.75 | 1.05 ± 0.79 | 1.12 ± 0.82 | 0.89 ± 0.05 | 0.92 ± 0.05 | 0.92 ± 0.05 | 0.69 ± 0.51 | 0.70 ± 0.54 | 1.03 ± 0.80 |
| Porphyromonas (0%) | n/a | n/a | n/a | n/a | n/a | n/a | n/a | n/a | n/a |
| Pseudomonas (5%) | 1.95 ± 0.82 | 1.67 ± 0.29 | 1.93 ± 0.60 | 1.13 ± 0.09 | 1.17 ± 0.09 | 1.17 ± 0.09 | 0.49 ± 0.08 | 0.48 ± 0.12 | 0.69 ± 0.18 |
| Rhodobacter (5%) | 1.17 ± 0.97 | 0.94 ± 0.56 | 1.12 ± 0.82 | 0.71 ± 0.00 | 0.73 ± 0.01 | 0.73 ± 0.01 | 1.34 ± 1.53 | 1.15 ± 1.18 | 1.61 ± 1.61 |
| Salmonella (0%) | n/a | n/a | n/a | n/a | n/a | n/a | n/a | n/a | n/a |
| Staphylococcus (10%) | 2.28 ± 1.93 | 1.83 ± 1.12 | 2.19 ± 1.63 | 1.37 ± 0.02 | 1.41 ± 0.03 | 1.42 ± 0.03 | 2.16 ± 0.82 | 2.00 ± 0.46 | 0.01 ± 0.02 |
| Streptococcus (15%) | 0.80 ± 0.19 | 0.70 ± 0.08 | 0.80 ± 0.13 | 1.19 ± 0.02 | 1.23 ± 0.03 | 1.23 ± 0.03 | 1.74 ± 1.07 | 1.57 ± 0.72 | 2.24 ± 0.94 |

**Evenly-Spaced BEI Mock Bacterial Community Samples V6-7, V8, V9**

| **Genus (Expected Abundance %)** | **Even BEI V6-7 GG** | **Even BEI V6-7 Silva** | **Even BEI V6-7 RDP** | **Even BEI V8 GG** | **Even BEI V8 Silva** | **Even BEI V8 RDP** | **Even BEI V9 GG** | **Even BEI V9 Silva** | **Even BEI V9 RDP** |
| --- | --- | --- | --- | --- | --- | --- | --- | --- | --- |
| **CutPrimers** | | | | | | | | | |
| Acinetobacter (5%) | 0.05 ± 0.01 | 0.06 ± 0.01 | 0.06 ± 0.01 | 1.62 ± 1.37 | 1.35 ± 1.13 | 0.0 ± 0.0 | 19.63 ± 0.11 | 19.69 ± 0.15 | 19.77 ± 0.32 |
| Actinomyces (5%) | 0.92 ± 0.08 | 1.18 ± 0.14 | 0.0 ± 0.0 | 0.32 ± 0.28 | 0.27 ± 0.23 | 0.0 ± 0.0 | 0.0 ± 0.0 | 0.0 ± 0.0 | 0.0 ± 0.0 |
| Bacillus (5%) | 0.0 ± 0.0 | 1.17 ± 0.01 | 1.20 ± 0.01 | 0.17 ± 0.13 | 0.15 ± 0.11 | 0.0 ± 0.0 | 0.15 ± 0.21 | 0.03 ± 0.06 | 0.0 ± 0.0 |
| Bacteroides (5%) | 2.39 ± 0.20 | 3.06 ± 0.16 | 3.14 ± 0.21 | 0.0 ± 0.0 | 0.0 ± 0.0 | 0.0 ± 0.0 | 0.0 ± 0.0 | 0.0 ± 0.0 | 0.0 ± 0.0 |
| Bifidobacterium (0%) | n/a | n/a | n/a | n/a | n/a | n/a | n/a | n/a | n/a |
| Clostridium (5%) | 3.39 ± 0.09 | 0.0 ± 0.0 | 0.0 ± 0.0 | 0.01 ± 0.01 | 0.0 ± 0.0 | 0.0 ± 0.0 | 0.0 ± 0.0 | 0.0 ± 0.0 | 0.0 ± 0.0 |
| Cutibacterium/  Propionibacterium (5%) | 1.22 ± 0.05 | 1.56 ± 0.02 | 0.0 ± 0.0 | 1.28 ± 1.00 | 1.06 ± 0.83 | 0.0 ± 0.0 | 0.0 ± 0.0 | 0.0 ± 0.0 | 0.0 ± 0.0 |
| Deinococcus (5%) | 0.64 ± 0.01 | 0.83 ± 0.05 | 0.85 ± 0.03 | 0.72 ± 0.63 | 0.60 ± 0.52 | 4.78 ± 4.13 | 0.0 ± 0.0 | 0.04 ± 0.03 | 0.0 ± 0.0 |
| Enterococcus (5%) | 0.74 ± 0.00 | 0.0 ± 0.0 | 0.0 ± 0.0 | 0.93 ± 0.81 | 0.77 ± 0.67 | 0.0 ± 0.0 | 0.12 ± 0.17 | 0.01 ± 0.05 | 0.12 ± 0.17 |
| Escherichia-Shigella (5%) | 0.0 ± 0.0 | 0.93 ± 0.17 | 0.96 ± 0.18 | 0.0 ± 0.0 | 1.61 ± 0.19 | 0.0 ± 0.0 | 0.0 ± 0.0 | 0.0 ± 0.0 | 0.0 ± 0.0 |
| Helicobacter (5%) | 1.54 ± 0.24 | 1.97 ± 0.24 | 2.03 ± 0.28 | 0.25 ± 0.21 | 0.21 ± 0.17 | 1.65 ± 1.42 | 0.0 ± 0.0 | 0.0 ± 0.0 | 0.0 ± 0.0 |
| Lactobacillus (5%) | 2.28 ± 0.16 | 0.0 ± 0.0 | 2.99 ± 0.26 | 0.0 ± 0.0 | 0.0 ± 0.0 | 0.0 ± 0.0 | 0.0 ± 0.0 | 0.0 ± 0.0 | 0.0 ± 0.0 |
| Listeria (5%) | 2.73 ± 0.19 | 3.50 ± 0.36 | 3.59 ± 0.32 | 0.0 ± 0.0 | 1.28 ± 1.08 | 0.0 ± 0.0 | 0.0 ± 0.0 | 0.0 ± 0.0 | 0.0 ± 0.0 |
| Neisseria (5%) | 0.0 ± 0.0 | 0.0 ± 0.0 | 0.0 ± 0.0 | 1.27 ± 0.99 | 1.06 ± 0.82 | 0.0 ± 0.0 | 0.0 ± 0.0 | 0.0 ± 0.0 | 0.0 ± 0.0 |
| Porphyromonas (0%) | n/a | n/a | n/a | n/a | n/a | n/a | n/a | n/a | n/a |
| Pseudomonas (5%) | 0.0 ± 0.0 | 0.47 ± 0.09 | 0.0 ± 0.0 | 1.39 ± 0.58 | 1.18 ± 0.45 | 0.0 ± 0.0 | 0.0 ± 0.0 | 0.15 ± 0.09 | 0.0 ± 0.0 |
| Rhodobacter (5%) | 0.0 ± 0.0 | 0.0 ± 0.0 | 0.0 ± 0.0 | 5.10 ± 6.13 | 4.55 ± 5.63 | 13.57 ± 5.55 | 0.05 ± 0.07 | 0.15 ± 0.09 | 0.05 ± 0.07 |
| Salmonella (0%) | n/a | n/a | n/a | n/a | n/a | n/a | n/a | n/a | n/a |
| Staphylococcus (10%) | 0.87 ± 0.02 | 1.12 ± 0.01 | 1.04 ± 0.01 | 0.39 ± 0.17 | 0.33 ± 0.13 | 0.0 ± 0.0 | 0.03 ± 0.04 | 0.01 ± 0.01 | 0.03 ± 0.04 |
| Streptococcus (15%) | 0.79 ± 0.00 | 1.01 ± 0.03 | 1.03 ± 0.01 | 2.05 ± 0.12 | 1.76 ± 0.16 | 0.0 ± 0.0 | 0.0 ± 0.0 | 0.02 ± 0.01 | 0.0 ± 0.0 |

Even mock samples bei_even n= 3. n/a = Bacteria listed was not in the specified mock community. Values (mean or standard deviation) were rounded to two decimal places, and values < 0.005 were rounded to 0.0 (not true zero in every case). Taxon-specific agreement was defined as the observed/expected ratio and calculated as the observed relative abundance (%) / expected relative abundance (%) for each genus. A value of 1 indicates perfect agreement, a value under 0-0.999 indicates the actual relative abundance (%) is less than expected, and a value over 1 indicates the actual relative abundance (%) is higher than expected in the mock community for that individual taxon. Non-parametric tests were run to determine precision metric differences between V region (Kruskal-Wallis), reference databases (Kruskal-Wallis), and bioinformatics workflows (Wilcoxon Rank Sum), respectively, for each individual genus.
