# Supplementary material for: Multi-amplicon microbiome data analysis pipelines for mixed orientation sequences using QIIME2: Assessing reference database, variable region and pre-processing bias in classification of mock bacterial community samples
Source: PLoS One. 2023 Jan 13;18(1):e0280293. doi: 10.1371/journal.pone.0280293 (PMC9838852; doi:10.1371/journal.pone.0280293)
Supplement: S10 Table — Staggered mock samples atcc_stag n = 14 samples. n/a = Bacteria listed was not in the specified mock community. Values (mean or standard deviation) were rounded to two decimal places, and values < 0.005 were rounded to 0.0 (not true zero in every case). Taxon-specific agreement was defined as the observed/expected ratio and calculated as the observed relative abundance (%) / expected relative abundance (%) for each genus. A value of 1 indicates perfect agreement, a value under 0–0.999 indicates the actual relative abundance (%) is less than expected, and a value over 1 indicates the actual relative abundance (%) is higher than expected in the mock community for that individual taxon. Non-parametric tests were run to determine precision metric differences between V region (Kruskal-Wallis), reference databases (Kruskal-Wallis), and bioinformatics workflows (Wilcoxon Rank Sum), respectively, for each individual genus. (DOCX) [file pone.0280293.s015.docx]

**Supplemental Table 10: Taxon-Specific Metrics by Mock Type**

**Staggered ATCC Mock Bacterial Community Samples V2, V3, V4**

| **Genus (Expected**  **Abundance %)** | **Stag ATCC V2 GG** | **Stag ATCC V2 Silva** | **Stag ATCC V2 RDP** | **Stag ATCC V3 GG** | **Stag ATCC V3 Silva** | **Stag ATCC V3 RDP** | **Stag ATCC V4 GG** | **Stag ATCC V4 Silva** | **Stag ATCC V4 RDP** |
| --- | --- | --- | --- | --- | --- | --- | --- | --- | --- |
| **CutPrimers** | | | | | | | | | |
| Acinetobacter (0.18%) | 0.54 ± 0.11 | 0.52 ± 0.11 | 0.74 ± 0.52 | 1.80 ± 0.14 | 1.58 ± 0.12 | 1.58 ± 0.12 | 0.36 ± 0.10 | 0.35 ± 0.09 | 1.70 ± 0.35 |
| Actinomyces (0.02%) | 0.04 ± 0.10 | 0.04 ± 0.10 | 0.0 ± 0.0 | 0.01 ± 0.02 | 0.00 ± 0.02 | 0.0 ± 0.0 | 0.03 ± 0.07 | 0.03 ± 0.07 | 0.0 ± 0.0 |
| Bacillus (1.8%) | 0.18 ± 0.02 | 0.17 ± 0.02 | 0.26 ± 0.22 | 1.99 ± 0.06 | 1.76 ± 0.06 | 1.76 ± 0.06 | 0.20 ± 0.06 | 0.19 ± 0.06 | 0.0 ± 0.0 |
| Bacteroides (0.02%) | 7.51 ± 1.77 | 7.24 ± 1.70 | 10.59 ± 8.23 | 2.96 ± 0.63 | 2.57 ± 0.44 | 2.57 ± 0.44 | 0.85 ± 0.40 | 0.84 ± 0.39 | 4.28 ± 2.17 |
| Bifidobacterium (0.02%) | 0.58 ± 0.53 | 0.56 ± 0.51 | 0.0 ± 0.0 | 0.00 ± 0.02 | 0.00 ± 0.01 | 0.0 ± 0.0 | 0.0 ± 0.0 | 0.0 ± 0.0 | 0.0 ± 0.0 |
| Clostridium (1.8%) | 0.41 ± 0.05 | 0.0 ± 0.0 | 0.0 ± 0.0 | 3.01 ± 0.14 | 0.0 ± 0.0 | 0.0 ± 0.0 | 0.44 ± 0.09 | 0.0 ± 0.0 | 0.0 ± 0.0 |
| Cutibacterium/  Propionibacterium (0.18%) | 0.11 ± 0.07 | 0.11 ± 0.07 | 0.0 ± 0.0 | 0.01 ± 0.01 | 0.01 ± 0.01 | 0.0 ± 0.0 | 0.0 ± 0.0 | 0.0 ± 0.0 | 0.0 ± 0.0 |
| Deinococcus (0.02%) | 3.67 ± 0.81 | 3.54 ± 0.78 | 5.16 ± 4.02 | 0.0 ± 0.0 | 0.0 ± 0.0 | 0.0 ± 0.0 | 0.0 ± 0.0 | 0.0 ± 0.0 | 0.0 ± 0.0 |
| Enterococcus (0.02%) | 0.0 ± 0.0 | 0.0 ± 0.0 | 0.0 ± 0.0 | 0.75 ± 0.28 | 0.67 ± 0.27 | 0.67 ± 0.27 | 0.0 ± 0.0 | 0.0 ± 0.0 | 0.0 ± 0.0 |
| Escherichia-Shigella (18%) | 0.0 ± 0.0 | 0.24 ± 0.02 | 0.17 ± 0.14 | 0.0 ± 0.0 | 0.91 ± 0.15 | 0.91 ± 0.15 | 0.0 ± 0.0 | 0.13 ± 0.04 | 0.63 ± 0.04 |
| Helicobacter (0.18%) | 3.15 ± 0.79 | 3.04 ± 0.76 | 4.19 ± 2.60 | 0.75 ± 0.10 | 0.66 ± 0.11 | 0.66 ± 0.11 | 0.16 ± 0.07 | 0.15 ± 0.07 | 0.73 ± 0.24 |
| Lactobacillus (0.18%) | 0.38 ± 0.12 | 0.37 ± 0.11 | 0.52 ± 0.37 | 2.71 ± 0.18 | 2.39 ± 0.22 | 2.39 ± 0.22 | 0.22 ± 0.09 | 0.21 ± 0.08 | 1.00 ± 0.19 |
| Listeria (0%) | n/a | n/a | n/a | n/a | n/a | n/a | n/a | n/a | n/a |
| Neisseria (0.18%) | 0.38 ± 0.11 | 0.37 ± 0.11 | 0.51 ± 0.30 | 0.99 ± 0.10 | 0.87 ± 0.08 | 0.87 ± 0.08 | 0.32 ± 0.13 | 0.32 ± 0.13 | 1.49 ± 0.36 |
| Porphyromonas (18%) | 1.07 ± 0.16 | 1.03 ± 0.15 | 1.44 ± 0.91 | 1.10 ± 0.05 | 0.97 ± 0.06 | 0.97 ± 0.06 | 0.51 ± 0.13 | 0.51 ± 0.12 | 2.41 ± 0.10 |
| Pseudomonas (1.8%) | 0.44 ± 0.06 | 0.42 ± 0.06 | 0.65 ± 0.57 | 1.25 ± 0.07 | 1.10 ± 0.07 | 1.10 ± 0.07 | 0.08 ± 0.02 | 0.08 ± 0.02 | 0.36 ± 0.05 |
| Rhodobacter (18%) | 0.18 ± 0.03 | 0.17 ± 0.03 | 0.26 ± 0.22 | 0.78 ± 0.03 | 0.69 ± 0.03 | 0.69 ± 0.03 | 0.09 ± 0.02 | 0.09 ± 0.02 | 0.42 ± 0.03 |
| Salmonella (0%) | n/a | n/a | n/a | n/a | n/a | n/a | n/a | n/a | n/a |
| Staphylococcus (19.8%) | 3.71 ± 0.15 | 3.58 ± 0.14 | 3.12 ± 1.32 | 1.42 ± 0.02 | 1.25 ± 0.05 | 1.25 ± 0.05 | 4.05 ± 0.23 | 3.99 ± 0.25 | 0.05 ± 0.05 |
| Streptococcus (19.8%) | 0.06 ± 0.01 | 0.06 ± 0.01 | 0.09 ± 0.06 | 1.29 ± 0.04 | 1.14 ± 0.05 | 1.14 ± 0.05 | 0.37 ± 0.08 | 0.37 ± 0.08 | 1.77 ± 0.08 |

**Staggered ATCC Mock Bacterial Community Samples V67, V8, V9**

| **Genus (Expected Abundance %)** | **Stag ATCC V67 GG** | **Stag ATCC V67 Silva** | **Stag ATCC V67 RDP** | **Stag ATCC V8 GG** | **Stag ATCC V8 Silva** | **Stag ATCC**  **V8 RDP** | **Stag ATCC**  **V9 GG** | **Stag ATCC**  **V9 Silva** | **Stag ATCC**  **V9 RDP** |
| --- | --- | --- | --- | --- | --- | --- | --- | --- | --- |
| **CutPrimers** | | | | | | | | | |
| Acinetobacter (0.18%) | 0.00 ± 0.01 | 0.00 ± 0.01 | 0.00 ± 0.01 | 1.74 ± 0.15 | 1.48 ± 0.20 | 0.0 ± 0.0 | 309.47 ± 13.18 | 307.35 ± 12.67 | 308.13 ± 13.65 |
| Actinomyces (0.02%) | 0.19 ± 0.15 | 0.16 ± 0.13 | 0.0 ± 0.0 | 0.19 ± 0.16 | 0.16 ± 0.15 | 0.0 ± 0.0 | 0.0 ± 0.0 | 0.0 ± 0.0 | 0.0 ± 0.0 |
| Bacillus (1.8%) | 0.0 ± 0.0 | 1.62 ± 0.13 | 1.63 ± 0.14 | 0.04 ± 0.02 | 0.04 ± 0.01 | 0.0 ± 0.0 | 0.07 ± 0.15 | 0.12 ± 0.19 | 0.0 ± 0.0 |
| Bacteroides (0.02%) | 7.67 ± 0.88 | 6.51 ± 0.68 | 6.57 ± 0.69 | 0.0 ± 0.0 | 0.0 ± 0.0 | 0.0 ± 0.0 | 0.0 ± 0.0 | 0.0 ± 0.0 | 0.0 ± 0.0 |
| Bifidobacterium (0.02%) | 0.27 ± 0.23 | 0.23 ± 0.19 | 0.0 ± 0.0 | 0.33 ± 0.30 | 0.29 ± 0.28 | 0.0 ± 0.0 | 0.0 ± 0.0 | 0.0 ± 0.0 | 0.0 ± 0.0 |
| Clostridium (1.8%) | 7.98 ± 0.16 | 0.0 ± 0.0 | 0.0 ± 0.0 | 0.0 ± 0.0 | 0.0 ± 0.0 | 0.0 ± 0.0 | 0.0 ± 0.0 | 0.0 ± 0.0 | 0.0 ± 0.0 |
| Cutibacterium/  Propionibacterium (0.18%) | 0.64 ± 0.09 | 0.54 ± 0.08 | 0.0 ± 0.0 | 0.91 ± 0.10 | 0.77 ± 0.12 | 0.0 ± 0.0 | 0.0 ± 0.0 | 0.0 ± 0.0 | 0.0 ± 0.0 |
| Deinococcus (0.02%) | 0.57 ± 0.28 | 0.49 ± 0.24 | 0.49 ± 0.24 | 0.61 ± 0.26 | 0.53 ± 0.25 | 2.27 ± 1.41 | 0.0 ± 0.0 | 0.0 ± 0.0 | 0.0 ± 0.0 |
| Enterococcus (0.02%) | 0.46 ± 0.39 | 0.0 ± 0.0 | 0.0 ± 0.0 | 0.15 ± 0.19 | 0.12 ± 0.16 | 0.0 ± 0.0 | 0.0 ± 0.0 | 0.0 ± 0.0 | 0.0 ± 0.0 |
| Escherichia-Shigella (18%) | 0.0 ± 0.0 | 1.32 ± 0.06 | 1.34 ± 0.06 | 0.0 ± 0.0 | 1.04 ± 0.24 | 0.0 ± 0.0 | 0.0 ± 0.0 | 0.0 ± 0.0 | 0.01 ± 0.02 |
| Helicobacter (0.18%) | 0.71 ± 0.07 | 0.60 ± 0.05 | 0.61 ± 0.05 | 0.03 ± 0.02 | 0.03 ± 0.02 | 79.49 ± 201.69 | 0.0 ± 0.0 | 0.0 ± 0.0 | 0.0 ± 0.0 |
| Lactobacillus (0.18%) | 1.84 ± 0.18 | 0.0 ± 0.0 | 1.57 ± 0.15 | 0.0 ± 0.0 | 0.0 ± 0.0 | 0.0 ± 0.0 | 0.0 ± 0.0 | 0.0 ± 0.0 | 0.0 ± 0.0 |
| Listeria (0%) | n/a | n/a | n/a | n/a | n/a | n/a | n/a | n/a | n/a |
| Neisseria (0.18%) | 0.0 ± 0.0 | 0.0 ± 0.0 | 0.0 ± 0.0 | 1.36 ± 0.14 | 1.16 ± 0.17 | 0.0 ± 0.0 | 0.0 ± 0.0 | 0.0 ± 0.0 | 0.0 ± 0.0 |
| Porphyromonas (18%) | 2.05 ± 0.10 | 1.74 ± 0.09 | 1.76 ± 0.09 | 0.0 ± 0.0 | 0.0 ± 0.0 | 0.0 ± 0.0 | 0.02 ± 0.03 | 0.06 ± 0.02 | 0.06 ± 0.02 |
| Pseudomonas (1.8%) | 0.0 ± 0.0 | 0.42 ± 0.05 | 0.0 ± 0.0 | 0.84 ± 0.09 | 0.72 ± 0.12 | 0.0 ± 0.0 | 0.31 ± 0.23 | 0.31 ± 0.23 | 0.27 ± 0.26 |
| Rhodobacter (18%) | 0.0 ± 0.0 | 0.0 ± 0.0 | 0.0 ± 0.0 | 1.20 ± 0.13 | 0.82 ± 0.35 | 4.76 ± 2.02 | 1.83 ± 0.15 | 1.81 ± 0.15 | 1.82 ± 0.15 |
| Salmonella (0%) | n/a | n/a | n/a | n/a | n/a | n/a | n/a | n/a | n/a |
| Staphylococcus (19.8%) | 1.38 ± 0.05 | 1.17 ± 0.05 | 1.17 ± 0.05 | 2.99 ± 0.22 | 2.56 ± 0.37 | 0.0 ± 0.0 | 0.30 ± 0.08 | 0.30 ± 0.08 | 0.30 ± 0.08 |
| Streptococcus (19.8%) | 1.05 ± 0.06 | 0.89 ± 0.05 | 0.90 ± 0.05 | 0.85 ± 0.31 | 0.71 ± 0.25 | 0.0 ± 0.0 | 0.22 ± 0.03 | 0.22 ± 0.03 | 0.21 ± 0.03 |

Staggered mock samples atcc_stag n= 14 samples. n/a = Bacteria listed was not in the specified mock community. Values (mean or standard deviation) were rounded to two decimal places, and values < 0.005 were rounded to 0.0 (not true zero in every case). Taxon-specific agreement was defined as the observed/expected ratio and calculated as the observed relative abundance (%) / expected relative abundance (%) for each genus. A value of 1 indicates perfect agreement, a value under 0-0.999 indicates the actual relative abundance (%) is less than expected, and a value over 1 indicates the actual relative abundance (%) is higher than expected in the mock community for that individual taxon. Non-parametric tests were run to determine precision metric differences between V region (Kruskal-Wallis), reference databases (Kruskal-Wallis), and bioinformatics workflows (Wilcoxon Rank Sum), respectively, for each individual genus.
