# Supplementary material for: Multi-amplicon microbiome data analysis pipelines for mixed orientation sequences using QIIME2: Assessing reference database, variable region and pre-processing bias in classification of mock bacterial community samples
Source: PLoS One. 2023 Jan 13;18(1):e0280293. doi: 10.1371/journal.pone.0280293 (PMC9838852; doi:10.1371/journal.pone.0280293)
Supplement: S11 Table — Staggered mock samples bei_stag n = 4 samples. n/a = Bacteria listed was not in the specified mock community. Values (mean or standard deviation) were rounded to two decimal places, and values < 0.005 were rounded to 0.0 (not true zero in every case). Taxon-specific agreement was defined as the observed/expected ratio and calculated as the observed relative abundance (%) / expected relative abundance (%) for each genus. A value of 1 indicates perfect agreement, a value under 0–0.999 indicates the actual relative abundance (%) is less than expected, and a value over 1 indicates the actual relative abundance (%) is higher than expected in the mock community for that individual taxon. Non-parametric tests were run to determine precision metric differences between V region (Kruskal-Wallis), reference databases (Kruskal-Wallis), and bioinformatics workflows (Wilcoxon Rank Sum), respectively, for each individual genus. (DOCX) [file pone.0280293.s016.docx]

**Supplemental Table 11: Taxon-Specific Metrics by Mock Type**

**Staggered BEI Mock Bacterial Community Samples V2, V3, V4**

| **Genus (Expected Abundance %)** | **Stag BEI**  **V2 GG** | **Stag BEI**  **V2 Silva** | **Stag BEI V2 RDP** | **Stag BEI V3 GG** | **Stag BEI V3 Silva** | **Stag BEI V3 RDP** | **Stag BEI V4 GG** | **Stag BEI V4 Silva** | **Stag BEI V4 RDP** |
| --- | --- | --- | --- | --- | --- | --- | --- | --- | --- |
| **CutPrimers** | | | | | | | | | |
| Acinetobacter (0.22%) | 8.81 ± 13.82 | 8.69 ± 14.46 | 10.03 ± 16.65 | 7.48 ± 12.13 | 7.45 ± 12.55 | 7.46 ± 12.57 | 6.18 ± 10.38 | 5.98 ± 10.22 | 8.59 ± 14.45 |
| Actinomyces (0.02%) | 10.56 ± 21.12 | 10.87 ± 21.74 | 0.0 ± 0.0 | 1.09 ± 2.18 | 1.12 ± 2.23 | 0.0 ± 0.0 | 14.29 ± 28.09 | 13.99 ± 27.53 | 0.0 ± 0.0 |
| Bacillus (2.19%) | 2.09 ± 0.86 | 1.83 ± 1.10 | 2.12 ± 1.25 | 1.76 ± 0.78 | 1.60 ± 0.93 | 1.60 ± 0.93 | 1.95 ± 0.96 | 1.82 ± 1.02 | 0.0 ± 0.0 |
| Bacteroides (0.02%) | 115.63 ± 219.18 | 117.76 ± 226.42 | 135.76 ± 260.80 | 99.40 ± 196.48 | 101.54 ± 201.11 | 101.71 ± 201.45 | 111.73 ± 220.24 | 109.42 ±  215.87 | 155.71 ± 306.26 |
| Bifidobacterium (0%) | n/a | n/a | n/a | n/a | n/a | n/a | n/a | n/a | n/a |
| Clostridium (2.19%) | 2.11 ± 1.02 | 0.0 ± 0.0 | 0.0 ± 0.0 | 1.72 ± 1.00 | 0.0 ± 0.0 | 0.0 ± 0.0 | 1.89 ± 1.35 | 0.0 ± 0.0 | 0.0 ± 0.0 |
| Cutibacterium/  Propionibacterium (0.22%) | 1.86 ± 2.59 | 1.80 ± 2.73 | 0.0 ± 0.0 | 0.12 ± 0.15 | 0.12 ± 0.15 | 0.0 ± 0.0 | 0.52 ± 0.78 | 0.51 ± 0.77 | 0.0 ± 0.0 |
| Deinococcus (0.02%) | 72.88±129.55 | 73.31±134.42 | 84.59±154.78 | 0.0 ± 0.0 | 0.0 ± 0.0 | 0.0 ± 0.0 | 6.57±12.50 | 6.42±12.26 | 9.16 ± 17.38 |
| Enterococcus (0.02%) | 70.72±141.43 | 72.79±145.58 | 83.86±167.72 | 76.44±151.33 | 78.10±154.88 | 78.23±155.14 | 56.81±113.62 | 55.67±111.34 | 79.04±158.08 |
| Escherichia-Shigella (21.91%) | 0.0 ± 0.0 | 0.94 ± 0.48 | 0.49 ± 0.26 | 0.0 ± 0.0 | 0.72 ± 0.37 | 0.72 ± 0.37 | 0.0 ± 0.0 | 0.54 ± 0.33 | 0.82 ± 0.43 |
| Helicobacter (0.22%) | 12.05 ± 15.89 | 11.51 ± 16.78 | 13.33 ± 19.31 | 8.97 ± 13.63 | 8.84 ± 14.15 | 8.86 ± 14.18 | 7.92 ± 11.76 | 7.62 ± 11.62 | 11.07 ± 16.34 |
| Lactobacillus (0.22%) | 13.89 ± 23.18 | 13.85 ± 24.16 | 15.98 ± 27.82 | 5.69 ± 8.77 | 5.63 ± 9.09 | 5.63 ± 9.11 | 9.73 ± 15.51 | 9.39 ± 15.30 | 13.56 ± 21.58 |
| Listeria (0.22%) | 5.27 ± 8.99 | 5.29 ± 9.34 | 6.09 ± 10.76 | 7.99 ± 13.90 | 8.02 ± 14.32 | 8.04 ± 14.34 | 0.0 ± 0.0 | 7.38 ± 13.22 | 0.0 ± 0.0 |
| Neisseria (0.22%) | 7.70 ± 10.62 | 7.44 ± 11.24 | 8.60 ± 12.93 | 6.25 ± 10.42 | 6.24 ± 10.76 | 6.24 ± 10.78 | 7.96 ± 12.26 | 7.61 ± 12.12 | 10.90 ± 17.13 |
| Porphyromonas (0%) | n/a | n/a | n/a | n/a | n/a | n/a | n/a | n/a | n/a |
| Pseudomonas (2.19%) | 2.73 ± 1.52 | 2.21 ± 1.04 | 2.59 ± 1.27 | 1.77 ± 0.74 | 1.60 ± 0.86 | 1.60 ± 0.86 | 1.27 ± 0.75 | 1.15 ± 0.72 | 1.69 ± 0.93 |
| Rhodobacter (21.91%) | 0.70 ± 0.47 | 0.54 ± 0.34 | 0.63 ± 0.40 | 0.61 ± 0.33 | 0.51 ± 0.25 | 0.51 ± 0.25 | 0.49 ± 0.29 | 0.42 ± 0.22 | 0.65 ± 0.31 |
| Salmonella (0%) | n/a | n/a | n/a | n/a | n/a | n/a | n/a | n/a | n/a |
| Staphylococcus (24.1%) | 1.50 ± 0.60 | 1.20 ± 0.38 | 1.30 ± 0.64 | 1.24 ± 0.40 | 1.06 ± 0.29 | 1.07 ± 0.29 | 1.31 ± 0.52 | 1.18 ± 0.49 | 0.0 ± 0.0 |
| Streptococcus (24.102%) | 0.70 ± 0.43 | 0.57 ± 0.33 | 0.66 ± 0.37 | 1.39 ± 0.38 | 1.19 ± 0.23 | 1.19 ± 0.23 | 1.48 ± 0.38 | 1.32 ± 0.27 | 2.06 ± 0.58 |

**Staggered ATCC Mock Bacterial Community Samples V6-7, V8, V9**

| **Genus (Expected Abundance %)** | **Stag BEI**  **V6-7 GG** | **Stag BEI**  **V6-7 Silva** | **Stag BEI V6-7 RDP** | **Stag BEI V8 GG** | **Stag BEI V8 Silva** | **Stag BEI V8 RDP** | **Stag BEI V9 GG** | **Stag BEI**  **V9 Silva** | **Stag BEI V9 RDP** |
| --- | --- | --- | --- | --- | --- | --- | --- | --- | --- |
| **CutPrimers** | | | | | | | | | |
| Acinetobacter (0.22%) | 0.83 ± 1.66 | 0.95 ± 1.90 | 0.93 ± 1.85 | 13.71 ± 23.84 | 11.56 ± 20.53 | 0.0 ± 0.0 | 285.43 ± 94.00 | 285.43 ± 94.00 | 293.59 ± 93.89 |
| Actinomyces (0.02%) | 16.85 ± 29.20 | 18.68 ± 33.77 | 0.0 ± 0.0 | 33.86 ± 64.94 | 28.80 ± 55.71 | 0.0 ± 0.0 | 0.0 ± 0.0 | 0.0 ± 0.0 | 0.0 ± 0.0 |
| Bacillus (2.19%) | 0.0 ± 0.0 | 2.07 ± 1.22 | 2.08 ± 1.16 | 0.24 ± 0.21 | 0.19 ± 0.19 | 0.0 ± 0.0 | 0.52 ± 1.05 | 0.52 ± 1.05 | 0.0 ± 0.0 |
| Bacteroides (0.02%) | 165.66 ± 305.32 | 184.87 ± 352.22 | 181.22 ± 344.36 | 0.0 ± 0.0 | 0.0 ± 0.0 | 0.0 ± 0.0 | 0.0 ± 0.0 | 0.0 ± 0.0 | 0.0 ± 0.0 |
| Bifidobacterium (0%) | n/a | n/a | n/a | n/a | n/a | n/a | n/a | n/a | n/a |
| Clostridium (2.19%) | 5.07 ± 1.46 | 0.0 ± 0.0 | 0.0 ± 0.0 | 0.03 ± 0.04 | 0.0 ± 0.0 | 0.0 ± 0.0 | 0.0 ± 0.0 | 0.0 ± 0.0 | 0.0 ± 0.0 |
| Cutibacterium/ Propionibacterium (0%) | 4.10 ± 2.74 | 3.87 ± 3.68 | 0.0 ± 0.0 | 11.82 ± 19.04 | 9.86 ± 16.47 | 0.0 ± 0.0 | 0.0 ± 0.0 | 0.0 ± 0.0 | 0.0 ± 0.0 |
| Deinococcus (0.02%) | 60.96 ± 121.92 | 69.74 ± 139.47 | 68.23 ± 136.45 | 23.21 ± 44.78 | 19.85 ± 38.41 | 229.71 ± 454.25 | 65.16 ± 130.33 | 65.16 ± 130.33 | 72.97 ± 145.95 |
| Enterococcus (0.02%) | 72.79±145.57 | 0.0 ± 0.0 | 0.0 ± 0.0 | 52.66±103.17 | 44.98±88.39 | 0.0 ± 0.0 | 43.79±87.59 | 43.79±87.59 | 49.04±98.09 |
| Escherichia-Shigella (21.91%) | 0.0 ± 0.0 | 1.08 ± 0.61 | 1.11 ± 0.63 | 0.0 ± 0.0 | 1.05 ± 0.49 | 0.0 ± 0.0 | 0.0 ± 0.0 | 0.0 ± 0.0 | 0.0 ± 0.0 |
| Helicobacter (0.22%) | 8.61 ± 9.29 | 8.63 ± 11.44 | 8.55 ± 11.12 | 1.22 ± 1.72 | 1.01 ± 1.50 | 10.39 ± 18.51 | 0.31 ± 0.62 | 0.31 ± 0.62 | 0.35 ± 0.70 |
| Lactobacillus (0.22%) | 13.94 ± 21.01 | 0.0 ± 0.0 | 14.73 ± 24.07 | 0.0 ± 0.0 | 0.0 ± 0.0 | 0.0 ± 0.0 | 0.0 ± 0.0 | 0.0 ± 0.0 | 0.0 ± 0.0 |
| Listeria (0.22%) | 16.39±24.66 | 17.57±28.97 | 17.29±28.28 | 0.0 ± 0.0 | 8.58±15.00 | 0.0 ± 0.0 | 0.0 ± 0.0 | 0.0 ± 0.0 | 0.0 ± 0.0 |
| Neisseria (0.22%) | 0.0 ± 0.0 | 0.0 ± 0.0 | 0.0 ± 0.0 | 12.68 ± 20.82 | 10.60 ± 17.99 | 0.0 ± 0.0 | 0.0 ± 0.0 | 0.0 ± 0.0 | 0.0 ± 0.0 |
| Porphyromonas (0%) | n/a | n/a | n/a | n/a | n/a | n/a | n/a | n/a | n/a |
| Pseudomonas (2.19%) | 0.0 ± 0.0 | 1.28 ± 0.65 | 0.0 ± 0.0 | 2.71 ± 1.19 | 2.05 ± 0.99 | 0.0 ± 0.0 | 0.47 ± 0.94 | 0.47 ± 0.94 | 0.53 ± 1.06 |
| Rhodobacter (21.91%) | 0.0 ± 0.0 | 0.0 ± 0.0 | 0.0 ± 0.0 | 1.26 ± 0.68 | 0.91 ± 0.44 | 4.25 ± 0.60 | 0.58 ± 0.30 | 0.58 ± 0.30 | 0.59 ± 0.29 |
| Salmonella (0%) | n/a | n/a | n/a | n/a | n/a | n/a | n/a | n/a | n/a |
| Staphylococcus (24.1%) | 1.41 ± 0.56 | 1.08 ± 0.26 | 1.08 ± 0.30 | 0.24 ± 0.19 | 0.18 ± 0.14 | 0.0 ± 0.0 | 0.36 ± 0.30 | 0.36 ± 0.30 | 0.37 ± 0.29 |
| Streptococcus (24.102%) | 1.62 ± 0.56 | 1.27 ± 0.30 | 1.29 ± 0.32 | 2.04 ± 0.29 | 1.52 ± 0.19 | 0.0 ± 0.0 | 0.47 ± 0.37 | 0.47 ± 0.37 | 0.42 ± 0.40 |

Staggered mock samples bei_stag n= 4 samples. n/a = Bacteria listed was not in the specified mock community. Values (mean or standard deviation) were rounded to two decimal places, and values < 0.005 were rounded to 0.0 (not true zero in every case). Taxon-specific agreement was defined as the observed/expected ratio and calculated as the observed relative abundance (%) / expected relative abundance (%) for each genus. A value of 1 indicates perfect agreement, a value under 0-0.999 indicates the actual relative abundance (%) is less than expected, and a value over 1 indicates the actual relative abundance (%) is higher than expected in the mock community for that individual taxon. Non-parametric tests were run to determine precision metric differences between V region (Kruskal-Wallis), reference databases (Kruskal-Wallis), and bioinformatics workflows (Wilcoxon Rank Sum), respectively, for each individual genus.
