# Supplementary material for: Multi-amplicon microbiome data analysis pipelines for mixed orientation sequences using QIIME2: Assessing reference database, variable region and pre-processing bias in classification of mock bacterial community samples
Source: PLoS One. 2023 Jan 13;18(1):e0280293. doi: 10.1371/journal.pone.0280293 (PMC9838852; doi:10.1371/journal.pone.0280293)
Supplement: S12 Table — Staggered mock samples zymo_stag n = 2. n/a = Bacteria listed was not in the specified mock community. Values (mean or standard deviation) were rounded to two decimal places, and values < 0.005 were rounded to 0.0 (not true zero in every case). Taxon-specific agreement was defined as the observed/expected ratio and calculated as the observed relative abundance (%) / expected relative abundance (%) for each genus. A value of 1 indicates perfect agreement, a value under 0–0.999 indicates the actual relative abundance (%) is less than expected, and a value over 1 indicates the actual relative abundance (%) is higher than expected in the mock community for that individual taxon. Non-parametric tests were run to determine precision metric differences between V region (Kruskal-Wallis), reference databases (Kruskal-Wallis), and bioinformatics workflows (Wilcoxon Rank Sum), respectively, for each individual genus. (DOCX) [file pone.0280293.s017.docx]

**Supplemental Table 12: Taxon-Specific Metrics by Mock Type**

**Staggered ZYMO Mock Bacterial Community Samples V2, V3, V4**

| **Genus (Expected**  **Abundance %)** | **Stag ZYMO V2 GG** | **Stag ZYMO V2 Silva** | **Stag ZYMO V2 RDP** | **Stag ZYMO V3 GG** | **Stag ZYMO V3 Silva** | **Stag ZYMO V3 RDP** | **Stag ZYMO V4 GG** | **Stag ZYMO V4 Silva** | **Stag ZYMO V4 RDP** |
| --- | --- | --- | --- | --- | --- | --- | --- | --- | --- |
| **CutPrimers** | | | | | | | | | |
| Acinetobacter (0%) | n/a | n/a | n/a | n/a | n/a | n/a | n/a | n/a | n/a |
| Actinomyces (0%) | n/a | n/a | n/a | n/a | n/a | n/a | n/a | n/a | n/a |
| Bacillus (17.4%) | 0.57 ± 0.27 | 0.48 ± 0.22 | 0.54 ± 0.26 | 1.37 ± 0.56 | 1.12 ± 0.46 | 0.00 ± 0.01 | 1.31 ± 0.78 | 0.76 ± 0.40 | 1.09 ± 0.63 |
| Bacteroides (0%) | n/a | n/a | n/a | n/a | n/a | n/a | n/a | n/a | n/a |
| Bifidobacterium (0%) | n/a | n/a | n/a | n/a | n/a | n/a | n/a | n/a | n/a |
| Clostridium (0%) | n/a | n/a | n/a | n/a | n/a | n/a | n/a | n/a | n/a |
| Cutibacterium/ Propionibacterium (0%) | n/a | n/a | n/a | n/a | n/a | n/a | n/a | n/a | n/a |
| Deinococcus (0%) | n/a | n/a | n/a | n/a | n/a | n/a | n/a | n/a | n/a |
| Enterococcus (9.9%) | 1.35 ± 0.09 | 1.14 ± 0.06 | 1.28 ± 0.08 | 2.83 ± 0.21 | 2.31 ± 0.17 | 3.12 ± 0.15 | 1.80 ± 0.09 | 1.07 ± 0.03 | 1.50 ± 0.05 |
| Escherichia-Shigella (10.1%) | 0.0 ± 0.0 | 1.53 ± 0.09 | 0.48 ± 0.00 | 0.0 ± 0.0 | 1.81 ± 0.02 | 2.44 ± 0.03 | 0.0 ± 0.0 | 1.70 ± 0.11 | 2.40 ± 0.30 |
| Helicobacter (0%) | n/a | n/a | n/a | n/a | n/a | n/a | n/a | n/a | n/a |
| Lactobacillus (18.4%) | 1.09 ± 0.46 | 0.92 ± 0.39 | 1.04 ± 0.43 | 0.44 ± 0.42 | 0.36 ± 0.34 | 0.0 ± 0.0 | 1.85 ± 0.82 | 1.12 ± 0.57 | 1.55 ± 0.71 |
| Listeria (14.1%) | 0.21 ± 0.05 | 0.18 ± 0.04 | 0.20 ± 0.05 | 0.54 ± 0.16 | 0.44 ± 0.13 | 0.60 ± 0.19 | 0.0 ± 0.0 | 0.29 ± 0.16 | 0.0 ± 0.0 |
| Neisseria (0%) | n/a | n/a | n/a | n/a | n/a | n/a | n/a | n/a | n/a |
| Porphyromonas (0%) | n/a | n/a | n/a | n/a | n/a | n/a | n/a | n/a | n/a |
| Pseudomonas (4.2%) | 4.68 ± 0.33 | 3.96 ± 0.23 | 4.46 ± 0.31 | 4.02 ± 0.49 | 3.29 ± 0.40 | 4.44 ± 0.43 | 2.94 ± 0.43 | 1.76 ± 0.39 | 2.46 ± 0.39 |
| Rhodobacter (0%) | n/a | n/a | n/a | n/a | n/a | n/a | n/a | n/a | n/a |
| Salmonella (10.4%) | 2.95 ± 0.13 | 2.50 ± 0.08 | 2.81 ± 0.12 | 0.0 ± 0.0 | 0.0 ± 0.0 | 0.0 ± 0.0 | 0.0 ± 0.0 | 1.85 ± 0.12 | 0.30 ± 0.01 |
| Staphylococcus (15.5%) | 0.22 ± 0.04 | 0.18 ± 0.03 | 0.21 ± 0.03 | 1.00 ± 0.01 | 0.82 ± 0.01 | 1.11 ± 0.02 | 0.84 ± 0.16 | 0.49 ± 0.06 | 0.0 ± 0.0 |
| Streptococcus (0%) | n/a | n/a | n/a | n/a | n/a | n/a | n/a | n/a | n/a |

**Staggered ZYMO Mock Bacterial Community Samples V67, V8, V9**

| **Genus (Expected Abundance %)** | **Stag ZYMO V67 GG** | **Stag ZYMO V67 Silva** | **Stag ZYMO V67 RDP** | **Stag ZYMO V8 GG** | **Stag ZYMO V8 Silva** | **Stag ZYMO V8 RDP** | **Stag ZYMO V9 GG** | **Stag ZYMO V9 Silva** | **Stag ZYMO V9 RDP** |
| --- | --- | --- | --- | --- | --- | --- | --- | --- | --- |
| **CutPrimers** | | | | | | | | | |
| Acinetobacter (0%) | n/a | n/a | n/a | n/a | n/a | n/a | n/a | n/a | n/a |
| Actinomyces (0%) | n/a | n/a | n/a | n/a | n/a | n/a | n/a | n/a | n/a |
| Bacillus (17.4%) | 1.15 ± 0.64 | 0.74 ± 0.38 | 0.93 ± 0.48 | 0.05 ± 0.01 | 0.04 ± 0.01 | 0.0 ± 0.0 | 0.0 ± 0.0 | 0.0 ± 0.0 | 0.0 ± 0.0 |
| Bacteroides (0%) | n/a | n/a | n/a | n/a | n/a | n/a | n/a | n/a | n/a |
| Bifidobacterium (0%) | n/a | n/a | n/a | n/a | n/a | n/a | n/a | n/a | n/a |
| Clostridium (0%) | n/a | n/a | n/a | n/a | n/a | n/a | n/a | n/a | n/a |
| Cutibacterium/  Propionibacterium (0%) | n/a | n/a | n/a | n/a | n/a | n/a | n/a | n/a | n/a |
| Deinococcus (0%) | n/a | n/a | n/a | n/a | n/a | n/a | n/a | n/a | n/a |
| Enterococcus (9.9%) | 1.86 ± 0.01 | 0.0 ± 0.0 | 0.0 ± 0.0 | 2.29 ± 0.09 | 1.56 ± 0.10 | 0.0 ± 0.0 | 0.0 ± 0.0 | 0.0 ± 0.0 | 0.0 ± 0.0 |
| Escherichia-Shigella (10.1%) | 0.0 ± 0.0 | 3.45 ± 0.33 | 4.32 ± 0.43 | 0.0 ± 0.0 | 2.61 ± 0.01 | 0.0 ± 0.0 | 0.0 ± 0.0 | 0.0 ± 0.0 | 0.0 ± 0.0 |
| Helicobacter (0%) | n/a | n/a | n/a | n/a | n/a | n/a | n/a | n/a | n/a |
| Lactobacillus (18.4%) | 1.92 ± 1.13 | 1.26 ± 0.79 | 1.58 ± 0.98 | 0.13 ± 0.09 | 0.09 ± 0.06 | 0.0 ± 0.0 | 0.0 ± 0.0 | 0.0 ± 0.0 | 0.0 ± 0.0 |
| Listeria (14.1%) | 0.80 ± 0.47 | 0.51 ± 0.28 | 0.64 ± 0.35 | 0.0 ± 0.0 | 0.39 ± 0.13 | 0.0 ± 0.0 | 0.0 ± 0.0 | 0.0 ± 0.0 | 0.0 ± 0.0 |
| Neisseria (0%) | n/a | n/a | n/a | n/a | n/a | n/a | n/a | n/a | n/a |
| Porphyromonas (0%) | n/a | n/a | n/a | n/a | n/a | n/a | n/a | n/a | n/a |
| Pseudomonas (4.2%) | 0.0 ± 0.0 | 2.90 ± 0.28 | 0.0 ± 0.0 | 5.88 ± 0.10 | 4.01 ± 0.17 | 0.0 ± 0.0 | 18.80 ± 7.09 | 18.80 ± 7.09 | 18.80 ± 7.09 |
| Rhodobacter (0%) | n/a | n/a | n/a | n/a | n/a | n/a | n/a | n/a | n/a |
| Salmonella (10.4%) | 0.0 ± 0.0 | 0.0 ± 0.0 | 0.0 ± 0.0 | 4.54 ± 0.36 | 3.09 ± 0.16 | 0.0 ± 0.0 | 0.0 ± 0.0 | 0.0 ± 0.0 | 0.0 ± 0.0 |
| Staphylococcus (15.5%) | 0.97 ± 0.20 | 0.62 ± 0.11 | 0.13 ± 0.02 | 0.14 ± 0.04 | 0.10 ± 0.03 | 0.0 ± 0.0 | 1.36 ± 1.92 | 1.36 ± 1.92 | 1.36 ± 1.92 |
| Streptococcus (0%) | n/a | n/a | n/a | n/a | n/a | n/a | n/a | n/a | n/a |

Staggered mock samples zymo_stag n=2. n/a = Bacteria listed was not in the specified mock community. Values (mean or standard deviation) were rounded to two decimal places, and values < 0.005 were rounded to 0.0 (not true zero in every case). Taxon-specific agreement was defined as the observed/expected ratio and calculated as the observed relative abundance (%) / expected relative abundance (%) for each genus. A value of 1 indicates perfect agreement, a value under 0-0.999 indicates the actual relative abundance (%) is less than expected, and a value over 1 indicates the actual relative abundance (%) is higher than expected in the mock community for that individual taxon. Non-parametric tests were run to determine precision metric differences between V region (Kruskal-Wallis), reference databases (Kruskal-Wallis), and bioinformatics workflows (Wilcoxon Rank Sum), respectively, for each individual genus.
